# Palladium-catalyzed dehydrogenative C–H cyclization for isoindolinone synthesis†

**DOI:** 10.1039/d1ra04661f

**Published:** 2021-08-08

**Authors:** Masahiro Abe, Kaho Ueta, Saki Tanaka, Tetsutaro Kimachi, Kiyofumi Inamoto

**Affiliations:** School of Pharmacy and Pharmaceutical Sciences, Mukogawa Women's University 11-68, 9-Bancho, Koshien, Nishinomiya Hyogo 663-8179 Japan abe_111@mukogawa-u.ac.jp inamoto@mukogawa-u.ac.jp

## Abstract

In this paper Pd-catalyzed intramolecular dehydrogenative C(sp^3^)–H amidation for the synthesis of isoindolinones is described. This method features the use of a Pd/C catalyst and the addition of a stoichiometric amount of oxidant is not necessary. A mechanistic study suggested the possible formation of H_2_ gas during the reaction.

## Abstract 2

Pd-catalyzed intramolecular dehydrogenative C(sp^3^)–H amidation for the synthesis of isoindolinones was developed. Use of Pd/C as a catalyst enables the desired cyclization to proceed smoothly without adding any stoichiometric oxidants.

## Graphical abstract

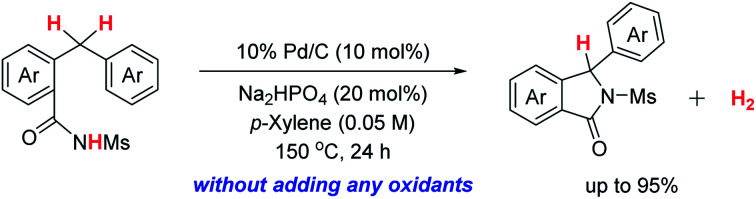

The isoindolinone scaffold occurs frequently in numerous biologically active compounds ranging from designed medicinal agents to natural products, thus constituting an extremely important class of heterocycles.^1,2^ Although a number of synthetic methods exist for the preparation of isoindolinones,^3^ the development of more general, versatile, and efficient procedures to construct the isoindolinone framework is still an ongoing, intensive research area.

Among a variety of synthetic strategies for isoindolinones, methods that make use of transition metal-catalyzed C–H functionalization represent a remarkable approach.^4^ In this research area, catalytic oxidative annulation of N-substituted benzamides and appropriate coupling partners (*e.g.* alkenes, alkynes, isocyanides, diazo compounds) has been extensively studied, most of which employ transition metals such as rhodium,^4*d*,*g*,*h*,*n*,*o*^ ruthenium,^4*l*^ cobalt,^4*a*,*i*,*j*,*k*^ and palladium.^4*b*,*m*^ These precedents often require the use of stoichiometric oxidants or prefunctionalized coupling partners to make the process catalytic.

On the other hand, an approach that involves intramolecular C–H cyclization of benzamide derivatives exploiting a transition metal catalyst could provide another efficient, facile, and direct access to isoindolinones, although less attention has been paid to such a process (Scheme 1). Kondo *et al.* previously reported C(sp^3^)–H aminative cyclization of 2-methyl-*N*-arylbenzamides in the presence of a copper catalyst along with a stoichiometric amount of di-*tert*-butyl peroxide as an oxidant, which resulted in the formation of 3-unsubstituted isoindolinones (Scheme 1a).^4*c*^ Bedford's group also developed a copper-based catalytic system composed of 20 mol% of Cu(OTf)_2_ and 2 equiv. of PhI(OAc)_2_ that successfully effected the intramolecular benzylic C–H sulfamidation of 2-benzyl-*N*-tosylbenzamides for the synthesis of isoindolinones (Scheme 1b).^4*f*^ In both cases, the choice of an oxidant should be crucial for the successful construction of the isoindolinone ring.

**Scheme 1:**
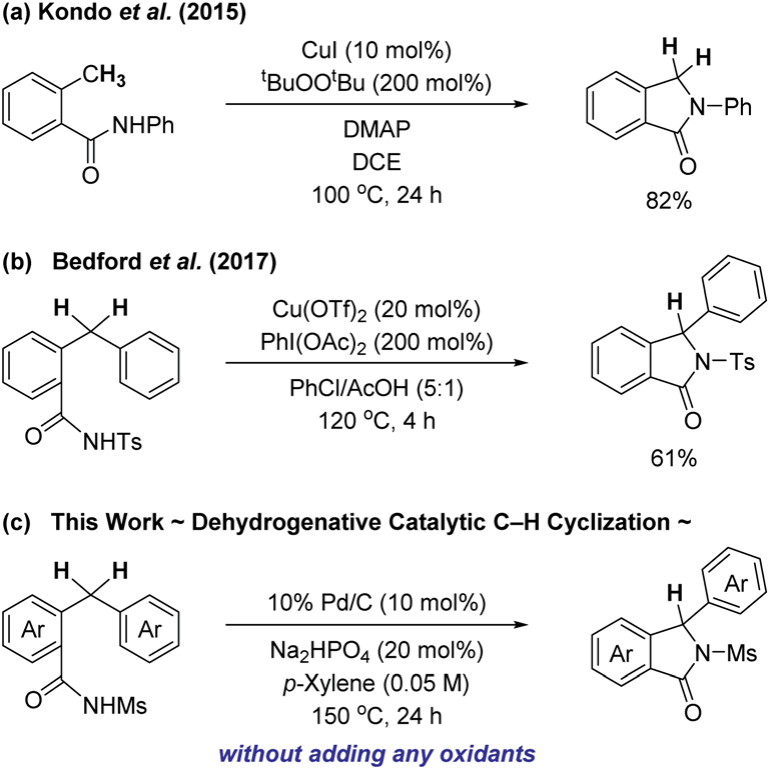
C–H cyclization strategies for isoindolinone synthesis.

Herein, we describe a catalytic system that enables cyclization of 2-benzyl-*N*-mesylbenzamides leading to isoindolinone derivatives, in which benzylic C(sp^3^)–H functionalization in the presence of a palladium catalyst smoothly occurs. It is particularly noteworthy to mention that any stoichiometric oxidants are not necessary for our isoindolinone synthesis. The key to success is the use of Pd/C as a catalyst. In this dehydrogenative process, an only detectable by-product was H_2_, which should also be an appealing feature of the process.

Based on our fruitful results of the heterocycles synthesis *via* transition metal-catalyzed C–H cyclization,^5^ our investigation began by examining the conversion of *N*-tosyl-protected benzamide 1a to the corresponding isoindolinone 2a. During the screening studies utilizing a range of transition metal catalysts as well as various oxidants, it was found that 2a did produce even in the absence of an oxidant when Pd/C was used as a catalyst. Indeed, the use of 10 mol% of Pd/C along with 20 mol% of KOAc enabled the desired cyclization process, providing 2a in 42% yield (Table 1). Intrigued by this unexpected oxidant-free process, the effect of the protecting group on the nitrogen atom of an amide moiety was briefly evaluated. We are pleased to find that the reaction of 1f possessing a mesyl group (–Ms) efficiently occurred and the isoindolinone 2f was obtained in fairly good yield (75%).^6^

**Table 1:** Effect of protecting group^a^^,^^b^

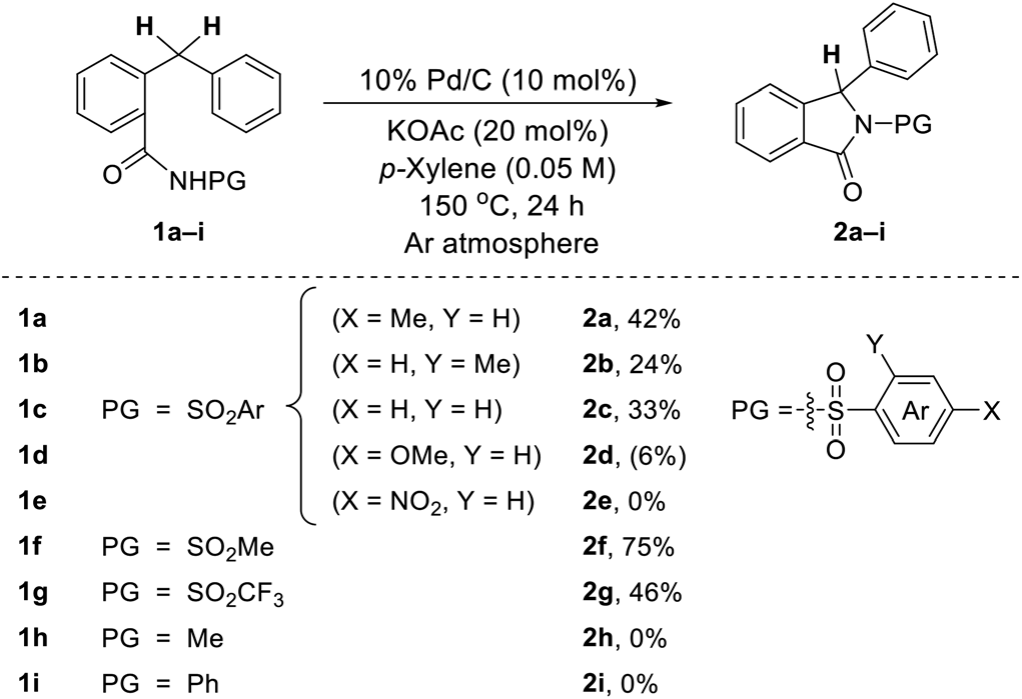

a Reactions were run on a 0.25 mmol scale.

b Isolated yields (yield determined by ^1^H NMR using an internal standard in parentheses).

Settled in –Ms for the protecting group, further examination of the reaction parameters was performed employing 1f (Table 2). Use of Na_2_HPO_4_ gave a slightly better result (entry 2), while reactions with other bases led to lower yields (entry 3). Among a variety of solvents tested, *p*-xylene was found to be the best (entry 4). Varying the concentration of the reaction did not enhance the process (entry 5). Performing the reaction under an oxygen atmosphere dreadfully diminished the yield (entry 6). In contrast, use of the degassed conditions by means of Ar bubbling afforded the desired 2f with excellent yield (entry 7). In addition, it was found that the yield decreased when the reaction was carried out in the absence of a base (entry 8).^7,8^

**Table 2:** Optimization of reaction parameters^a^

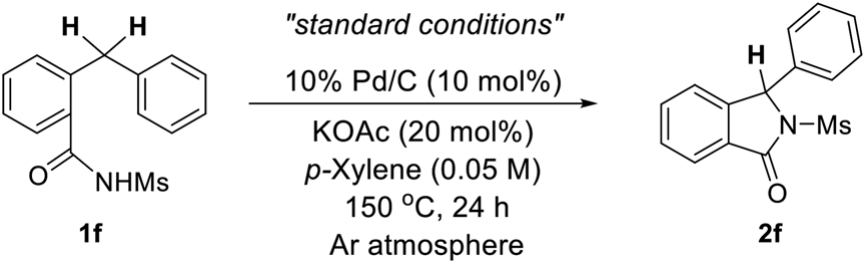		
Entry	Variation from “standard conditions"	Yield^b^^,^^c^ (%)
1	None	(75)
2	Na_2_HPO_4_ instead of KOAc	86 (80)
3	NaOAc, LiOAc, K_2_CO_3_ or Cs_2_CO_3_ instead of KOAc	61–70
4	DMA, DMI or DMSO instead of *p*-xylene	0–28
5	0.25 M instead of 0.05 M	67
6^d^	O_2_ atmosphere instead of Ar atmosphere	4
7^d^	Degassed (bubbling with Ar)	92 (86)
8^d^	In the absence of KOAc	72

a Reactions were run on a 0.25 mmol scale.

b Yields were determined by ^1^H NMR using an internal standard.

c Isolated yields in parentheses.

d Na_2_HPO_4_ was used instead of KOAc.

Our new method for isoindolinone synthesis can be applied to cyclization of a wide range of substrates (Table 3). Substituents such as an electron donating methyl or methoxy group as well as an electron withdrawing halogen atom on the benzene ring are well tolerated under the reaction conditions, and the corresponding products 2j–l are successfully obtained. It is also noteworthy that heterocycles such as thiophene and indoles are compatible during the process (2n–q). Remarkably, benzamides 1o–q possessing an indole nucleus that can be easily oxidable were suitable under our oxidant-free conditions.^9^ An attempt to obtain 3-alkylisoindolinone 2r unfortunately failed. On the other hand, the reaction of 1f can be easily scaled up: in this case, 2f was obtained in 92% yield.^10^

**Table 3:** Substrate scope^a^^,^^b^

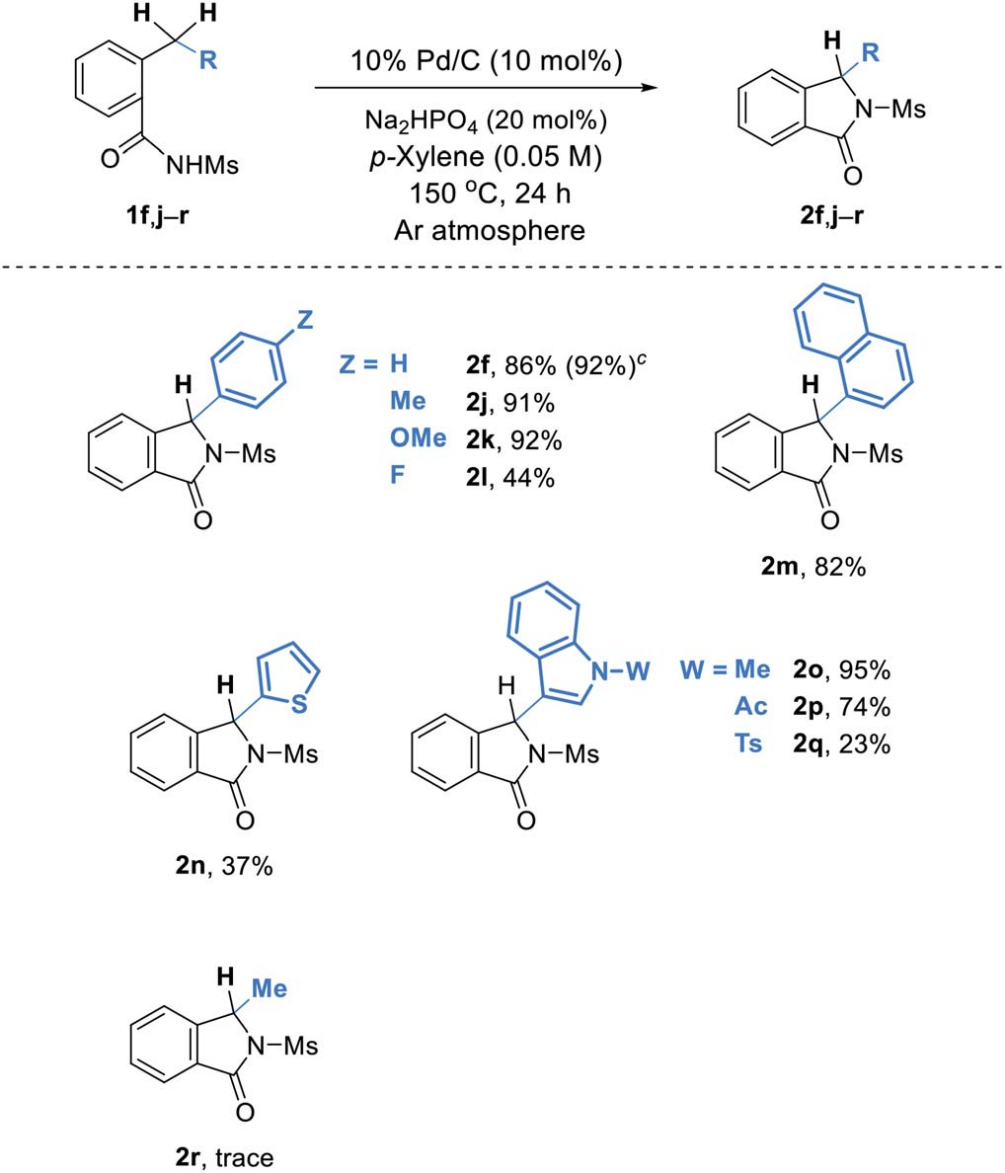

a Reactions were run on a 0.25 mmol scale.

b Isolated yields.

c 1 mmol scale.

To further demonstrate the synthetic utility of the method we have developed, several transformations of isoindolinone 2f obtained were carried out (Scheme 2). Deprotection of a mesyl group with AIBN and Bu_3_SnH afforded the N-free isoindolinone 3 in high yield. Reduction of the carbonyl group of 2f using LiAlH_4_ also successfully preceded, giving rise to isoindoline 4 in excellent yield (97%).

**Scheme 2:**
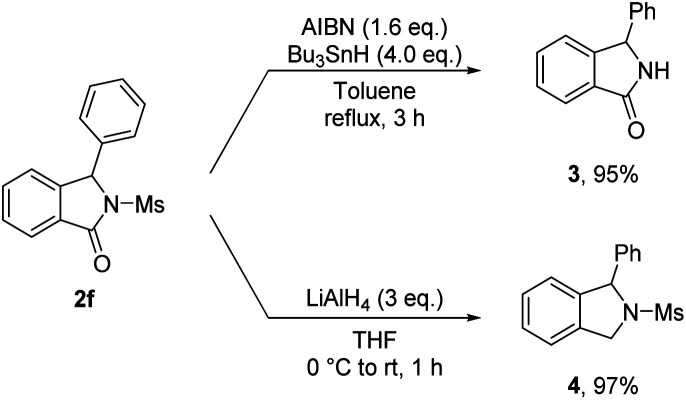
Further transformation of isoindolinone 2f.

To gain insight into the reaction mechanism of the process, C–H cyclization of 1f in the presence of 0.5 equiv. of methyl cinnamate (5) was performed under the optimized conditions (Scheme 3). In addition to isoindolinone 2f, methyl 3-phenylpropanoate (6) was observed in 32% NMR yield, implying that the formation of H_2_ gas is possibly occurring during the reaction.^11^

**Scheme 3:**
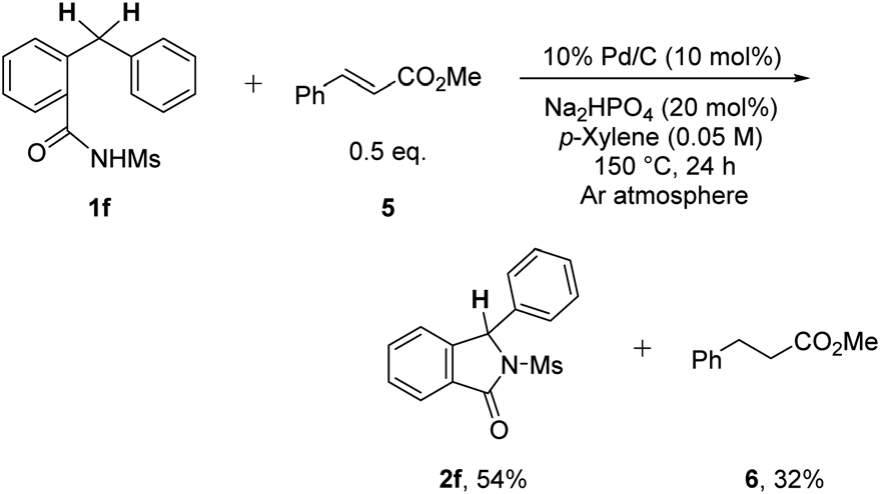
Mechanistic studies.

On the basis of the finding above, a tentative reaction pathway shown in Scheme 4 is proposed. The reaction is likely initiated by the coordination of the nitrogen atom of an amide moiety to give complex A. Subsequently, the insertion of Pd(0) into the benzylic C(sp^3^)–H bond leading to the formation of six-membered palladacycle B accompanied with the evolution of H_2_ gas and the following reductive elimination process affords the desired isoindolinone 2.

**Scheme 4:**
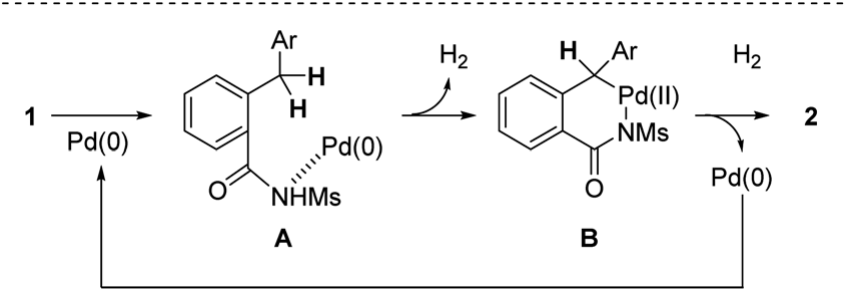
Plausible reaction mechanism.

In summary, we have developed intramolecular Pd-catalyzed dehydrogenative C(sp^3^)–H amidation for isoindolinone synthesis. The addition of oxidants is not necessary and the Pd/C catalyst along with a catalytic amount (20 mol%) of base is the only reagents required for this C–H cyclization. The method developed provides a simple, facile, and efficient access to a biologically important isoindolinone nucleus. Further studies to broaden the substrate scope of the process as well as to improve the catalytic efficiency are vigorously underway in our laboratory.

## Conflicts of interest

The authors declare no competing financial interest.

## Supplementary Material

RA-011-D1RA04661F-s001
