# Supplementary material for: Palladium-catalyzed dehydrogenative C–H cyclization for isoindolinone synthesis
Source: RSC Adv. 2021 Aug 8;11(43):26988–91. doi: 10.1039/d1ra04661f (PMC9037686; doi:10.1039/d1ra04661f)

Electronic Supplementary Material (ESI) for RSC Advances.
This journal is © The Royal Society of Chemistry 2021

Electronic Supplementary Information

Palladium-Catalyzed Dehydrogenative C–H Cyclization for Isoindolinone Synthesis

Masahiro Abe,* Kaho Ueta, Saki Tanaka, Tetsutaro Kimachi, Kiyofumi Inamoto*

School of Pharmacy and Pharmaceutical Sciences, Mukogawa Women's University
11-68, 9-Bancho, Koshien, Nishinomiya, Hyogo 663-8179, Japan

e-mail: abe_111@mukogawa-u.ac.jp, inamoto@mukogawa-u.ac.jp

1. General Comments **P.2**

2. Materials **P.2**

3. Details of Optimization Studies **P.3**

4. Spectroscopic and Analytical Data **P.5**

5. References **P.21**

6. ^{1}H-, ^{13}C- and ^{19}F-NMR Spectra **P.22**

7. Mechanistic Studies **P.63**

1. General Comments

Melting points were measured with a AS ONE Corporation melting temperature measurement device (ATM-02) and uncorrected. IR spectra were recorded on a SHIMADZU IRAffinity-1. NMR data were recorded on either a JEOL JNM-ECP400 spectrometer (400 MHz) or a JEOL ECA500 spectrometer (500 MHz). Chemical shifts are expressed in δ (parts per million, ppm) values and coupling constants are expressed in hertz (Hz). ^{1}H NMR spectra were referenced to $(CH_3)_4Si$ (TMS) as an internal standard or to a residual proton signal in deuterated solvent ($CDCl_3$: 7.26 ppm, DMSO-d_6: 2.50 ppm, acetone-d_6: 2.05 ppm). 1,1,2-Trichloroethane was used as an internal standard. ^{13}C NMR spectra were referenced to a residual proton signal in deuterated solvent ($CDCl_3$: 77.16 ppm, DMSO-d_6: 39.52 ppm, acetone-d_6: 206.26 ppm and 29.84 ppm). ^{19}F NMR spectra were referenced to 4-fluorotoluene as an internal standard (−118.0 ppm). The following abbreviations are used: s = singlet, d = doublet, t = triplet, q = quartet, sext = sextet, dd, = double doublet, dt = double triplet, td = triple doublet, ddd, = double double doublet, m = multiplet, and brs = broad signal. Mass spectra and high resolution mass spectra were measured on a JEOL JMS-700 instrument. Chromatographic separations were achieved on silica gel column (Wakosil® C-200, 64 − 210 µm).

2. Materials

All commercially available materials including palladium on activated charcoal (Sigma–Aldrich Co., #75990) and anhydrous *p*-xylene (Sigma–Aldrich Co., #296333, ≥99%) were purchased from Sigma–Aldrich Co., Tokyo Chemical Industry Co. and Wako Pure Chemical Industries, and were used as received. Test tubes with screws (IWAKI, TST SCR 25-150) were used for isoindolinone synthesis. Starting materials **1h** and **1i** were prepared according to the literature (acid chloride formation and subsequent addition of amine).[1,2] 2-(4-Methylbenzyl)benzoic acid,[3] 2-(4-methoxylbenzyl)benzoic acid,[4] 2-(4-fluorobenzyl)benzoic acid,[5] 2-(thiophen-2-ylmethyl)benzoic acid[5] were prepared according to the literature.

3. Details of Optimization Studies

Pd Catalyst Screening

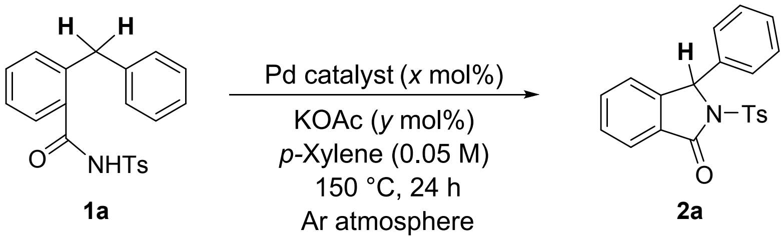

entry	Pd catalyst (*x* mol%)	KOAc (*y* mol%)	yield (%)
1	$Pd(PPh_3)_4$ (25 mol%)	100	0
2	Pd_2dba_3 (12.5 mol%)	100	27
3	10% Pd/C (25 mol%)	100	43
**4**	**10% Pd/C (10 mol%)**	**20**	**43**

Yields were determined by ^{1}H NMR using an internal standard.

Solvent Screening

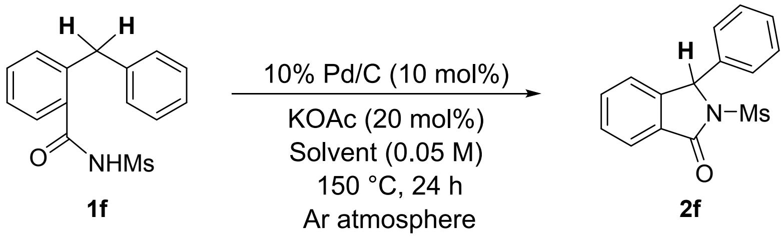

entry	Solvent	yield (%)[a,b]
**1**	***p*-Xylene**	**83 (75)**
2	*o*-Xylene	83
3	*m*-Xylene	72
4	mesityene	38
5	DMA	28
6	DMSO	0
7	DMI	0

[a] Yields were determined by ^{1}H NMR using an internal standard.
[b] Isolated yield in parentheses.

Base Screening

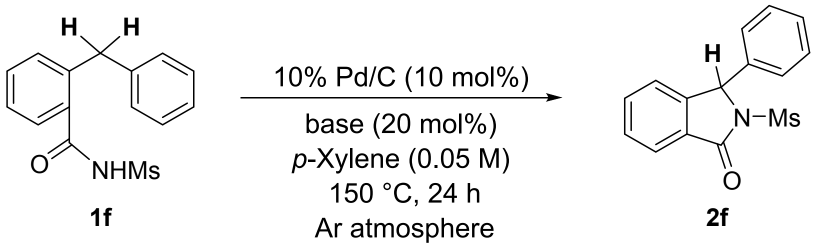

1f **2f**

entry	Base	yield (%)[a,b]
1	KOAc	83
2	NaOAc	70
3	LiOAc	68
4	K_2CO_3	61
5	$KHCO_3$	77
6	Cs_2CO_3	64
**7**	**Na_2HPO_4**	**86 (80)**
8	Na_3PO_4	68
9	K_2HPO_4	81
10	K_3PO_4	71
11	pyridine	84
12	NEt_3	80

[a] The yields were determined by ^{1}H NMR using an internal standard.
[b] Isolated yield in parentheses.

4. Spectroscopic and Analytical Data

Preparation of 2-Benzyl-*N*-Tosylbenzamide (1a)[6)]

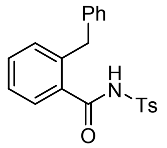

p-Toluenesulfonyl isocyanate (0.72 mL, 4.7 mmol) was added to a solution of 2-benzylbenzoic acid (1.0 g, 4.7 mmol) in THF (10 mL) and the mixture was stirred at room temperature for 10 min. Triethylamine (0.66 mL, 4.7 mmol) was added dropwise to the solution with evolution of gas, and the reaction mixture was stirred at rt overnight. 2M HCl (5 mL) was added and the mixture was extracted with AcOEt (10 mL × 3). The combined organic phase was washed with brine (10 mL) and dried over $MgSO_4$. The solvent was removed under a reduced pressure and the resulting solid was purified by silica gel flash column chromatography eluting with hexane/AcOEt and recrystallization from hexane/AcOEt to give 2-benzyl-*N*-tosylbenzamide (**1a**) as colorless needles (77% yield, 1.33 g, 3.63 mmol).

mp 156 – 157 ℃ (recrystallized from hexane/AcOEt, lit.[6)] mp 156 – 157 ºC).

^{1}H NMR (400 MHz, $CDCl_3$/TMS) δ 8.32 (brs, 1H), 7.94 (d, *J* = 8.4 Hz, 2H), 7.44 – 7.35 (m, 2H), 7.32 (d, *J* = 8.1 Hz, 2H), 7.26 – 7.18 (m, 2H), 7.18 – 7.08 (m, 3H), 6.99 – 6.90 (m, 2H), 4.06 (s, 2H), 2.45 (s, 3H).

^{13}C{^{1}H} NMR (101 MHz, $CDCl_3$/TMS) δ 166.3, 145.2, 140.7, 140.2, 135.7, 132.8, 132.0, 131.7, 129.7, 128.9, 128.7, 128.7, 127.8, 126.7, 126.4, 38.5, 21.8.

IR (neat): 3084, 3028, 2860, 1672, 1593, 1493, 1441, 1350, 1256, 1167, 1126, 1076, 883, 847, 822, 777, 741 *cm^{-1}*

LRMS (EI) *m/z*: 365 (M^+).

HRMS: Calcd. for $C_{21}H_{19}NO_3S$: 365.1086, found: 365.1086.

Rf = 0.13 (hexane/AcOEt: 4/1)

General Procedure for Preparation of Sulfonimide (1b-r)

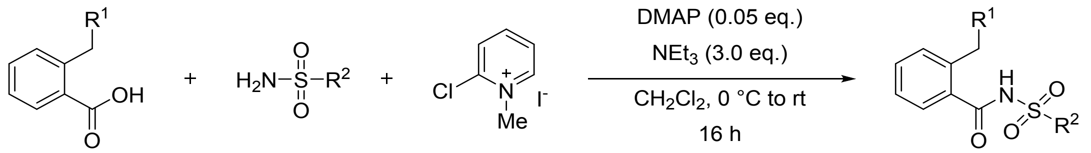

A corresponding benzoic acid (1.0 eq.), a corresponding sulfonamide (2.0 eq.), 2-chloro-1-methylpyridinium iodide (1.2 eq.) and DMAP (0.05 eq.) were dissolved in CH_2Cl_2 (0.25 M) at rt. After stirring for 5 min, triethylamine (3.0 eq.) was slowly added at 0 °C. The reaction was warmed to rt and stirred for 16 h. The reaction was quenched with 1M HCl aq. and taken up in AcOEt. The organic layer was separated, washed with H_2O and brine, dried over $MgSO_4$, filtered, and concentrated *in vacuo*. The residue was purified by silica gel flash column chromatography eluting with hexane/AcOEt to afford the desired product.

2-Benzyl-*N*-(*o*-tolylsulfonyl)benzamide (1b)

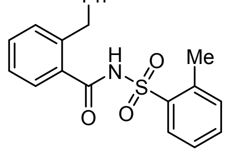

74% yield (1.27 g, 3.47 mmol) from 2-benzylbenzoic acid (1.0 g, 4.71 mmol).

colorless solid, **mp** 112 °C (recrystallized from hexane/AcOEt)

^{1}H NMR (500 MHz, $CDCl_3$/TMS) δ 8.61 (brs, 1H), 8.16 (d, *J* = 8.0 Hz, 1H), 7.51 (t, *J* = 7.5 Hz, 1H), 7.46 (d, *J* = 7.5 Hz, 1H), 7.42 – 7.34 (m, 2H), 7.30 – 7.19 (m, 3H), 7.18 – 7.10 (m, 3H), 6.98 – 6.92 (m, 2H), 4.07 (s, 2H), 2.52 (s, 3H).

^{13}C{^{1}H} NMR (126 MHz, $CDCl_3$/TMS) δ 166.4, 141.0, 140.2, 137.8, 136.8, 134.0, 132.6, 132.5, 132.1, 131.8, 131.6, 128.9, 128.7, 127.9, 126.7, 126.6, 126.4, 38.5, 20.4.

IR (neat): 3142, 3026, 2970, 2853, 1674, 1435, 1420, 1348, 1252, 1165, 1125, 1074, 1061, 891, 851, 777, 737, 689, 664, 588, 567 cm^{-1}

LRMS (EI) m/z 365 $(M)^+$

HRMS (EI) calcd for $(M)^+$ $C_{21}H_{19}NO_3S$:365.1086, found: 365.1090.

Rf = 0.29 (hexane/AcOEt: 2/1)

2-Benzyl-*N*-(phenylsulfonyl)benzamide (1c)

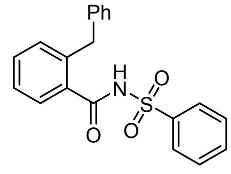

86% yield (1.42 g, 4.04 mmol) from 2-benzylbenzoic acid (1.0 g, 4.71 mmol).

colorless solid, **mp** 97 – 98 °C (recrystallized from hexane/AcOEt)

^{1}H NMR (500 MHz, $CDCl_3$/TMS) δ 8.39 (brs, 1H), 8.06 (d, *J* = 7.5 Hz, 2H), 7.68 – 7.62 (m, 1H), 7.54 (t, *J* = 7.9 Hz, 2H), 7.43 (d, *J* = 7.8 Hz, 1H), 7.39 (td, *J* = 7.6, 1.4 Hz, 1H), 7.27 – 7.19 (m, 2H), 7.18 – 7.09 (m, 3H), 6.99 – 6.91 (m, 2H), 4.05 (s, 2H).

^{13}C{^{1}H} NMR (126 MHz, $CDCl_3$/TMS) δ 166.4, 140.7, 140.2, 138.7, 134.1, 132.7, 132.1, 131.7, 129.1, 128.9, 128.7, 128.7, 127.8, 126.7, 126.4, 38.6.

IR (neat): 3312, 3215, 3057, 3030, 1707, 1688, 1418, 1404, 1331, 1169, 1090, 1070, 1038, 893, 845, 760, 731, 700, 683, 615, 569 cm^{-1}

LRMS (EI) m/z 351 $(M)^+$

HRMS (EI) calcd for $(M)^+$ $C_{20}H_{17}NO_3S$: 351.0929, found: 351.0926.

Rf = 0.19 (hexane/AcOEt: 2/1)

2-Benzyl-*N*-[(4-methoxyphenyl)sulfonyl]benzamide (1d)

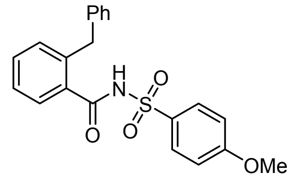

72% yield (1.29 g, 3.38 mmol) from 2-benzylbenzoic acid (1.0 g, 4.71 mmol).

colorless solid, **mp** 150 °C (recrystallized from hexane/AcOEt)

^{1}H NMR (500 MHz, $CDCl_3$/TMS) δ 8.22 (brs, 1H), 8.04 – 7.96 (m, 2H), 7.45–7.36 (m, 2H), 7.28 – 7.19 (m, 2H), 7.18 – 7.10 (m, 3H), 7.02 – 6.92 (m, 4H), 4.07 (s, 2H), 3.89 (s, 3H).

^{13}C{^{1}H} NMR (126 MHz) δ 166.4, 164.2, 140.6, 140.2, 132.9, 132.0, 131.7, 131.1, 130.1, 128.9, 128.7, 127.7, 126.8, 126.4, 114.3, 55.9, 38.6.

IR (neat): 3065, 3026, 2857, 1670, 1495, 1437, 1350, 1252, 1159, 1076, 1018, 885, 843, 775, 739, 698, 662, 556 cm^{-1}

LRMS (EI) m/z 381 $(M)^+$

HRMS (EI) calcd for $(M)^+$ $C_{21}H_{19}NO_4S$: 381.1035, found: 381.1032.

Rf = 0.26 (hexane/AcOEt: 2/1)

2-Benzyl-*N*-[(4-nitrophenyl)sulfonyl]benzamide (1e)

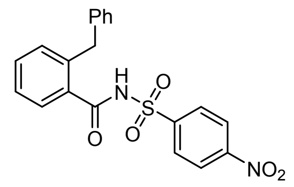

15% yield (241.6 mg, 0.61 mmol) from 2-benzylbenzoic acid (0.849 g, 4.0 mmol).

colorless solid, **mp** 163 – 164 °C (recrystallized from hexane/AcOEt)

^{1}H NMR (500 MHz, DMSO-d_6) δ 8.45 – 8.39 (m, 2H), 8.22 – 8.15 (m, 2H), 7.51 (dd, *J* = 7.7, 1.4 Hz, 1H), 7.47 (td, *J* = 7.6, 1.4 Hz, 1H), 7.36 – 7.28 (m, 2H), 7.12 – 7.03 (m, 3H), 6.90 – 6.83 (m, 2H), 3.97 (s, 2H).

^{13}C{^{1}H} NMR (126 MHz, DMSO-d_6) δ 167.8, 150.2, 144.4, 140.2, 139.8, 132.6, 131.6, 131.0, 129.2, 128.5, 128.2, 128.1, 126.2, 125.8, 124.3, 37.3.

IR (neat): 3244, 3107, 3024, 1715, 1522, 1431, 1352, 1234, 1175, 1092, 1057, 1045, 856, 729, 685, 604, 561 *cm^{-1}*

LRMS (EI) m/z 396 (M)$^+$

HRMS (EI) calcd for (M)$^+$ $C_{20}H_{16}N_2O_5S$: 396.0780, found: 396.0786.

Rf = 0.09 (hexane/AcOEt: 1/1)

2-Benzyl-*N*-(methylsulfonyl)benzamide (1f)

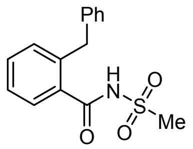

64% yield (1.74 g, 6.02 mmol) from 2-benzylbenzoic acid (2.0 g 9.42 mmol).

colorless solid, **mp** 117 – 118 °C (recrystallized from hexane/AcOEt)

^{1}H NMR (500 MHz, $CDCl_3$/TMS) δ 8.26 (brs, 1H), 7.52 – 7.42 (m, 2H), 7.35 – 7.28 (m, 2H), 7.27 – 7.22 (m, 2H), 7.17 (t, *J* = 7.4 Hz, 1H), 7.10 (d, *J* = 7.1 Hz, 2H), 4.26 (s, 2H), 3.03 (s, 3H).

^{13}C{^{1}H} NMR (126 MHz, $CDCl_3$/TMS) δ 167.5, 140.9, 140.5, 132.5, 132.3, 132.2, 129.1, 128.7, 128.0, 127.0, 126.5, 41.2, 39.2.

IR (neat): 3242, 3046, 3028, 1682, 1420, 1396, 1341, 1327, 1242, 1161, 1119, 1065, 972, 881, 847, 779, 745, 731, 702, 610 *cm^{-1}*

LRMS (EI) m/z 289 (M)$^+$

HRMS (EI) calcd for (M)$^+$ $C_{15}H_{15}NO_3S$: 289.0773, found: 289.0771.

Rf = 0.32 (hexane/AcOEt: 2/1)

2-Benzyl-*N*-[(trifluoromethyl)sulfonyl]benzamide (1g)

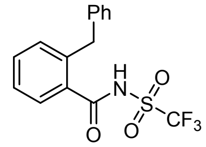

73% yield (751.1 mg, 2.19 mmol) from 2-benzylbenzoic acid (636.7 mg, 3.0 mmol).

colorless solid, **mp** 118 °C (recrystallized from hexane/AcOEt)

^{1}H NMR (400 MHz, $CDCl_3$/TMS) δ 7.56 – 7.48 (m, 2H), 7.39 – 7.32 (m, 2H), 7.30 – 7.24 (m, 2H), 7.23 – 7.17 (m, 1H), 7.14 – 7.08 (m, 2H), 4.23 (s, 2H).

^{13}C{^{1}H} NMR (101 MHz, $CDCl_3$/TMS) δ 164.7, 141.5, 139.6, 133.1, 132.3, 131.3, 129.0, 128.8, 127.99, 127.0, 126.7, 119.21 (q, *J* = 322.4 Hz), 38.8.

^{19}F NMR (471 MHz, $CDCl_3$) δ –74.7.

IR (neat): 3146, 3030, 2963, 2922, 2887, 2814, 1701, 1491, 1447, 1381, 1213, 1198, 1136, 1034, 891, 866, 733, 637, 602 *cm^{-1}*

LRMS (EI) m/z 343(M)$^+$

HRMS (EI) calcd for (M)$^+$ $C_{15}H_{12}F_3NO_3S$: 343.0490, found: 43.0490.

Rf = 0.31 (hexane/AcOEt: 1/1)

2-(4-Methylbenzyl)-*N*-(methylsulfonyl)benzamide (1j)

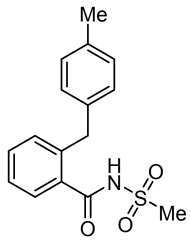

83% yield (558.3 mg, 1.84 mmol) from 2-(4-methylbenzyl)benzoic acid (500.0 mg, 2.21 mmol).

colorless solid, **mp** 131 – 132 °C (recrystallized from hexane/AcOEt)

^{1}H NMR (500 MHz, $CDCl_3$/TMS) δ 7.97 (brs, 1H), 7.53 – 7.44 (m, 2H), 7.36 – 7.28 (m, 2H), 7.07 (d, *J* = 8.0 Hz, 2H), 7.00 (d, *J* = 8.0 Hz, 2H), 4.23 (s, 2H), 3.10 (s, 3H), 2.29 (s, 3H).

^{13}C{^{1}H} NMR (126 MHz, $CDCl_3$/TMS) δ 167.4, 141.1, 137.3, 136.1, 132.5, 132.3, 132.2, 129.4, 129.0, 127.9, 127.0, 41.3, 38.8, 21.1.

IR (neat): 3302, 3011, 1703, 1514, 1497, 1418, 1402, 1327, 1238, 1157, 1123, 1045, 897, 881, 772, 743, 723 *cm^{-1}*

LRMS (EI) m/z 301 $(M)^+$

HRMS (EI) calcd for $(M)^+$ $C_{16}H_{17}NO_3S$: 303.0929, found: 303.0926.

Rf = 0.29 (hexane/AcOEt: 1/1)

2-(4-Methoxybenzyl)-*N*-(methylsulfonyl)benzamide (1k)

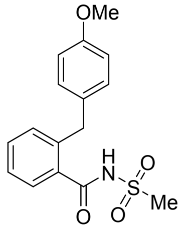

79% yield (518.7 mg, 1.62 mmol) from 2-(4-methoxylbenzyl)benzoic acid (500.0 mg, 2.06 mmol).

colorless solid, **mp** 105 °C (recrystallized from hexane/AcOEt)

^{1}H NMR (500 MHz, $CDCl_3$/TMS) δ 7.95 (brs, 1H), 7.53 – 7.44 (m, 2H), 7.35 – 7.29 (m, 2H), 7.03 (d, *J* = 8.8 Hz, 2H), 6.80 (d, *J* = 8.8 Hz, 2H), 4.20 (s, 2H), 3.76 (s, 3H), 3.15 (s, 3H).

^{13}C{^{1}H} NMR (126 MHz, $CDCl_3$/TMS) δ 167.4, 158.5, 141.3, 132.5, 132.4, 132.4, 132.1, 130.1, 127.9, 127.0, 114.2, 55.5, 41.5, 38.3.

IR (neat):3184, 3030, 2920, 2830, 1680, 1508, 1437, 1410, 1341, 1244, 1165, 1125, 1103, 1072, 1036, 980, 899, 854, 797, 773, 748, 737, 662 *cm^{-1}*

LRMS (EI) m/z 319 $(M)^+$

HRMS (EI) calcd for $(M)^+$ $C_{16}H_{17}NO_4S$: 319.0878, found: 319.0880.

Rf = 0.26 (hexane/AcOEt: 1/1)

2-(4-Fluorobenzyl)-*N*-(methylsulfonyl)benzamide (1l)

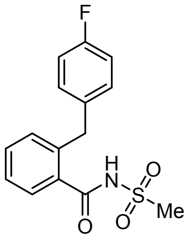

92% yield (737.6 mg, 2.4 mmol) from 2-(4-fluorobenzyl)benzoic acid (600.0 mg, 2.61 mmol).

colorless solid, **mp** 114 – 115 °C (recrystallized from hexane/AcOEt)

^{1}H NMR (500 MHz, $CDCl_3$/TMS) δ 8.19 (brs, 1H), 7.54 – 7.44 (m, 2H), 7.36 – 7.27 (m, 2H), 7.14 – 7.05 (m, 2H), 6.99 – 6.90 (m, 2H), 4.23 (s, 2H), 3.16 (s, 3H).

^{13}C{^{1}H} NMR (126 MHz, $CDCl_3$/TMS) δ 161.6 (d, *J* = 245.0 Hz), 160.6, 140.9, 136.0 (d, *J* = 3.5 Hz), 132.4, 132.1, 132.0, 130.5 (d, *J* = 7.8 Hz), 127.9, 127.0, 115.4 (d, *J* = 21.4 Hz), 41.4, 38.2.

^{19}F NMR (471 MHz, $CDCl_3$) δ –115.8.

IR (neat):3179, 3011, 2930, 1678, 1506, 1437, 1337, 1225, 1163, 1065, 976, 849, 779, 662 *cm^{-1}*

LRMS (EI) m/z 307 $(M)^+$
HRMS (EI) calcd for $(M)^+$ $C_{15}H_{14}FNO_3S$: 307.0678, found: 307.0678.
Rf = 0.31 (hexane/AcOEt: 1/1)

N-(Methylsulfonyl)-2-(naphthalen-1-ylmethyl)benzamide (1m)

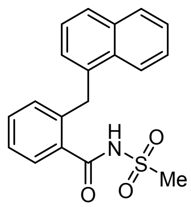

87% yield (1.09 g, 3.21 mmol) from 2-(naphthalen-1-ylmethyl)benzoic acid (0.96 g, 3.67 mmol).
colorless solid, **mp** 158 °C (recrystallized from hexane/AcOEt)
^{1}H NMR (400 MHz, $CDCl_3$/TMS) δ 8.08 – 8.00 (m, 1H), 7.89 – 7.81 (m, 1H), 7.75 (d, *J* = 8.2 Hz, 1H), 7.57 – 7.46 (m, 3H), 7.43 (td, *J* = 7.6, 1.5 Hz, 1H), 7.40 – 7.30 (m, 2H), 7.23 (dd, *J* = 7.6, 0.7 Hz, 1H), 7.04 (dd, *J* = 7.1, 1.1 Hz, 1H), 4.73 (s, 2H), 2.87 (s, 3H).
^{13}C{^{1}H} NMR (101 MHz, $CDCl_3$/TMS) δ 167.4, 140.6, 136.5, 134.0, 132.6, 132.4, 132.2, 131.9, 128.8, 127.8, 127.5, 127.0 (2C), 126.6, 126.1, 125.6, 124.0, 41.1, 35.8.
IR (neat): 3211, 3044, 1680, 1495, 1445, 1398, 1325, 1248, 1234, 1161, 1117, 1063, 974, 959, 893, 847, 791, 773 cm^{-1}
LRMS (EI) m/z 339 $(M)^+$
HRMS (EI) calcd for $(M)^+$ $C_{19}H_{17}NO_3S$:339.0929, found: 339.0924.
Rf = 0.24 (hexane/AcOEt: 1/1)

N-(Methylsulfonyl)-2-(thiophen-2-ylmethyl)benzamide (1n)

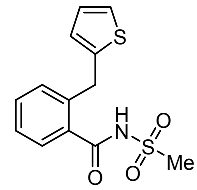

93% yield (1.18 g, 4.01 mmol) from 2-(thiophen-2-ylmethyl)benzoic acid (1.0 g, 4.33 mmol).
colorless solid, **mp** 98 – 99 °C (recrystallized from hexane/AcOEt)
^{1}H NMR (500 MHz, $CDCl_3$/TMS) δ 8.08 (brs, 1H), 7.56 – 7.46 (m, 2H), 7.43 – 7.32 (m, 2H), 7.14 (dd, *J* = 5.2, 1.2 Hz, 1H), 6.90 (dd, *J* = 5.2, 3.4 Hz, 1H), 6.77 – 6.72 (m, 1H), 4.45 (s, 2H), 3.20 (s, 3H).
^{13}C{^{1}H} NMR (126 MHz, $CDCl_3$/TMS) δ 167.2, 143.4, 140.4, 132.6, 132.2, 131.8, 128.0, 127.5, 127.0, 125.9, 124.5, 41.5, 33.7.
IR (neat):33188, 3055, 3032, 2932, 2916, 1670, 1497, 1447, 1404, 1327, 1248, 1161, 1134, 1061, 978, 883, 845, 770, 712, 689 cm^{-1}
LRMS (EI) m/z 295 $(M)^+$
HRMS (EI) calcd for $(M)^+$ $C_{13}H_{13}NO_3S_2$:295.0337, found: 295.0333.
Rf = 0.15 (hexane/AcOEt: 1/1)

2-[(1-Methyl-1*H*-indol-3-yl)methyl]-*N*-(methylsulfonyl)benzamide (1o)

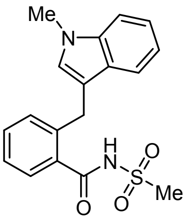

61% yield (300.3 mg, 0.877 mmol) from 2-[(1-methyl-1*H*-indol-3-yl)methyl]benzoic acid (381.7 mg, 1.44 mmol).
colorless solid, **mp** 147 °C (recrystallized from hexane/AcOEt)

^{1}**H NMR** (400 MHz, $CDCl_3$/TMS) δ 7.98 (brs, 1H), 7.51 – 7.42 (m, 4H), 7.34 – 7.26 (m, 2H), 7.20 (ddd, *J* = 8.2, 6.9, 1.1 Hz, 1H), 7.06 (ddd, *J* = 8.0, 6.9, 1.1 Hz, 1H), 6.77 (s, 1H), 4.34 (s, 2H), 3.72 (s, 3H), 3.05 (s, 3H).

^{13}**C{^{1}H} NMR** (101 MHz, $CDCl_3$/TMS) δ 167.7, 141.2, 137.3, 132.8, 132.1, 131.6, 128.2, 128.0, 127.7, 126.8, 122.1, 119.3, 119.2, 113.3, 109.4, 41.3, 32.8, 29.6.

IR(neat): 3204, 3055, 3028, 3005, 2928, 1674, 1435 ,1406, 1339, 1339, 1238, 1163, 1113, 1076, 1069, 984, 961, 893, 835, 733, 714, 669 *cm^{-1}*

LRMS (EI) m/z 342 $(M)^+$

HRMS (EI) calcd for $(M)^+$ $C_{18}H_{18}N_2O_3S$: 342.1038, found: 342.1040.

Rf = 0.14 (hexane/AcOEt: 1/1)

2-[(1-Acetyl-1*H*-indol-3-yl)methyl]-*N*-(methylsulfonyl)benzamide (1p)

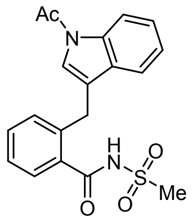

73% yield (322.3 mg, 0.87 mmol) from 2-[(1-acetyl-1*H*-indol-3-yl)methyl]benzoic acid (349.3 mg, 1.19 mmol).

colorless solid, **mp** 188 °C (recrystallized from hexane/AcOEt)

^{1}**H NMR** (500 MHz, $CDCl_3$/TMS) δ 8.35 (d, *J* = 8.3 Hz, 1H), 8.27 (brs, 1H), 7.51 – 7.45 (m, 3H), 7.43 (d, *J* = 7.8 Hz, 1H), 7.36 – 7.27 (m, 2H), 7.24 – 7.16 (m, 2H), 4.32 (s, 2H), 3.19 (s, 3H), 2.55 (s, 3H).

^{13}**C{^{1}H} NMR** (126 MHz, $CDCl_3$/TMS) δ 168.9, 167.5, 140.0, 136.1, 132.5, 131.8 (2C), 130.4, 127.8, 127.2, 125.5, 124.1, 123.7, 120.9, 119.3, 116.9, 41.6, 29.2, 24.1.

IR(neat): 3227, 3119, 3026, 2930, 2851, 1695, 1674, 1601, 1450, 1437, 1387, 1373, 1329, 1242, 1223, 1159, 1117, 1061, 1016, 968, 935, 893, 849, 781, 745, 667, 654, 638 *cm^{-1}*

LRMS (EI) m/z 370 $(M)^+$

HRMS (EI) calcd for $(M)^+$ $C_{19}H_{18}N_2O_4S$: 370.0987, found: 370.0987.

Rf = 0.15 (hexane/AcOEt: 1/2)

***N*-(Methylsulfonyl)-2-[(1-tosyl-1*H*-indol-3-yl)methyl]benzamide (1q)**

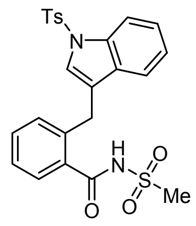

71% yield (696.9 mg, 1.44 mmol) from 2-[(1-tosyl-1*H*-indol-3-yl)methyl]benzoic acid (818.9 mg, 2.02 mmol).

colorless solid, **mp** 202 °C (recrystallized from hexane/AcOEt)

^{1}**H NMR** (500 MHz, $CDCl_3$/TMS) δ 8.12 (brs, 1H), 7.93(d, *J* = 8.3 Hz, 1H), 7.71 (d, *J* = 8.4 Hz, 2H), 7.55 – 7.50 (m, 1H), 7.49– 7.41 (m, 2H), 7.39 – 7.27 (m, 3H), 7.24 – 7.15 (m, 4H), 4.30 (s, 2H), 3.02 (s, 3H), 2.34 (s, 3H).

^{13}**C{^{1}H} NMR** (101 MHz, $CDCl_3$/TMS) δ 167.2, 145.2, 139.9, 135.4, 132.6, 132.1, 131.7, 130.7, 130.1, 128.1, 127.4, 127.0, 125.1 (2C), 124.5, 123.5, 122.0, 120.0, 113.8, 41.4, 29.0, 21.7.

IR (neat): 3229, 3028, 2928, 1697, 1447, 1429, 1398, 1362, 1339, 1242, 1209, 1161, 1119, 1094, 1059, 1018, 970, 893, 849, 814, 781, 743 *cm^{-1}*

LRMS (EI) m/z 482 $(M)^+$

HRMS (EI) calcd for $(M)^+$ $C_{24}H_{22}N_2O_5S_2$: 482.0970, found: 482.0969.

Rf = 0.27 (hexane/AcOEt: 1/2)

2-Ethyl-*N*-(methylsulfonyl)benzamide (1r)

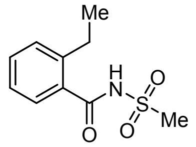

53% yield (783.0 mg, 3.44 mmol) from 2-ethyl benzoic acid (1.0 g, 6.66 mmol).

colorless solid, **mp** 96 °C (recrystallized from hexane/AcOEt)

^{1}H NMR (500 MHz, $CDCl_3$/TMS) δ 8.40 (brs, 1H), 7.49 – 7.40 (m, 2H), 7.31 (d, *J* = 7.7 Hz, 1H), 7.28 – 7.22 (m, 1H), 3.39 (s, 3H), 2.84 (q, *J* = 7.5 Hz, 2H), 1.24 (t, *J* = 7.5 Hz, 3H).

^{13}C{^{1}H} NMR (126 MHz, $CDCl_3$/TMS) δ 167.7, 144.5, 132.3, 131.8, 130.5, 127.5, 126.2, 41.8, 26.7, 15.9.

IR (neat): 3117, 2984, 2955, 2930, 2868, 1680, 1493, 1456, 1437, 1410, 1327, 1258, 1229, 1134, 1074 1057, 976, 968, 889, 847, 793, 764, 660 *cm^{-1}*

LRMS (EI) m/z 227 $(M)^+$

HRMS (EI) calcd for $(M)^+$ $C_{10}H_{13}NO_3S$: 227.0616, found: 227.0610.

Rf = 0.34 (hexane/AcOEt: 1/1)

Synthesis of 2-(Naphthalen-1-ylmethyl)benzoic Acid (S1)

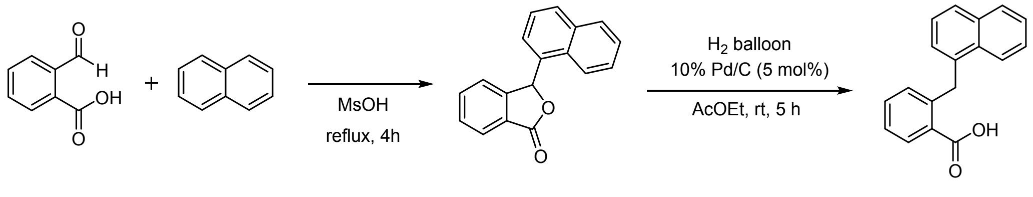

(Step 1) A mixture of phthaladehydic acid (1.5g, 10 mmol) and naphthalene (2.56 g, 20 mmol) in MsOH (10 mL) was stirred at room temperature. After 20 h, the mixture was poured into ice water (150 mL), and the solid obtained was collected by filtration and washed with H_2O and cold diethyl ether. The solid was dried (2.5g, 9.6 mmol, 96%) and submitted to the next reaction without further purification.

(Step 2) In a flask, the above obtained phthalide (1 g, 3.84 mmol) and 10% Pd/C (204.3 mg, 0.192 mmol, 5 mol%) were dissolved with AcOEt (30 mL). The flask was evacuated and refilled with H_2 using a balloon. After stirring for 5 h, the mixture was filtered by Celite®, washed with AcOEt, and evaporated *in vacuo*. The crude was purified by silica gel column chromatography eluting with hexane/AcOEt (4:1) to afford the desired benzoic acid (**S1**, 60 % yield, 605.2 mg, 2.31 mmol).

colorless solid, **mp** 148 – 149 °C (recrystallized from hexane/AcOEt)

^{1}H NMR (400 MHz, $CDCl_3$/TMS) δ 8.14 – 8.08 (m, 1H), 7.94 – 7.87 (m, 1H), 7.86 – 7.80 (m, 1H), 7.74 (d, *J* = 8.2 Hz, 1H), 7.46 – 7.34 (m, 3H), 7.34 – 7.23 (m, 2H), 7.14 (d, *J* = 7.0 Hz, 1H), 6.95 – 6.89 (m, 1H), 4.89 (s, 2H).

^{13}C{^{1}H} NMR (101 MHz, $CDCl_3$/TMS) δ 173.4, 143.4, 136.6, 134.1, 133.2, 132.5, 131.8, 131.1, 128.8, 128.6, 127.5, 127.3, 126.4, 126.2, 125.7 (2C), 124.4, 37.0.

IR (neat): 3061, 3005, 2864, 2810, 1672, 1570, 1487, 1312, 1290, 1254, 1148, 1082, 937, 777, 763, 737 *cm^{-1}*

LRMS (EI) m/z 262 $(M)^+$

HRMS (EI) calcd for $(M)^+$ $C_{18}H_{14}O_2$: 262.0994, found: 262.0992.

Rf = 0.16 (hexane/AcOEt: 4/1)

Representative Procedure for Synthesis of Benzoic Acid (S2 and S3)

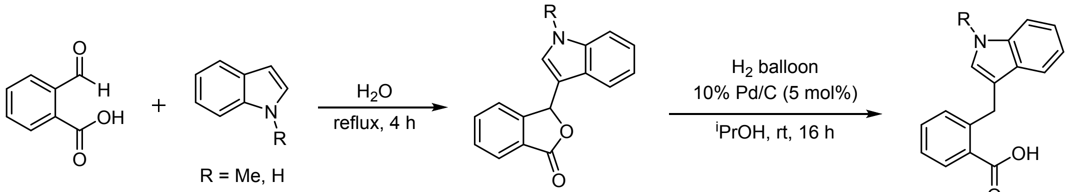

R = Me, **S2**
H, **S3**

(Step 1) A mixture of phthalaldehydic acid (1.0 g, 6.66 mmol) and *N*-methyl indole (873.6 mg, 6.66 mmol) in water (30 mL) was reflux for 4 h. Upon cooling to rt, the solid obtained was filtered and washed with a small amount of cold EtOH. The product was obtained in quantitative yield (99% yield, 1.72 g, 6.63 mmol) and submitted to the next reaction without further purification.

(Step 2) In a flask, the above obtained phthalide (1.0 g, 3.80 mmol) and 10% Pd/C (202.1 mg, 0.19 mmol, 5 mol%) were dissolved with ^{i}PrOH. (30 mL). The flask was evacuated and refilled with H_2 using a balloon. After stirring for 16 h, the mixture was filtered by Celite®, washed with AcOEt, and evaporated *in vacuo*. The crude was purified by silica gel column chromatography eluting with hexane/AcOEt to afford 2-[(1-methyl-1*H*-indol-3-yl)methyl]benzoic acid **S2** (63% yield, 635.7 mg, 2.4 mmol).

2-[(1-Methyl-1*H*-indol-3-yl)methyl]benzoic Acid (S2)

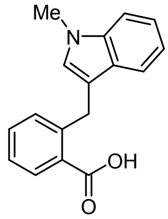

63 % yield (635.7 mg, 2.4 mmol)

colorless solid, **mp** 188 – 189 °C (recrystallized from hexane/AcOEt)

^{1}H NMR (400 MHz, $CDCl_3$/TMS) δ 8.04 (dd, *J* = 7.9, 1.5 Hz, 1H), 7.54 (dt, *J* = 7.9, 1.0 Hz, 1H), 7.45 – 7.38 (m, 1H), 7.36 – 7.26 (m, 3H), 7.21 (ddd, *J* = 8.2, 6.9, 1.2 Hz, 1H), 7.07 (ddd, *J* = 8.0, 6.9, 1.1 Hz, 1H), 6.74 (s, 1H), 4.55 (s, 2H), 3.71 (s, 3H).

^{13}C{^{1}H} NMR (101 MHz, $CDCl_3$/TMS) δ 172.4, 144.2, 137.3, 133.0, 131.6, 131.2, 128.4, 128.2, 127.7, 126.2, 121.7, 119.4, 119.0, 113.9, 109.3, 32.7, 29.6.

IR (neat): 3080, 3055, 2907, 2822, 2650, 1682, 1574, 1485, 1452, 1410, 1373, 1310, 1273, 1252, 1219, 1146, 1123, 1061, 1009, 910, 870, 826, 781, 760, 729, 698, 664 *cm^{-1}*

LRMS (EI) m/z 265 $(M)^+$

HRMS (EI) calcd for $(M)^+$ $C_{17}H_{15}NO_2$: 265.1103, found: 265.1103.

Rf = 0.18 (hexane/AcOEt: 1/1)

2-[(1*H*-Indol-3-yl)methyl]benzoic Acid (S3)

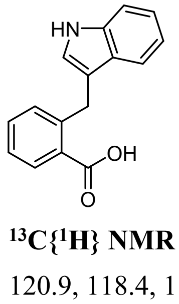

86% yield (1.08 g, 4.3 mmol)

pale brown solid, **mp** 210 – 211 °C (recrystallized from hexane/AcOEt)

^{1}H NMR (400 MHz, DMSO-*d_6*) δ 10.77 (brs, 1H), 7.76 (dd, *J* = 7.5, 1.4 Hz, 1H), 7.45 – 7.36 (m, 2H), 7.35 – 7.29 (m, 2H), 7.26 (td, *J* = 7.5, 1.4 Hz, 1H), 7.04 (ddd, *J* = 8.2, 7.0, 1.2 Hz, 1H), 6.99 (d, *J* = 2.4 Hz, 1H), 6.92 (ddd, *J* = 8.0, 7.0, 1.0 Hz, 1H), 4.40 (s, 2H).

^{13}C{^{1}H} NMR (101 MHz, DMSO-*d_6*) δ 169.2, 142.2, 136.3, 131.3, 130.9, 130.5, 129.8, 127.1, 125.8, 123.2, 120.9, 118.4, 118.3, 113.7, 111.3, 28.6.

IR (neat): 3401, 3059, 2872, 1682, 1570, 1485, 1456, 1404, 1288, 1250, 1144, 1094, 922, 824, 779, 738, 731 *cm⁻¹*

LRMS (EI) m/z 251(M)$^+$

HRMS (EI) calcd for (M)$^+$ $C_{16}H_{13}NO_2$: 251.0946, found: 251.0944.

Rf = 0.43 (hexane/AcOEt: 1/1)

Representative Procedure for Synthesis of Benzoic Acid (S4 and S5)

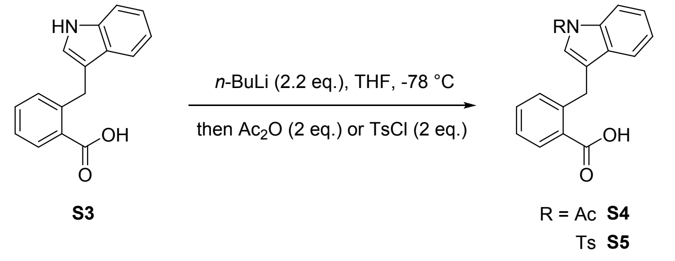

In a dry flask, the solution of 2-[(1*H*-indol-3-yl)methyl]benzoic acid **S3** (753.9 mg, 3.0 mmol) in THF (15 mL) was cooled to –78°C. 1.6 M *n*-BuLi (4.2 mL, 6.6 mmol) was slowly added with stirring at –78°C. After stirring for 1 h, acetic anhydride (0.57 mL, 6.0 mmol) or TsCl (1.14 g, 6.0 mmol) was added and the reaction was warmed to rt, and stirred for 16 h. The reaction was quenched with 1 M HCl aq. and taken up in AcOEt. The organic layer was separated and the aqueous layer was extracted with AcOEt (twice). The collected organic layer was washed with brine, dried over $MgSO_4$, filtered, and concentrated *in vacuo*. The residue was purified by silica gel flash column chromatography eluting with hexane/AcOEt to afford the desired product.

2-(1-Acetyl-1*H*-indol-3-ylmethyl)benzoic Acid (S4)

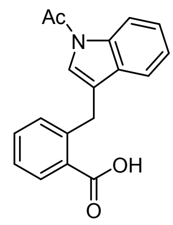

71% yield (626.1 mg, 0.185 mmol) from 2-[(1*H*-indol-3-yl)methyl]benzoic acid (753.9 mg, 3.0 mmol).

colorless solid, **mp** 248 – 249 °C (recrystallized from hexane/AcOEt)

^{1}**H NMR** (400 MHz, Acetone-d_6/TMS) δ 8.38 (d, *J* = 8.2 Hz, 1H), 7.97 (dd, *J* = 7.8, 1.1 Hz, 1H), 7.53 (ddd, *J* = 7.7, 1.3, 0.7 Hz, 1H), 7.49 – 7.38 (m, 3H), 7.37 – 7.27 (m, 2H), 7.25 – 7.18 (m, 1H), 4.52 (s, 2H), 2.59 (s, 3H).

^{13}**C{^{1}H} NMR** (101 MHz, Acetone-d_6/TMS) δ 169.4, 169.2, 142.2, 137.0, 132.8, 131.8, 131.7, 131.2, 127.3 (2C), 125.6, 125.1, 124.0, 122.3, 120.2, 117.1, 29.6, 23.96.

IR (neat): 3067, 3042, 3011, 1701, 1684, 1603, 1449, 1379, 1329, 1304, 1271, 1238, 1207, 1144, 1117, 1003, 972, 932, 804, 746, 712 *cm⁻¹*

LRMS (EI) m/z 293 (M)$^+$

HRMS (EI) calcd for (M)$^+$ $C_{18}H_{15}NO_3$: 293.1052, found: 293.1054.

Rf = 0.14 ($CHCl_3$/MeOH: 100/1)

2-[(1-Tosyl-1*H*-indol-3-yl)methyl]benzoic acid (S5)

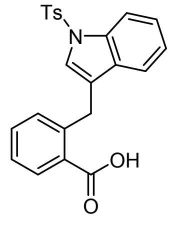

82% yield (992.6 mg, 2.45 mmol) from 2-[(1*H*-indol-3-yl)methyl]benzoic acid (753.9 mg, 3.0 mmol).

colorless solid, **mp** 219 – 220 °C (recrystallized from hexane/AcOEt)

^{1}H NMR (400 MHz, Acetone-d_6/TMS) δ 8.03 – 7.92 (m, 2H), 7.81 – 7.74 (m, 2H), 7.56 – 7.51 (m, 1H), 7.50 – 7.44 (m, 1H), 7.40 – 7.27 (m, 6H), 7.20 (ddd, *J* = 7.9, 7.2, 1.0 Hz, 1H), 4.49 (s, 2H), 2.33 (s, 3H).
^{13}C{^{1}H} NMR (101 MHz, Acetone-d_6/TMS) δ 169.0, 146.1, 141.8, 136.2, 135.9, 132.9, 132.0, 131.9, 131.8, 131.1, 130.9, 127.7, 127.5, 125.5, 125.3, 124.1, 123.9, 120.9, 114.5, 29.7, 21.4.
IR (neat): 3105, 2970, 2872, 2814, 1684, 1670, 1601, 1447, 1358, 1308, 1281, 1269, 1256, 1207, 1169, 1148, 1113, 1096, 1074, 1053, 976, 949, 930, 818, 781, 733, 706, 687, 667, 604 *cm^{-1}*
LRMS (EI) m/z 406 $(M)^+$
HRMS (EI) calcd for $(M)^+$ $C_{23}H_{19}NO_4S$: 405.1035, found: 406.1034.
Rf = 0.12 (hexane/AcOEt: 1/1)

Representative Procedure for Synthesis of Isoindolinone (2a-q)
In a test tube, 2-benzyl-*N*-(methylsulfonyl)benzamide **1f** (72.3 mg, 0.25 mmol), 10% Pd/C (26.6 mg, 0.025 mmol), and Na_2HPO_4 (7.1 mg, 0.05 mmol) were added. The tube was evacuated and backfilled with Ar three times and then *p*-xylene (5 mL) was added. The mixture was degassed with Ar bubbling for 5 min (in cases of **1f**,**j**–**r**). The tube was sealed and heated at 150 °C in an oil bath for 24 h. After cooling to room temperature, the solution was submitted to silica gel column chromatography eluting with hexane/AcOEt (3:1) to afford 2-(methylsulfonyl)-3-phenylisoindolin-1-one **2f** (86% yield, 61.6 mg, 0.214 mmol) as a colorless solid.

3-Phenyl-2-(*p*-tolylsulfonyl)isoindolin-1-one (2a)

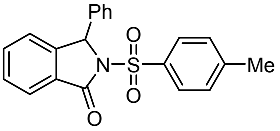

42% yield (38.0 mg, 0.105 mmol)
colorless solid, **mp** 173 – 174 ℃ (recrystallized from hexane/AcOEt, lit.[6)] mp 173 – 174 ºC).
^{1}H NMR (400 MHz, $CDCl_3$/TMS) δ 7.86 (d, *J* = 7.6 Hz, 1H), 7.59 – 7.50 (m, 3H), 7.46 (t, *J* = 7.5 Hz, 1H), 7.35 – 7.21 (m, 3H), 7.19 – 7.10 (m, 3H), 7.10 – 7.04 (m, 2H), 6.21 (s, 1H), 2.36 (s, 3H).
^{13}C{^{1}H} NMR (101 MHz, $CDCl_3$/TMS) δ 166.6, 146.6, 144.8, 137.2, 136.2, 134.4, 129.3, 129.13, 129.10, 128.9, 128.8, 128.3, 128.2, 124.9, 123.9, 65.8, 21.7.
LRMS (FAB) *m/z*: 364 (M^++1).
HRMS: Calcd. for $C_{21}H_{18}NO_3S$: 364.1007, found: 364.1006.
IR (neat): 3065, 3026, 1722, 1597, 1458, 1364, 1285, 1184, 1167, 1103, 842, 806, 789, 756, 735 *cm^{-1}*
Rf = 0.29 (hexane/AcOEt: 4/1)

3-Phenyl-2-(*o*-tolylsulfonyl)isoindolin-1-one (2b)

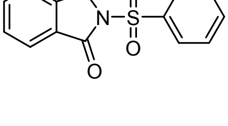

24% yield (21.0 mg, 0.0578 mmol)
colorless solid, **mp** 202 – 203 ℃ (recrystallized from hexane/AcOEt)
^{1}H NMR (500 MHz, $CDCl_3$/TMS) δ 7.88 (dd, *J* = 8.1, 1.4 Hz, 1H), 7.85 (d, *J* = 7.6 Hz, 1H), 7.57 (td, *J* = 7.5, 1.2 Hz, 1H), 7.47 (t, *J* = 7.5 Hz, 1H), 7.41 (td, *J* = 7.5, 1.4 Hz, 1H), 7.31 – 7.26 (m, 3H), 7.24 – 7.15 (m, 5H), 6.27 (s, 1H), 2.51 (s, 3H).
^{13}C{^{1}H} NMR (126 MHz, $CDCl_3$/TMS) δ 166.8, 146.7, 138.7, 137.5, 137.4, 134.5, 133.8, 132.5, 131.3, 129.2, 129.1, 128.9, 128.8, 127.8, 126.3, 125.0, 123.9, 66.2, 20.4.
IR (neat): 3063, 3030, 2974, 2934, 1734, 1558, 1506, 1456, 1350, 1288, 1169, 1099, 758, 739, 694 *cm^{-1}*

LRMS (EI) m/z 363 $(M)^+$

HRMS (EI) calcd for $(M)^+$ $C_{21}H_{17}NO_3S$: 363.0929, found: 363.0926.

Rf = 0.34 (hexane/AcOEt: 3/1)

3-Phenyl-2-(phenylsulfonyl)isoindolin-1-one (2c)

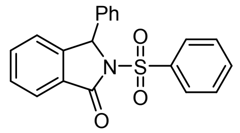

33% yield (29.2 mg, 0.0836 mmol)

colorless solid, **mp** 176 – 177 °C (recrystallized from hexane/AcOEt)

^{1}H NMR (500 MHz, $CDCl_3$/TMS) δ 7.88 (d, *J* = 7.6 Hz, 1H), 7.67 – 7.61 (m, 2H), 7.56 (td, *J* = 7.5, 1.2 Hz, 1H), 7.53 – 7.45 (m, 2H), 7.36 – 7.28 (m, 3H), 7.26 – 7.21 (m, 2H), 7.16 (dd, *J* = 7.7, 0.9 Hz, 1H), 7.08 – 7.03 (m, 2H), 6.23 (s, 1H).

^{13}C{^{1}H} NMR (126 MHz, $CDCl_3$/TMS) δ 166.5, 146.6, 139.2, 137.0, 134.5, 133.7, 129.2, 129.0, 128.9, 128.8, 128.7, 128.2, 128.1, 124.9, 123.9, 65.8.

IR (neat): 3065, 3030, 2916, 2849, 1732, 1611, 1466, 1449, 1364, 1288, 1171, 1101, 1090, 723, 687 *cm^{-1}*

LRMS (EI) m/z 285 $(M)^+$

HRMS (EI) calcd for $(M)^+$ $C_{20}H_{15}NO_3S$: 349.0773, found: 349.0771.

Rf = 0.29 (hexane/AcOEt: 3/1)

2-[(4-Methoxyphenyl)sulfonyl]-3-phenylisoindolin-1-one (2d)

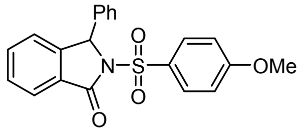

6% NMR yield

colorless solid, **mp** 176 °C (recrystallized from hexane/AcOEt)

^{1}H NMR (500 MHz, $CDCl_3$/TMS) δ 7.86 (d, *J* = 7.6 Hz, 1H), 7.60 – 7.52 (m, 3H), 7.47 (t, *J* = 7.5 Hz, 1H), 7.34 –7.24 (m, 3H), 7.16 (dd, *J* = 7.7, 0.9 Hz, 1H), 7.11 – 7.06 (m, 2H), 6.82 – 6.76 (m, 2H), 6.21 (s, 1H), 3.82 (s, 3H).

^{13}C{^{1}H} NMR (126 MHz, $CDCl_3$/TMS) δ 166.6, 163.9, 146.6, 137.2, 134.4, 130.7, 130.6, 129.2, 129.1, 128.9, 128.8, 128.2, 124.8, 123.9, 113.9, 65.8, 55.7.

IR (neat): 3063, 3009, 2943, 2843, 1730, 1595, 1578, 1497, 1466, 1456, 1364, 1288, 1261, 1180, 1165, 1092, 1028, 831, 758, 739, 692, 667 *cm^{-1}*

LRMS (EI) m/z 315 $(M)^+$

HRMS (EI) calcd for $(M)^+$ $C_{21}H_{17}NO_4S$: 379.0878, found: 379.0878.

Rf = 0.24 (hexane/AcOEt: 3/1)

2-(Methylsulfonyl)-3-phenylisoindolin-1-one (2f)

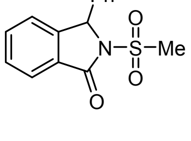

86% yield (61.6 mg, 0.214 mmol) from **1f** (72.3 mg, 0.25 mmol)

92% yield (265.0 mg, 0.922 mmol) from **1f** (753.9 mg, 1.0 mmol)

colorless solid, **mp** 200 °C (recrystallized from hexane/AcOEt)

^{1}H NMR (500 MHz, $CDCl_3$/TMS) δ 7.96 (d, *J* = 7.6 Hz, 1H), 7.61 (td, *J* = 7.5, 1.2 Hz, 1H), 7.53 (t, *J* = 7.5 Hz, 1H), 7.39 – 7.31 (m, 3H), 7.24 – 7.17 (m, 3H), 6.16 (s, 1H), 3.11 (s, 3H).

^{13}C{^{1}H} NMR (126 MHz, $CDCl_3$/TMS) δ 167.5, 146.7, 137.3, 134.8, 129.3, 129.2, 129.1, 128.7, 127.5, 125.1, 124.0, 65.3, 42.3.

IR (neat): 3030, 3011, 2930, 1732, 1558, 1541, 1506, 1456, 1354, 1290, 1165, 1103, 968, 827, 754, 739 *cm^{-1}*

LRMS (EI) m/z 287 $(M)^+$

HRMS (EI) calcd for $(M)^+$ $C_{15}H_{13}NO_3S$: 287.0616, found: 287.0620.

Rf = 0.11 (hexane/AcOEt: 3/1)

3-Phenyl-2-[(trifluoromethyl)sulfonyl]isoindolin-1-one (2g)

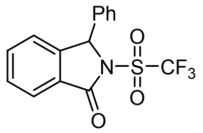

46% yield (39.2 mg, 0.115 mmol)

colorless solid, **mp** 141 – 142 ℃ (recrystallized from hexane/AcOEt)

^{1}H NMR (500 MHz, $CDCl_3$/TMS) δ 8.00 (d, *J* = 7.6 Hz, 1H), 7.67 (td, *J* = 7.6, 1.2 Hz, 1H), 7.58 (t, *J* = 7.6 Hz, 1H), 7.38 – 7.32 (m, 3H), 7.23 – 7.16 (m, 3H), 6.20 (s, 1H).

^{13}C{^{1}H} NMR (126 MHz, $CDCl_3$/TMS) δ 165.6, 146.8, 136.2, 135.8, 129.8, 129.6, 129.2, 128.1, 127.2, 125.8, 124.3, 119.32 (q, *J* = 323.5 Hz), 66.9.

^{19}F NMR (376 MHz, $CDCl_3$) δ –74.5.

IR (neat): 3065, 3032, 1761, 1558, 1506, 1456, 1406, 1283, 1209, 1140, 1070, 849, 735, 689, 637 *cm^{-1}*

LRMS (EI) m/z 341 $(M)^+$

HRMS (EI) calcd for $(M)^+$ $C_{15}H_{10}F_3NO_3S$: 341.0333, found: 341.0331.

Rf = 0.32 (hexane/AcOEt: 3/1)

2-(Methylsulfonyl)-3-(*p*-tolyl)isoindolin-1-one (2j)

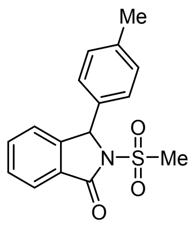

91% yield (68.6 mg, 0.228 mmol)

colorless solid, **mp** 205 ℃ (recrystallized from hexane/AcOEt)

^{1}H NMR (400 MHz, $CDCl_3$/TMS) δ 7.95 (dt, *J* = 7.6, 1.1 Hz, 1H), 7.59 (dd, *J* = 7.6, 1.3 Hz, 1H), 7.52 (t, *J* = 7.4 Hz, 1H), 7.22 – 7.13 (m, 3H), 7.13 – 7.06 (m, 2H), 6.13 (s, 1H), 3.10 (s, 3H), 2.33 (s, 3H).

^{13}C{^{1}H} NMR (101 MHz, $CDCl_3$/TMS) δ 167.5, 146.9, 139.0, 134.7, 134.2, 129.9, 129.2, 128.7, 127.5, 125.0, 124.0, 65.2, 42.3, 21.3.

IR (neat): 3013, 2930, 1732, 1344, 1287, 1159, 1099, 1088, 962, 849, 839, 793, 756, 741, 719, 685 *cm^{-1}*

LRMS (EI) m/z 301 $(M)^+$

HRMS (EI) calcd for $(M)^+$ $C_{16}H_{15}NO_3S$: 301.0773, found: 301.0776.

Rf = 0.18 (hexane/AcOEt: 3/1)

3-(4-Methoxyphenyl)-2-(methylsulfonyl)isoindolin-1-one (2k)

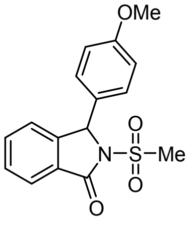

92% yield (73.1 mg, 0.230 mmol)

colorless solid, **mp** 141 – 142 ℃ (recrystallized from hexane/AcOEt)

^{1}H NMR (400 MHz, $CDCl_3$/TMS) δ 7.96 (d, *J* = 7.6 Hz, 1H), 7.61 (td, *J* = 7.6, 1.2 Hz, 1H), 7.53 (t, *J* = 7.6 Hz, 1H), 7.20 (dd, *J* = 7.7, 0.9 Hz, 1H), 7.13 (d, *J* = 8.7 Hz, 2H), 6.87 (d, *J* = 8.7 Hz, 2H), 6.13 (s, 1H), 3.80 (s, 3H), 3.07 (s, 3H).

^{13}C{^{1}H} NMR (101 MHz, $CDCl_3$/TMS) δ 167.3, 160.1, 146.9, 134.7, 129.2, 129.0, 128.9, 128.7, 124.9, 123.9, 114.5, 65.0, 55.4, 42.3.

IR (neat): 3011, 2930, 2837, 1719, 1512, 1337, 1288, 1244, 1159, 1098, 1036, 968, 849, 754, 723, 685 *cm^{-1}*

LRMS (EI) m/z 317 $(M)^+$

HRMS (EI) calcd for $(M)^+$ $C_{16}H_{15}NO_4S$: 317.0722, found: 317.0719.

Rf = 0.09 (hexane/AcOEt: 3/1)

3-(4-Fluorophenyl)-2-(methylsulfonyl)isoindolin-1-one (2l)

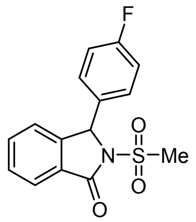

44% yield (25.1 mg, 0.111 mmol)

colorless solid, **mp** 134 ℃ (recrystallized from hexane/AcOEt)

^{1}H NMR (500 MHz, $CDCl_3$/TMS) δ 7.96 (d, *J* = 7.7 Hz, 1H), 7.63 (td, *J* = 7.6, 1.2 Hz, 1H), 7.55 (t, *J* = 7.5 Hz, 1H), 7.24 – 7.16 (m, 3H), 7.08 – 7.01 (m, 2H), 6.15 (s, 1H), 3.14 (s, 3H).

$^{13}C\{^1H\}$ NMR (126 MHz, $CDCl_3$/TMS) δ 167.4, 163.04 (d, *J* = 248.7 Hz), 146.5, 134.9,133.2 (d, *J* = 3.5 Hz), 129.51, 129.5 (d, *J* = 8.4 Hz), 128.7, 125.2, 124.0, 116.29 (d, *J* = 21.6 Hz), 64.6, 42.4.

^{19}F NMR (471 MHz, $CDCl_3$) δ –111.6.

IR (neat): 3032, 3017, 2934, 1732, 1603, 1508, 1337, 1287, 1229, 1159, 1098, 964, 853, 756, 739, 719, 685 *cm^{-1}*

LRMS (EI) m/z 305 $(M)^+$

HRMS (EI) calcd for $(M)^+$ $C_{15}H_{12}FNO_3S$: 305.0522, found: 305.0516.

Rf = 0.15 (hexane/AcOEt: 3/1)

2-(Methylsulfonyl)-3-(naphthalene-1-yl)isoindolin-1-one (2m)

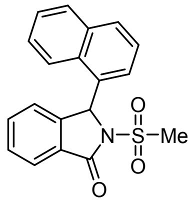

82% yield (69.4 mg, 0.206 mmol)

colorless solid, **mp** 193 – 194 ℃ (recrystallized from hexane/AcOEt)

^{1}H NMR (400 MHz, $CDCl_3$/TMS) δ 8.00 (d, *J* = 7.5 Hz, 1H), 7.92 – 7.76 (m, 4H), 7.63 – 7.47 (m, 4H), 7.21 (dq, *J* = 7.9, 1.0 Hz, 1H), 7.08 (dd, *J* = 8.5, 1.9 Hz, 1H), 6.33 (s, 1H), 3.11 (s, 3H).

$^{13}C\{^1H\}$ NMR (101 MHz, $CDCl_3$/TMS) δ 167.6, 146.7, 134.8, 134.3, 133.6, 133.4, 129.44, 129.39, 128.8, 128.3, 128.00, 127.96, 126.98, 126.95, 125.2, 124.1, 123.6, 65.6, 42.4.

IR (neat):3046, 3011, 2926, 2772, 1722, 1464, 1348, 1165, 1099, 1084, 968, 750, 733, 692, 604 *cm^{-1}*

LRMS (EI) m/z 337 $(M)^+$

HRMS (EI) calcd for $(M)^+$ $C_{19}H_{15}NO_3S$: 337.0773, found: 337.0774.

Rf = 0.18 (hexane/AcOEt: 3/1)

2-(Methylsulfonyl)-3-(thiophen-2-yl)isoindolin-1-one (2n)

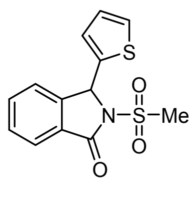

37% yield (27.5 mg, 0.0937 mmol)

colorless solid, **mp** 194 – 195 ℃ (recrystallized from hexane/AcOEt)

^{1}H NMR (400 MHz, $CDCl_3$/TMS) δ 7.95 (d, *J* = 7.7 Hz, 1H), 7.67 (td, *J* = 7.6, 1.2 Hz, 1H), 7.57 (t, *J* = 7.6 Hz, 1H), 7.35 (dd, *J* = 7.7, 0.9 Hz, 1H), 7.38 – 7.33 (m, 1H), 7.26 – 7.23 (m, 1H), 7.01 (dd, *J* = 5.1, 3.6 Hz, 1H), 6.52 (s, 1H), 3.11 (s, 3H).

$^{13}C\{^1H\}$ NMR (126 MHz, $CDCl_3$/TMS) δ 166.7, 146.0, 140.1, 134.8, 129.8, 129.0, 128.6, 127.3, 126.9, 125.2, 124.2, 60.6, 42.5.

IR (neat): 3121, 3005, 2928, 1719, 1352, 1279, 1165, 1103, 1086, 961, 847, 756, 698 *cm^{-1}*

LRMS (EI) m/z 293 $(M)^+$

HRMS (EI) calcd for $(M)^+$ $C_{13}H_{11}NO_3S_2$: 293.0180, found: 293.0182.

Rf = 0.19 (hexane/AcOEt: 3/1)

3-(1-Methyl-1*H*-indol-3-yl)-2-(methylsulfonyl)isoindolin-1-one (2o)

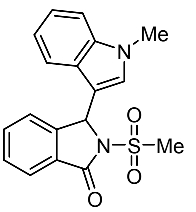

95% yield (80.8 mg, 0.237 mmol)

colorless solid, **mp** 241 – 242 ℃ (recrystallized from hexane/AcOEt)

^{1}H NMR (400 MHz, $CDCl_3$/TMS) δ 8.05 – 8.00 (m, 1H), 7.61 – 7.51 (m, 2H), 7.35 (s, 1H), 7.31 – 7.25 (m, 2H), 7.19 – 7.10 (m, 1H), 6.90 – 6.81 (m, 1H), 6.59 (d, *J* = 8.1 Hz, 1H), 6.46 (s, 1H), 3.81 (s, 3H), 2.86 (s, 3H).

^{13}C{^{1}H} NMR (101 MHz, $CDCl_3$/TMS) δ 167.3, 146.5, 137.6, 134.6, 131.0, 129.4, 129.3, 125.1, 124.9, 124.2, 122.3, 120.2, 118.8, 110.0, 108.5, 60.1, 42.2, 33.1.

IR (neat): 3123, 3073, 2924, 1730, 1611, 1549, 1466, 1346, 1333, 1319, 1294, 1277, 1254, 1159, 1099, 1070, 1016, 962, 756, 737, 702, 685, 665 *cm^{-1}*

LRMS (EI) m/z 340 $(M)^+$

HRMS (EI) calcd for $(M)^+$ $C_{18}H_{16}N_2O_2S$: 340.0882, found: 340.0883.

Rf = 0.21 (hexane/AcOEt: 1/1 with 0.5% NEt_3)

3-(1-Acetyl-1*H*-indol-3-yl)-2-(methylsulfonyl)isoindolin-1-one (2p)

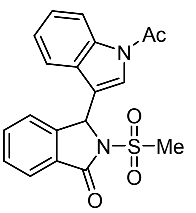

74% yield (68.2 mg, 0.185 mmol)

colorless solid, **mp** 222 ℃ (recrystallized from hexane/AcOEt)

^{1}H NMR (500 MHz, $CDCl_3$/TMS) δ 8.44 (d, *J* = 8.4 Hz, 1H), 8.05 (dd, *J* = 6.4, 1.7 Hz, 1H), 7.75 (s, 1H), 7.67 – 7.51 (m, 2H), 7.37 – 7.24 (m, 2H), 7.08 – 7.00 (m, 1H), 6.65 (d, *J* = 8.0 Hz, 1H), 6.42 (s, 1H), 3.03 (s, 3H), 2.70 (s, 3H).

^{13}C{^{1}H} NMR (101 MHz, $CDCl_3$/TMS) δ 168.6, 167.2, 145.0, 136.6, 135.0, 129.8, 129.3, 126.9, 126.7, 125.9, 125.3, 124.2, 124.0, 118.7, 117.3, 116.8, 59.0, 42.2, 24.1.

IR (neat):3115, 3051, 3017, 2934, 1728, 1717, 1701, 1449, 1391, 1360, 1342, 1323, 1281, 1227, 1165, 1123, 1096, 1078, 1016, 961, 941, 835, 750, 733, 696, 646 *cm^{-1}*

LRMS (EI) m/z 368 $(M)^+$

HRMS (EI) calcd for $(M)^+$ $C_{19}H_{16}N_2O_4S$: 368.0831, found: 368.0833.

Rf = 0.14 (hexane/AcOEt: 1/1)

2-(Methylsulfonyl)-3-(1-tosyl-1*H*-indol-3-yl)isoindolin-1-one (2q)

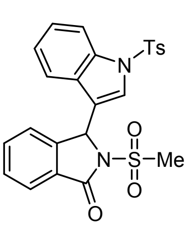

23% yield (27.4 mg, 0.0570 mmol)

colorless oil

^{1}H NMR (500 MHz, $CDCl_3$/TMS) δ 8.02 (d, *J* = 6.9 Hz, 1H), 7.95 (d, *J* = 8.4 Hz, 1H), 7.90 (s, 1H), 7.78 (d, *J* = 8.3 Hz, 2H), 7.65 – 7.54 (m, 2H), 7.26 – 7.19 (m, 4H), 6.98 (t, *J* = 7.6 Hz, 1H), 6.57 (d, *J* = 8.0 Hz, 1H), 6.41 (s, 1H), 2.79 (s, 3H), 2.35 (s, 3H).

^{13}C{^{1}H} NMR (101 MHz, $CDCl_3$/TMS) δ 167.0, 145.4, 144.8, 135.7, 135.02, 134.96, 130.1, 129.8, 129.2, 128.5, 127.5, 127.1, 125.3, 125.2, 124.0, 123.9, 119.3, 116.9, 114.4, 58.7, 42.0, 21.7.

IR (neat): 3111, 3026, 2928, 1728, 1597, 1468, 1447, 1356, 1325, 1290, 1256, 1217, 1165, 1094, 1018, 966, 835, 812, 799, 745, 729, 694, 669, 650, 631 *cm^{-1}*

LRMS (EI) m/z 480 $(M)^+$

HRMS (EI) calcd for $(M)^+$ $C_{24}H_{20}N_2O_5S_2$: 480.0814, found: 480.0810.

Rf = 0.22 (hexane/AcOEt: 2/1 with 0.5% NEt_3)

3-Phenylisoindolin-1-one (3)

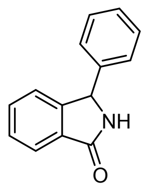

Bu_3SnH (0.27 mL, 1.0 mmol) and AIBN (24.6 mg, 0.15 mmol) were added to a solution of isoindolinone **2f** (71.8 mg, 0.25 mmol) in toluene (5 mL) in one portion at rt. The reaction was refluxed under Ar atmosphere and then AIBN (8.2 mg, 0.05 mmol) was added portionwise every 0.5 h (5 times). After cooling to rt, the reaction mixture was quenched with 1M HCl aq. and taken up in AcOEt. The organic layer was separated, washed with H_2O and brine, dried over $MgSO_4$, filtered, and concentrated *in vacuo*. The residue was purified by silica gel flash column chromatography eluting with hexane/AcOEt to afford **3** in 95% yield (49.7 mg, 0.238 mmol) as a colorless solid.

mp 222 – 223 ℃ (recrystallized from hexane/AcOEt)

^{1}H NMR (400 MHz, $CDCl_3$/TMS) δ 7.93 – 7.87 (m, 1H), 7.54 – 7.44 (m, 2H), 7.39 – 7.30 (m, 3H), 7.29 – 7.21 (m, 3H), 6.41 (brs, 1H), 5.62 (s, 1H).

^{13}C{^{1}H} NMR (101 MHz, $CDCl_3$/TMS) δ 170.9, 148.1, 138.6, 132.5, 131.0, 129.3, 128.8, 128.6, 127.0, 124.0, 123.5, 60.9.

IR (neat): 3154, 3055, 2864, 1682, 1454, 1362, 1319, 1140, 789, 741, 723, 694, 617, 575 *cm^{-1}*

LRMS (EI) m/z 209 $(M)^+$

HRMS (EI) calcd for $(M)^+$ $C_{14}H_{11}NO$: 209.0841, found: 209.0837.

Rf = 0.09 (hexane/AcOEt: 2/1)

2-(Methylsulfonyl)-1-phenylisoindoline (4)

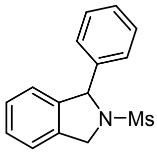

To a solution of isoindolinone **2f** (57.5 mg, 0.20 mmol) in THF (2 mL), $LiAlH_4$ (22.8 mg, 0.6 mmol, 3 eq.) was added slowly at 0 °C. The reaction was warmed to rt and stirred for 1 h. The reaction was quenched with 1M HCl aq. and taken up in AcOEt. The organic layer was separated and the aqueous layer was extracted with AcOEt (twice). The collected organic layer was washed with brine, dried over $MgSO_4$, filtered, and concentrated *in vacuo*. The residue was purified by silica gel flash column chromatography eluting with hexane/AcOEt to afford **4** in 97% yield (53.2 mg, 0.195 mmol) as colorless oil.

mp 193 – 195 ℃ (recrystallized from hexane/AcOEt)

^{1}H NMR (400 MHz, $CDCl_3$/TMS) δ 7.42 – 7.26 (m, 9H), 6.04 (s, 1H), 4.63 (d, *J* = 12.2 Hz, 1H), 4.51 (d, *J* = 12.2 Hz, 1H), 2.69 (s, 3H).

^{13}C{^{1}H} NMR (101 MHz, $CDCl_3$/TMS) δ 140.5, 139.9, 138.0, 131.0, 129.3, 128.9, 128.9, 128.7, 127.9, 127.2, 63.5, 59.0, 42.0.

IR (neat): 3275, 3028, 2889, 1450, 1310, 1250, 1146, 1107, 1043, 1028, 978, 910, 833, 746, 698, 611, 602, 561 *cm^{-1}*

LRMS (EI) m/z 273 $(M)^+$

HRMS (EI) calcd for $(M)^+$ $C_{15}H_{15}NO_2S$: 273.0823, found: 273.0822.

Rf = 0.34 (hexane/AcOEt: 1/1)

5. References

1. Q. Elliott, G. dos Passos Gomes, C. J. Evoniuk, I. V. Alabugin, *Chem. Sci.*, 2020, **11**, 6539.

2. L. Zhang, G. Y. Ang, S. Chiba, *Org. Lett.*, 2011, **13**, 1622.

3. Y. Wang, Shi. Yang, G. Bian, L. Song, *Synthesis*, 2017, **49**, 1884.

4. A. K. Turek, D. J. Hardee, A. M. Ullman, D. G. Nocera, E. N. Jacobsen, *Angew. Chem. Int. Ed.*, 2016, **55**, 539.

5. T. Duhamel, K. Muniz, *Chem. Commun.*, 2019, **55**, 933.

6. R. B. Bedford J. G. Bowen, C. Méndez-Gálvez, *J. Org. Chem.*, 2017, **82**, 1719.

^{1}H-, ^{13}C-and ^{19}F-NMR Spectra

^{1}H NMR (400 MHz, $CDCl_3$) of 1a

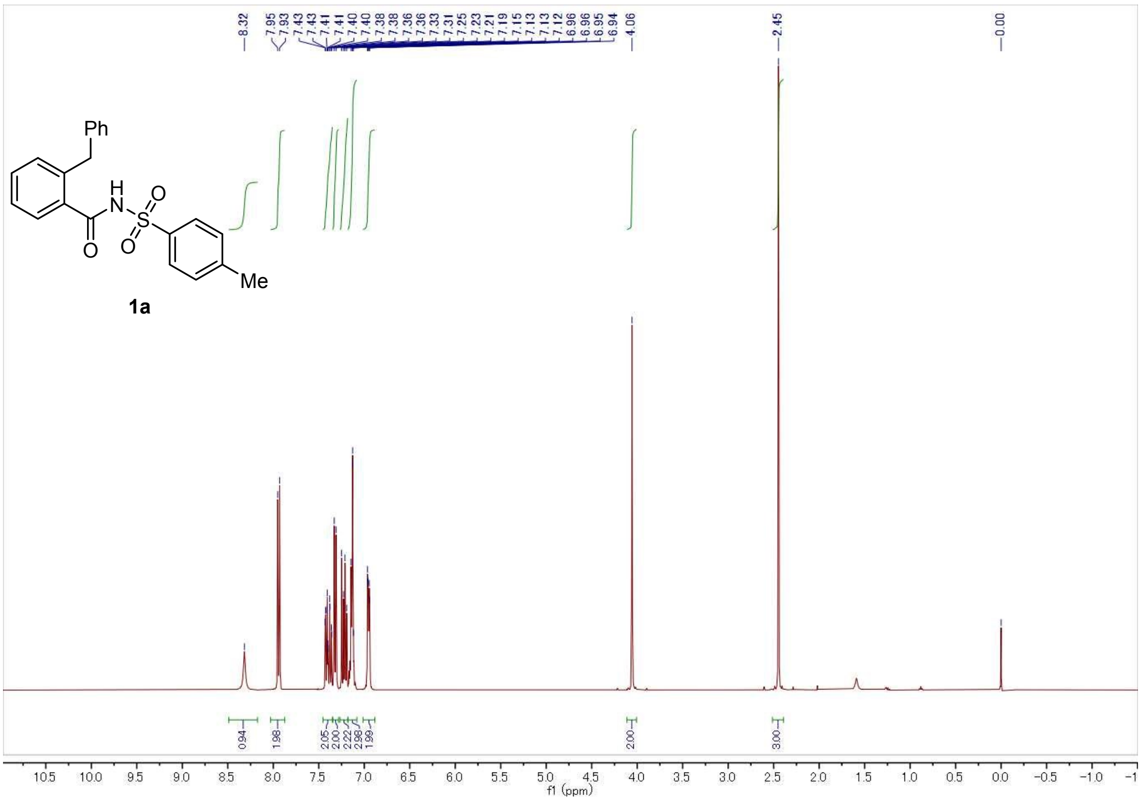

^{13}C NMR (101 MHz, $CDCl_3$) of 1a

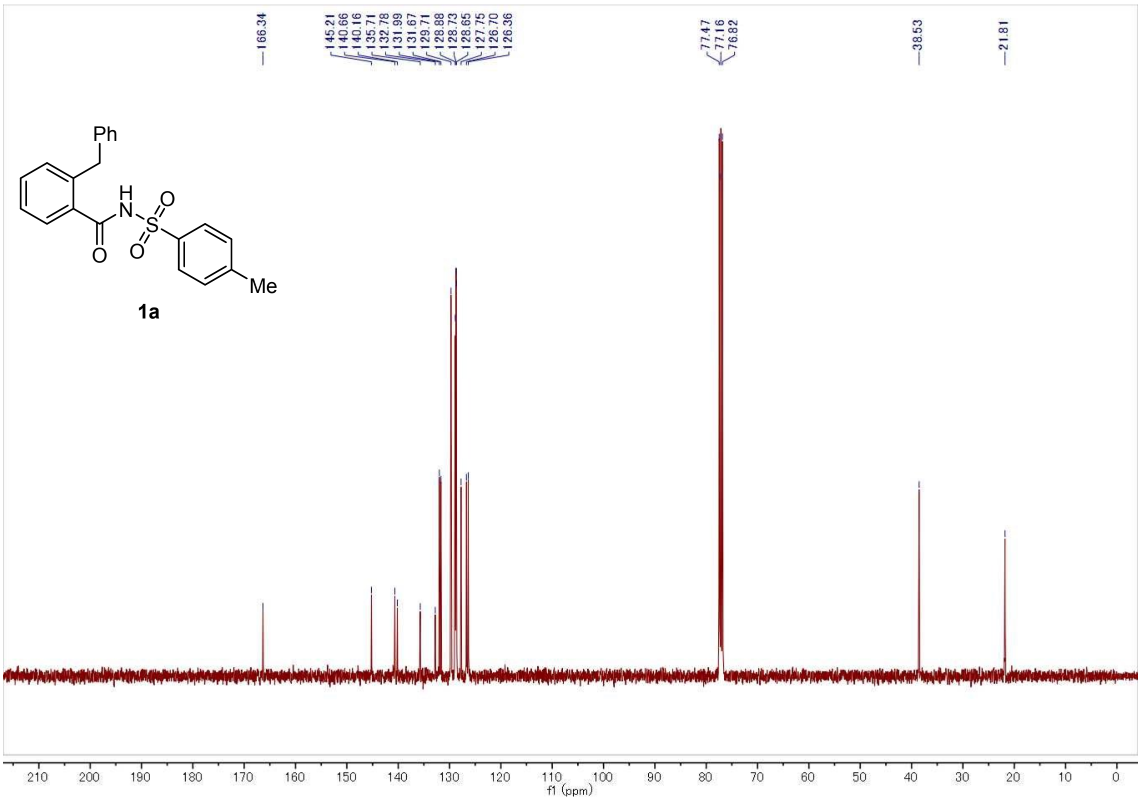

^{1}H NMR (500 MHz, $CDCl_3$) of 1b

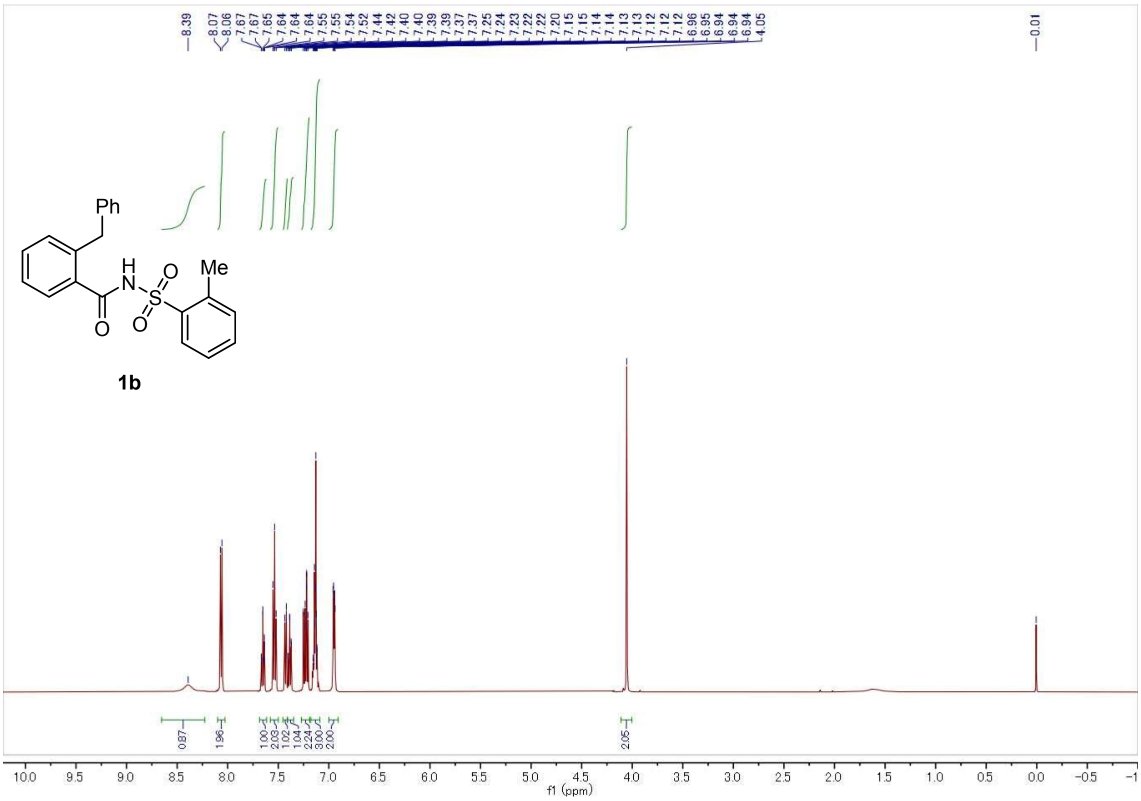

^{13}C NMR (126 MHz, $CDCl_3$) of 1b

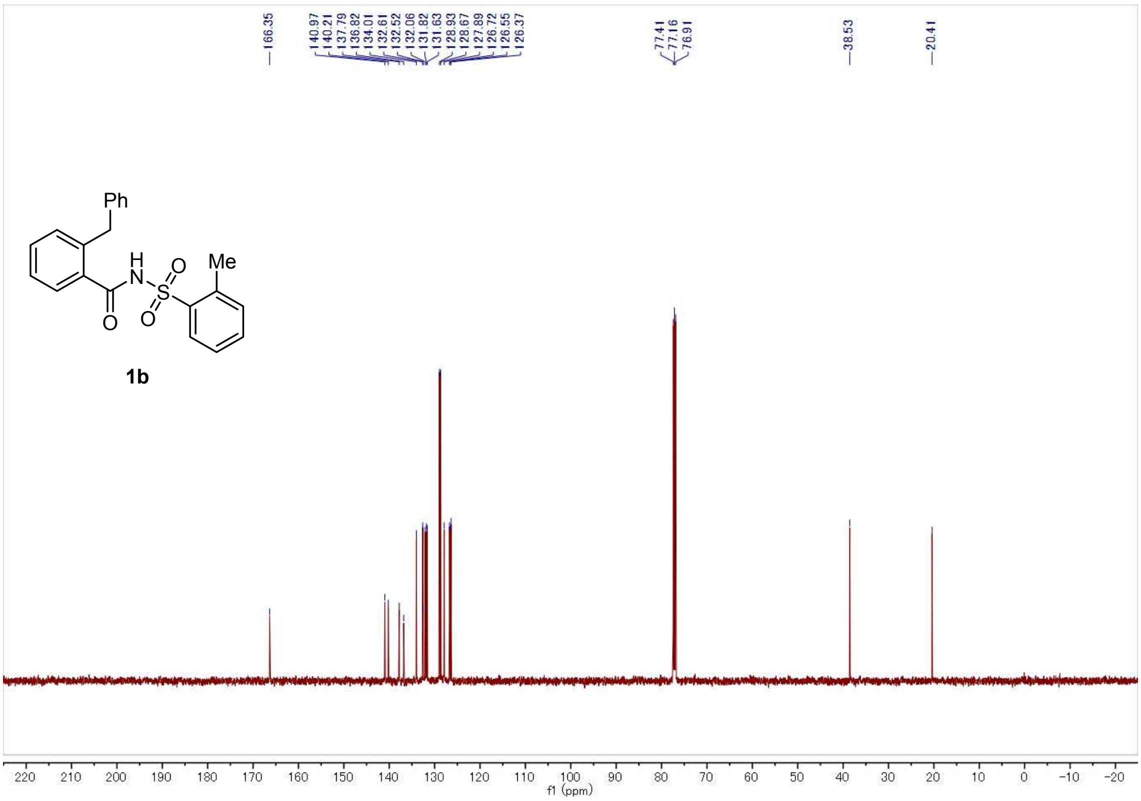

^{1}H NMR (500 MHz, $CDCl_3$) of 1c

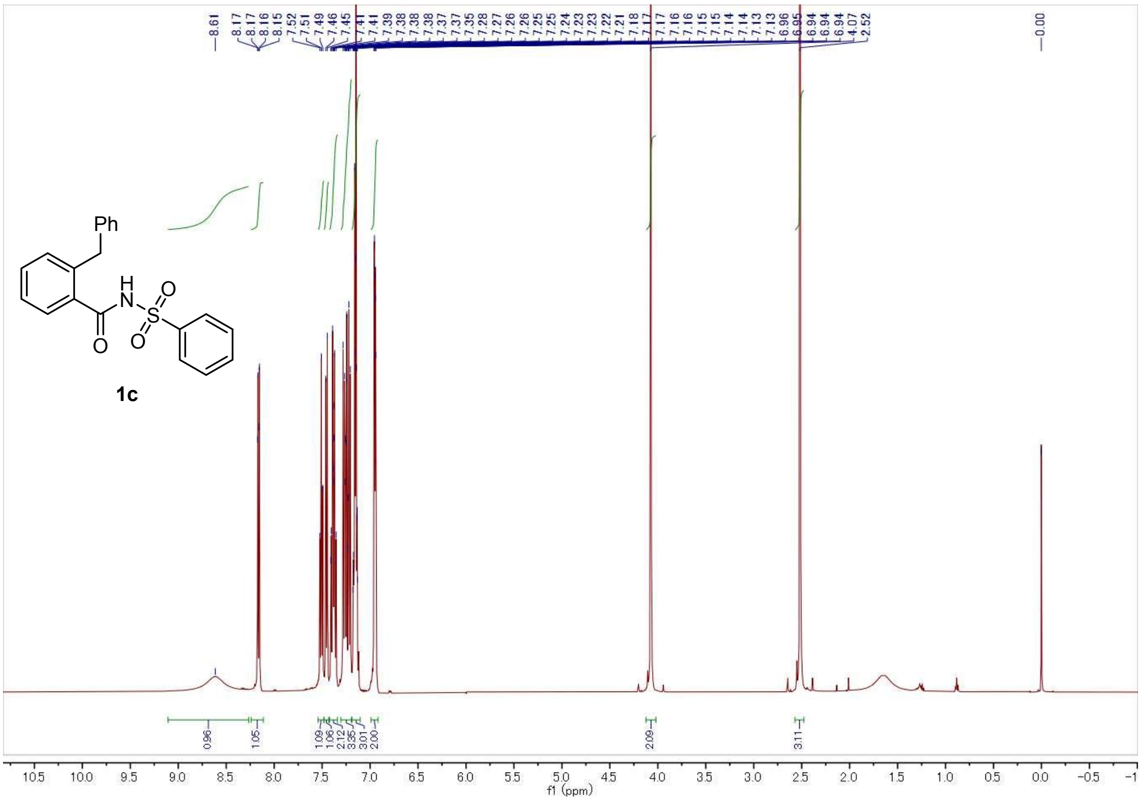

^{13}C NMR (126 MHz, $CDCl_3$) of 1c

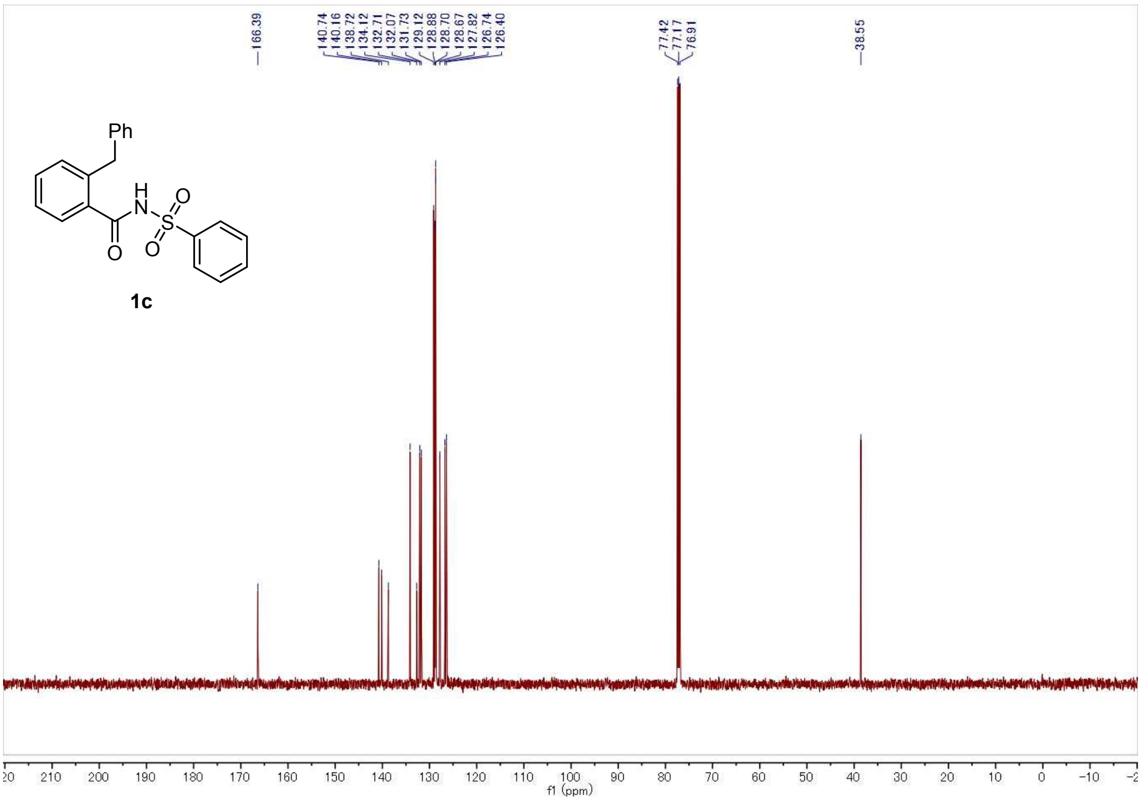

^{1}H NMR (500 MHz, $CDCl_3$) of 1d

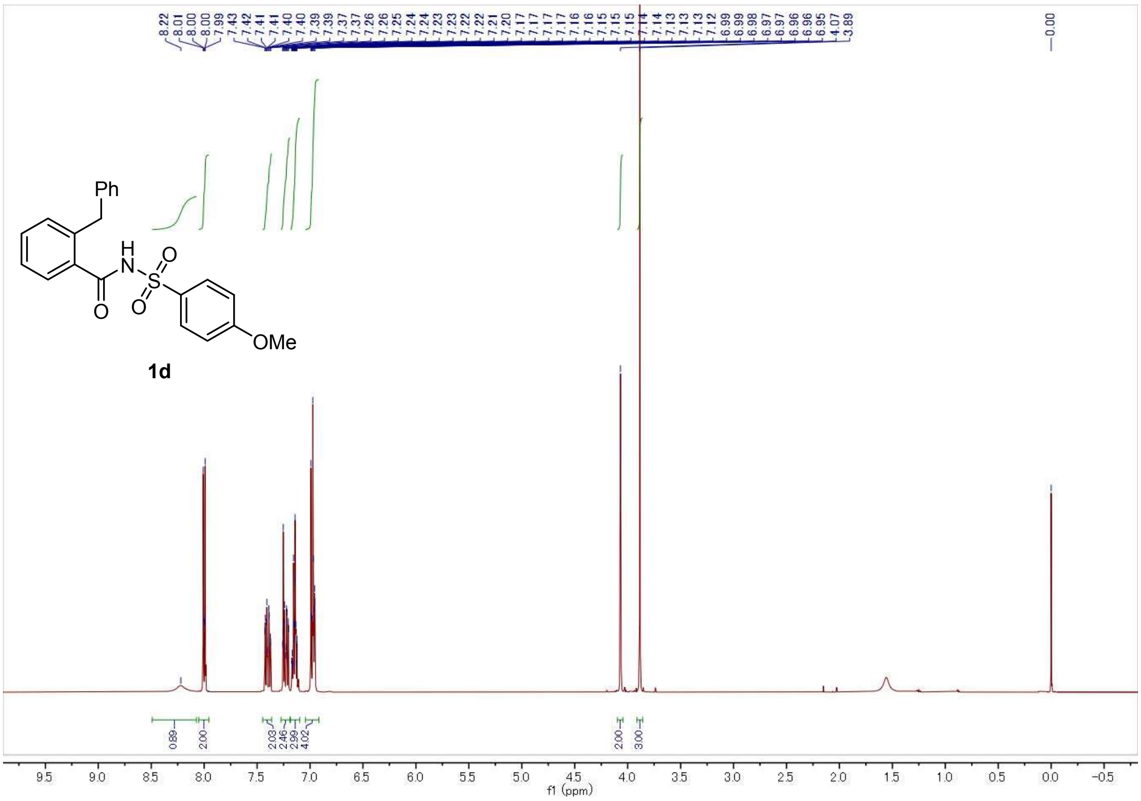

^{13}C NMR (126 MHz, $CDCl_3$) of 1d

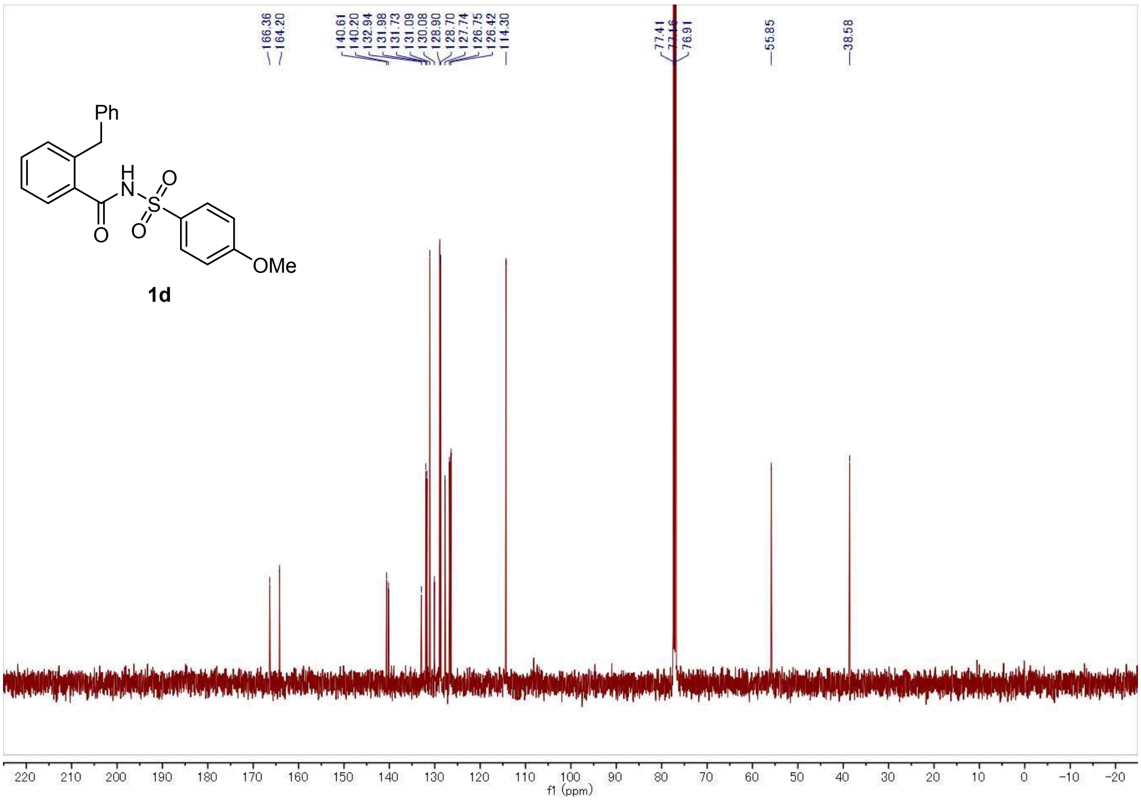

^{1}H NMR (500 MHz, DMSO-d_6) of 1e

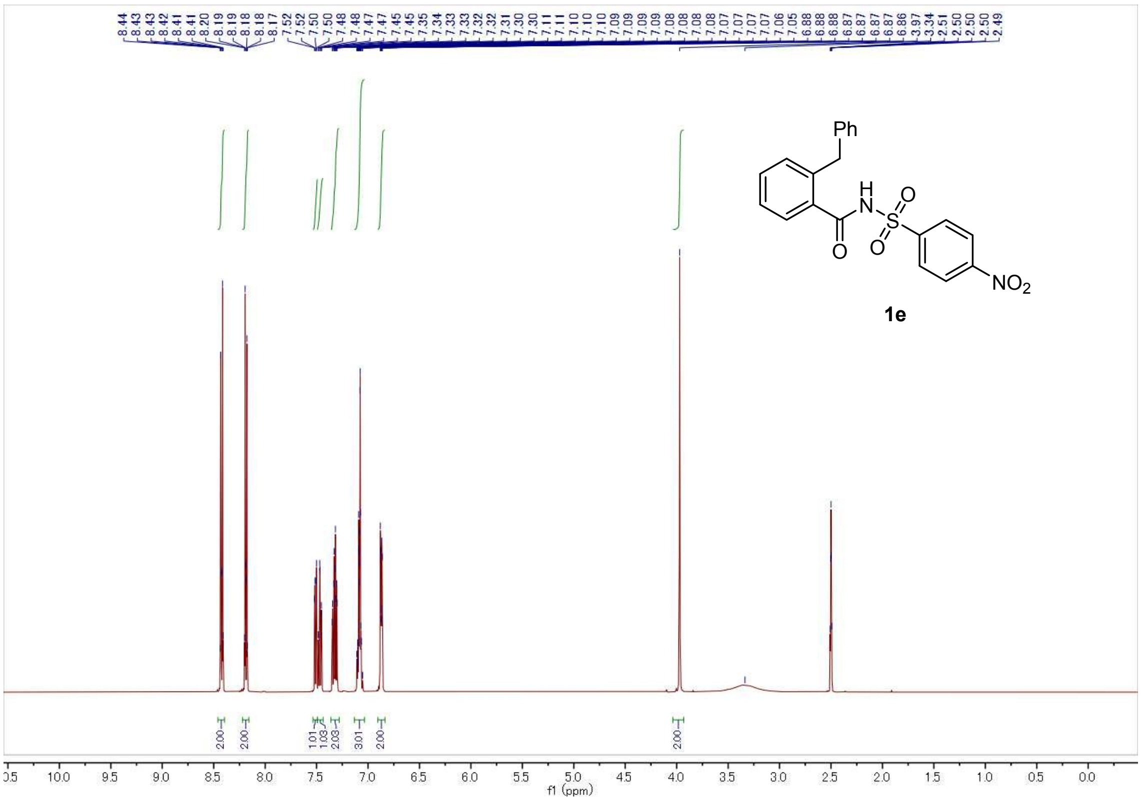

^{13}C NMR (126 MHz, DMSO-d_6) of 1e

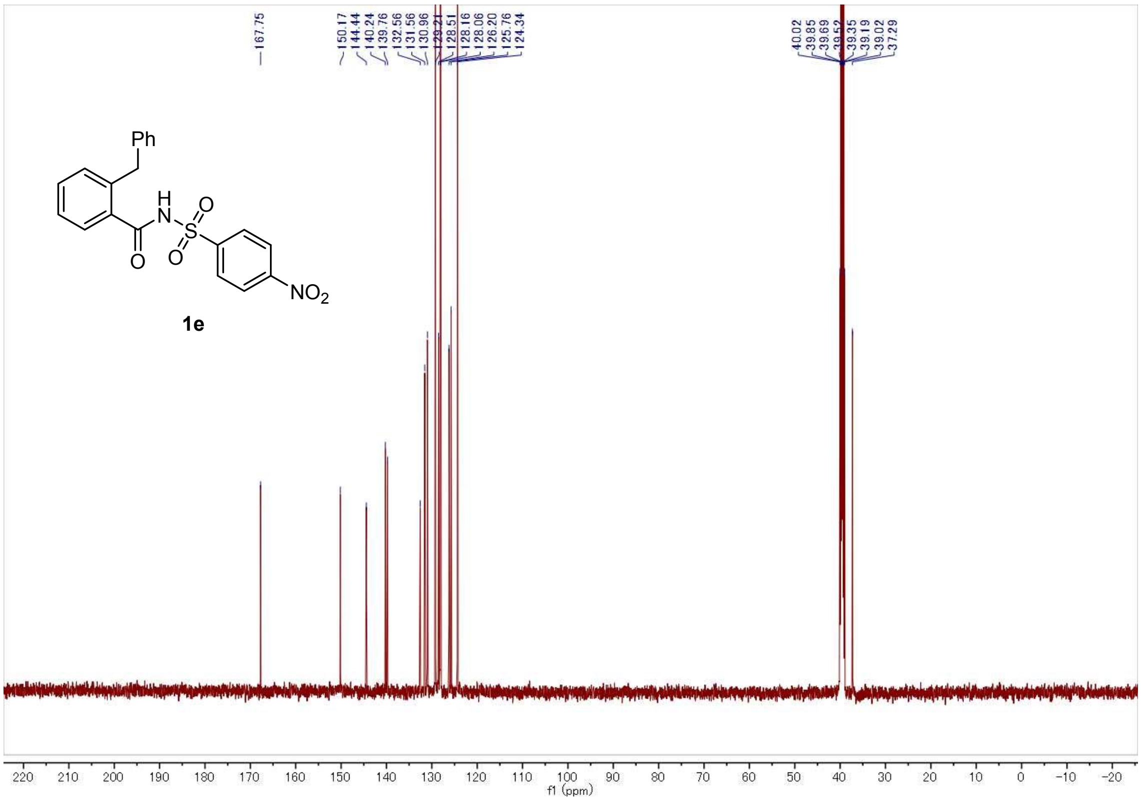

^{1}H NMR (500 MHz, $CDCl_3$) of 1f

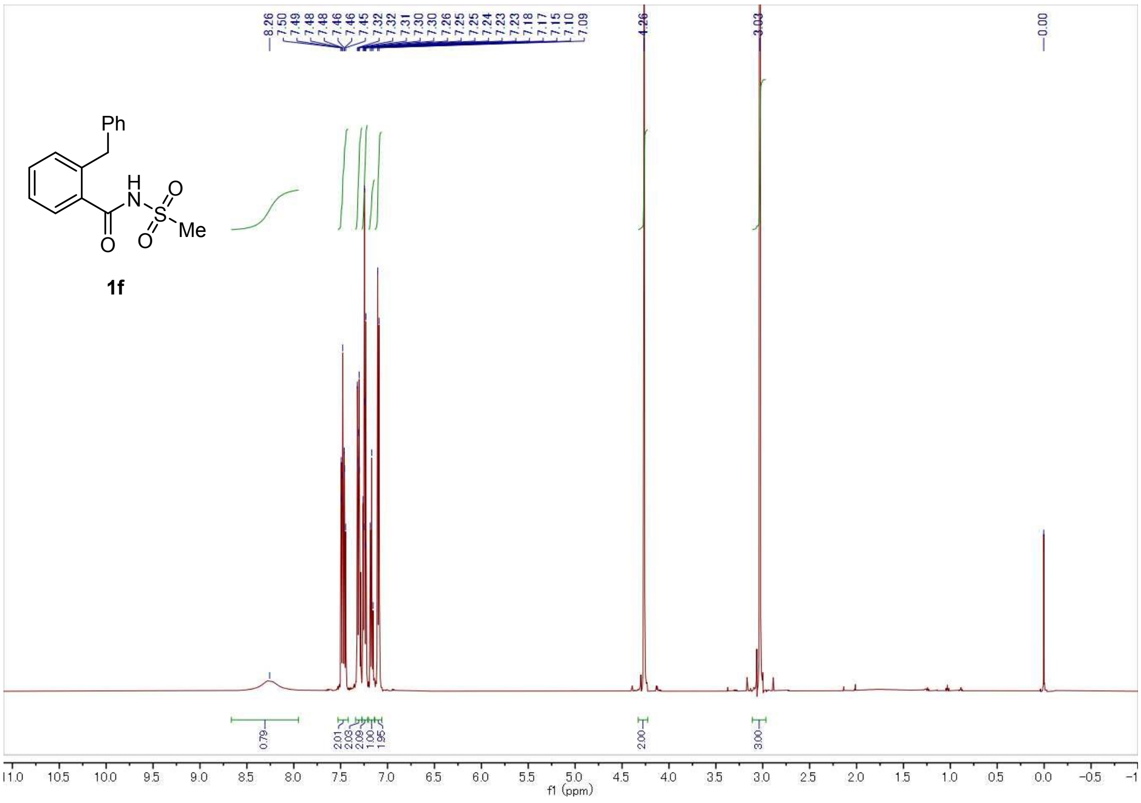

^{13}C NMR (126 MHz, $CDCl_3$) of 1f

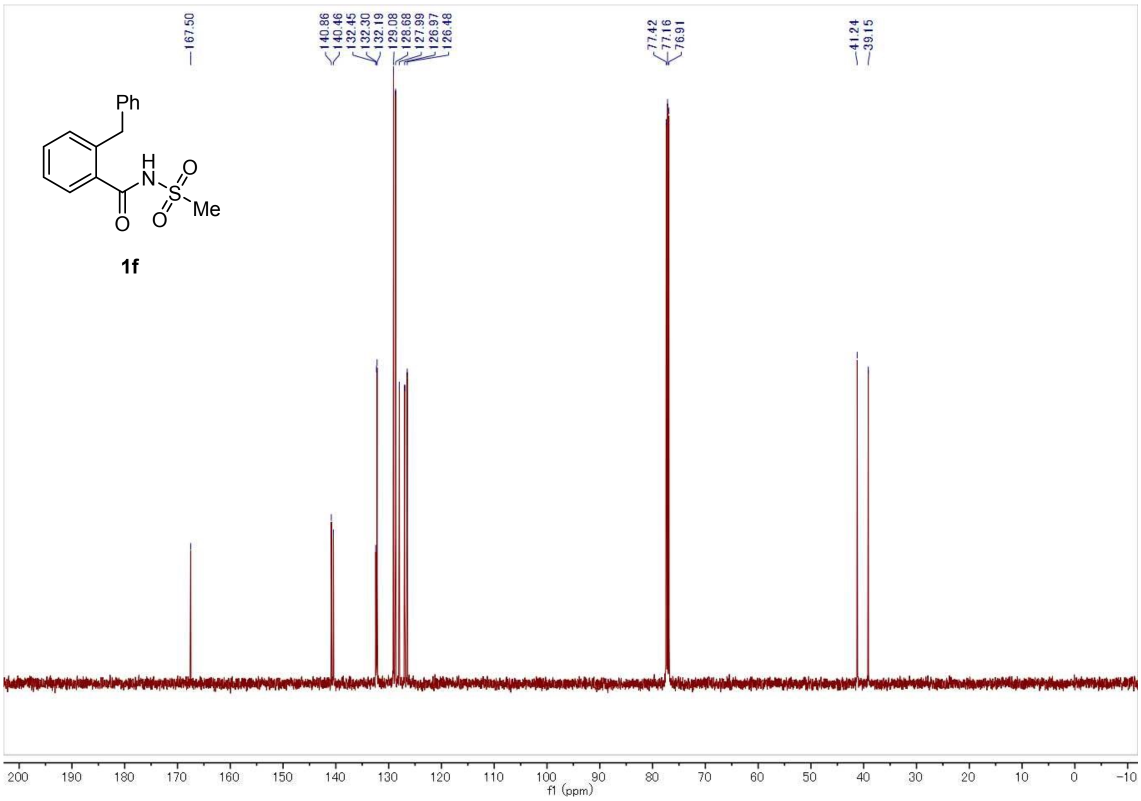

^{1}H NMR (400 MHz, $CDCl_3$) of 1g

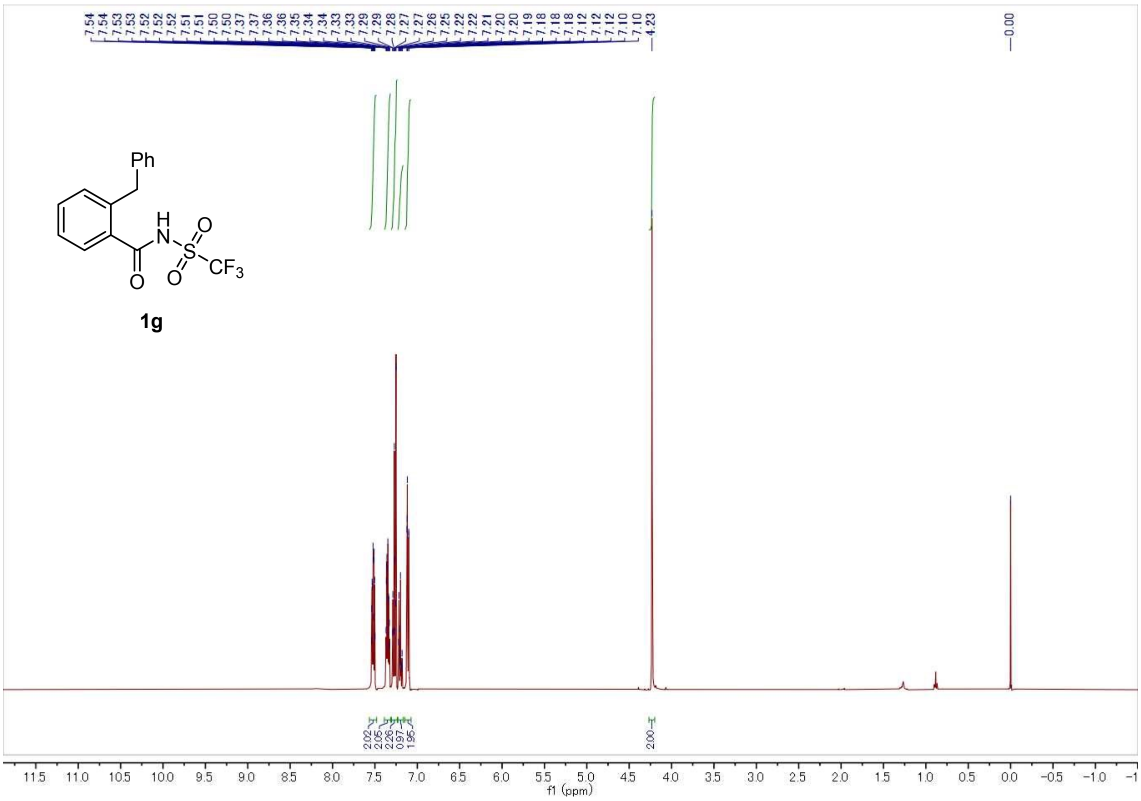

^{13}C NMR (101 MHz, $CDCl_3$) of 1g

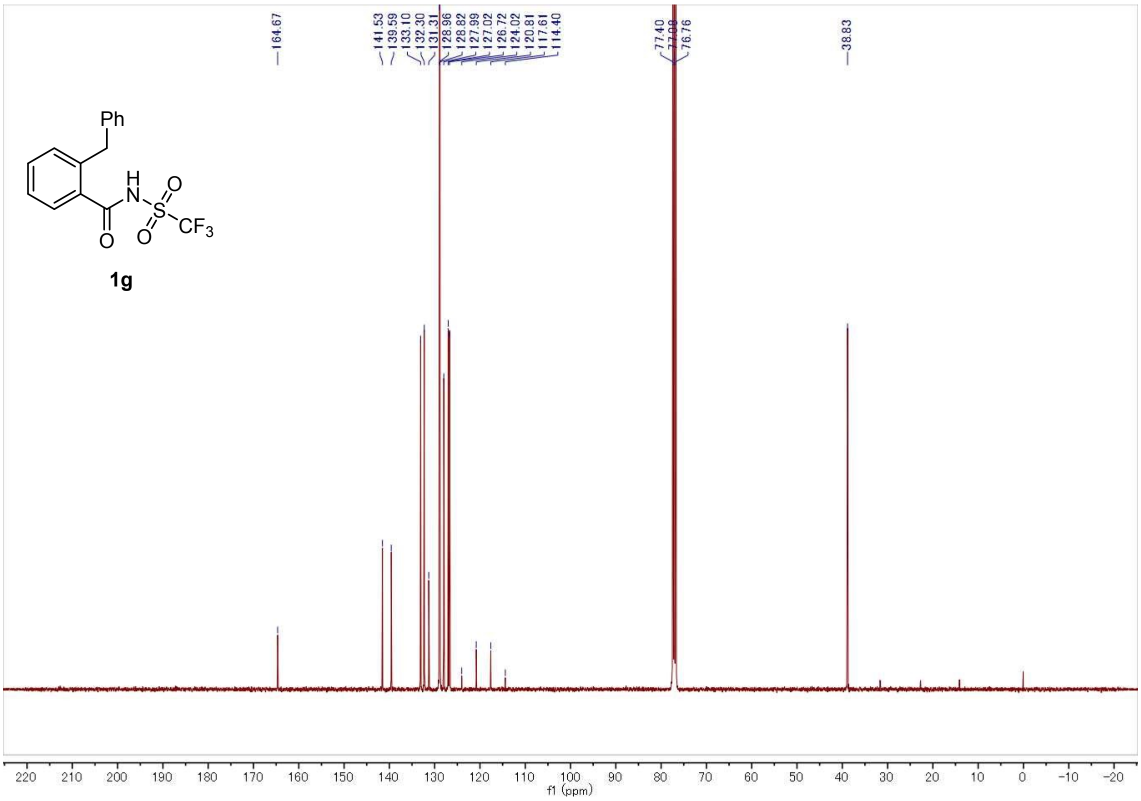

^{19}F NMR (471 MHz, $CDCl_3$) of 1g

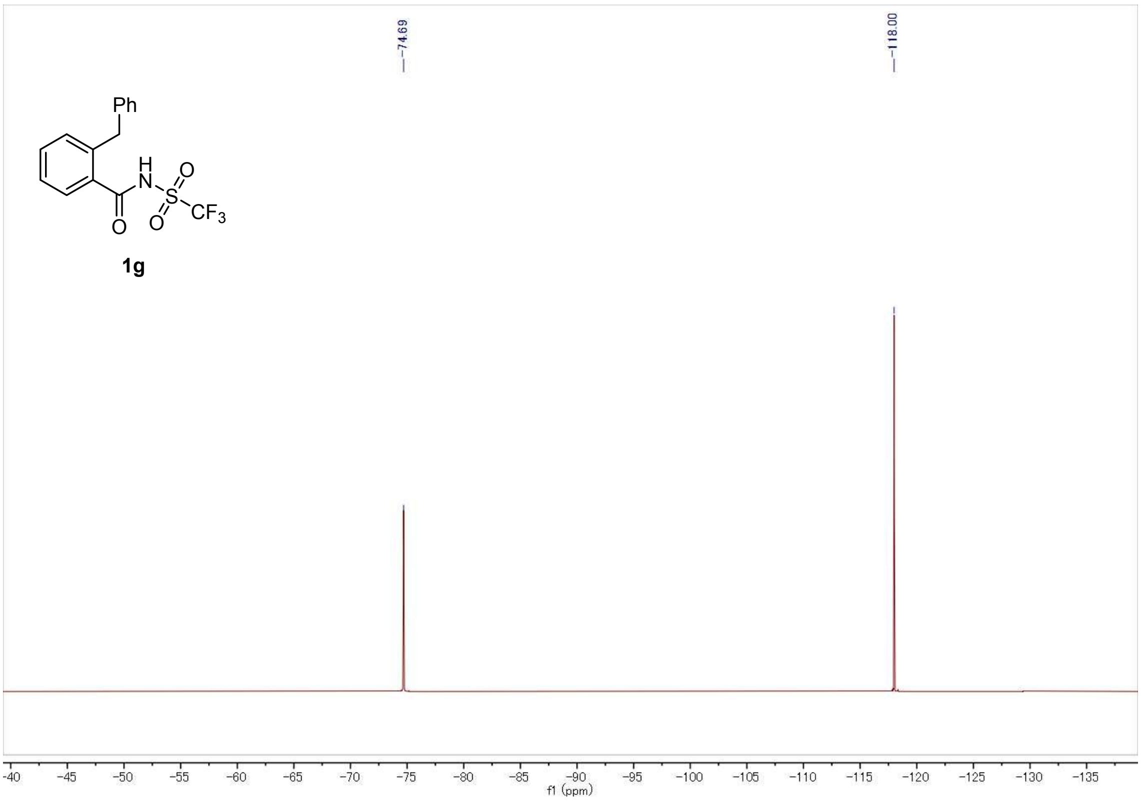

^{1}H NMR (500 MHz, $CDCl_3$) of 1j

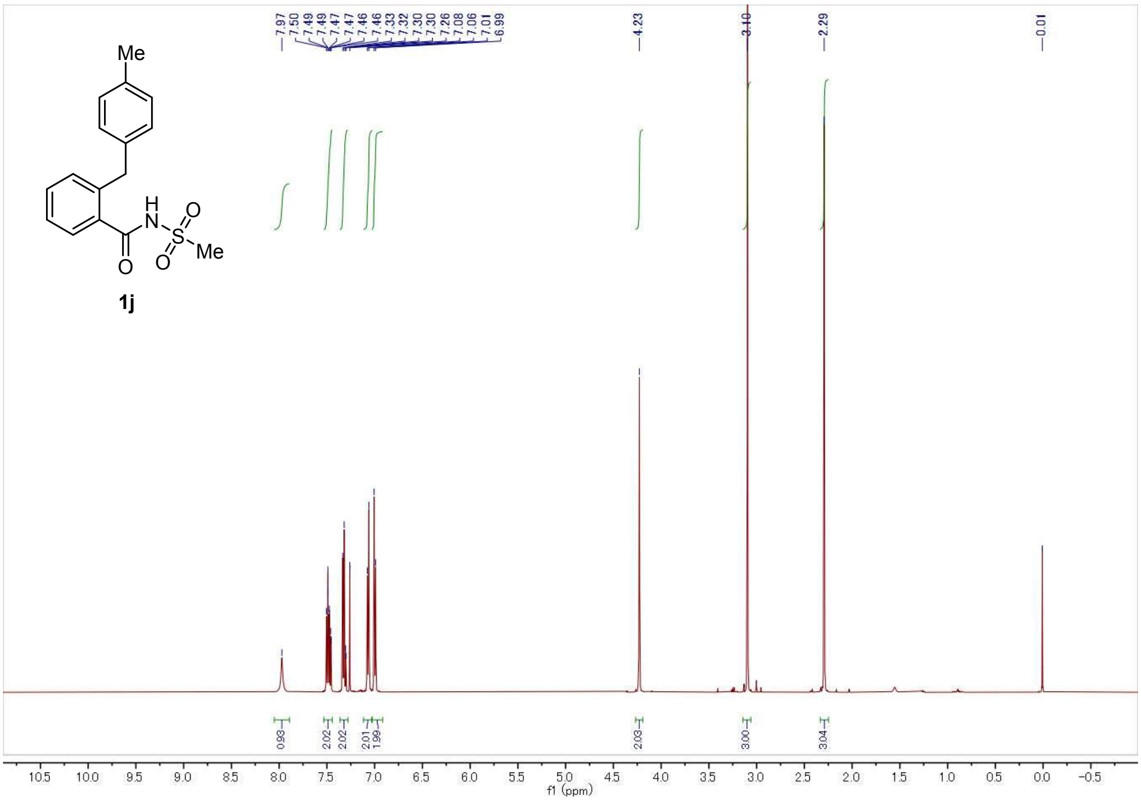

^{13}C NMR (126 MHz, $CDCl_3$) of 1j

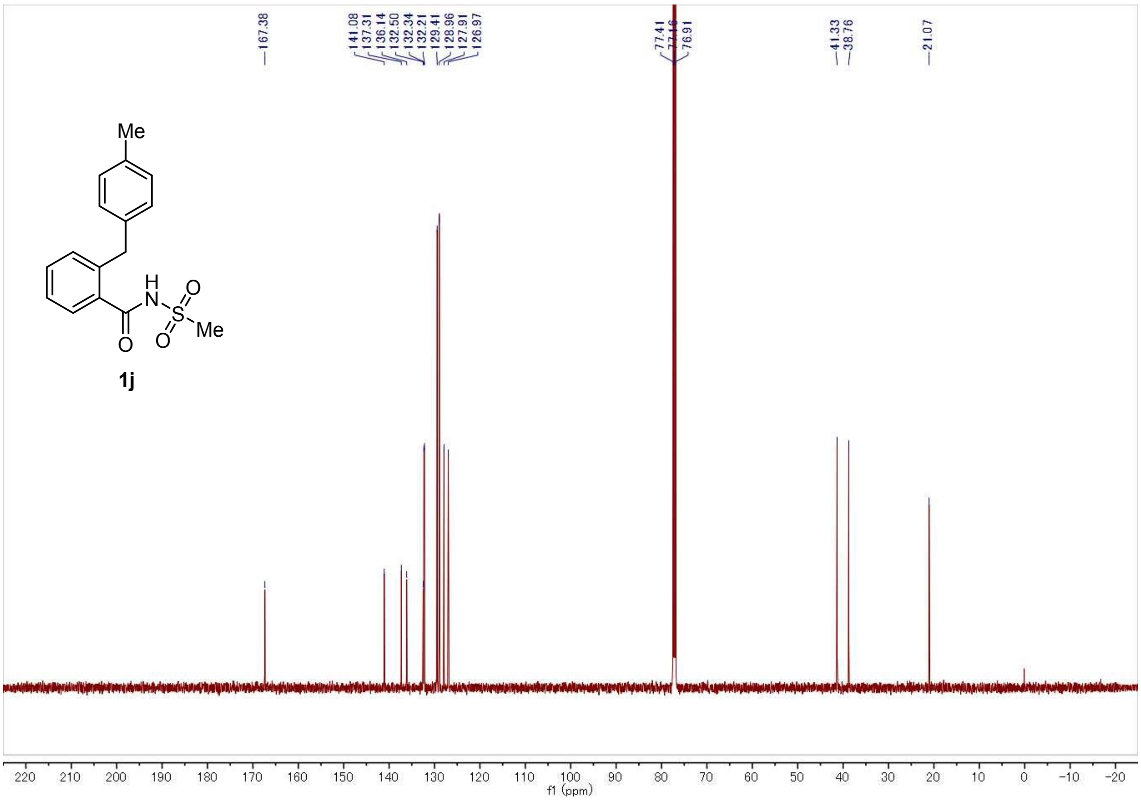

^{1}H NMR (500 MHz, $CDCl_3$) of 1k

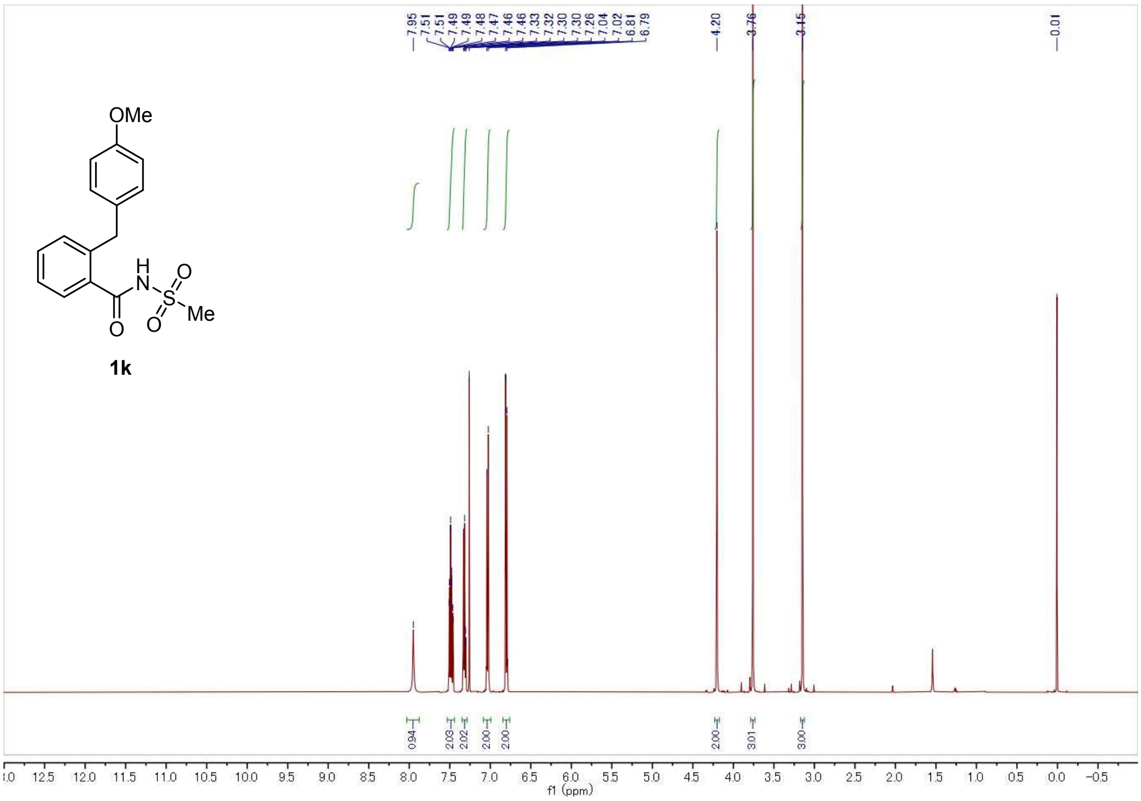

^{13}C NMR (126 MHz, $CDCl_3$) of 1k

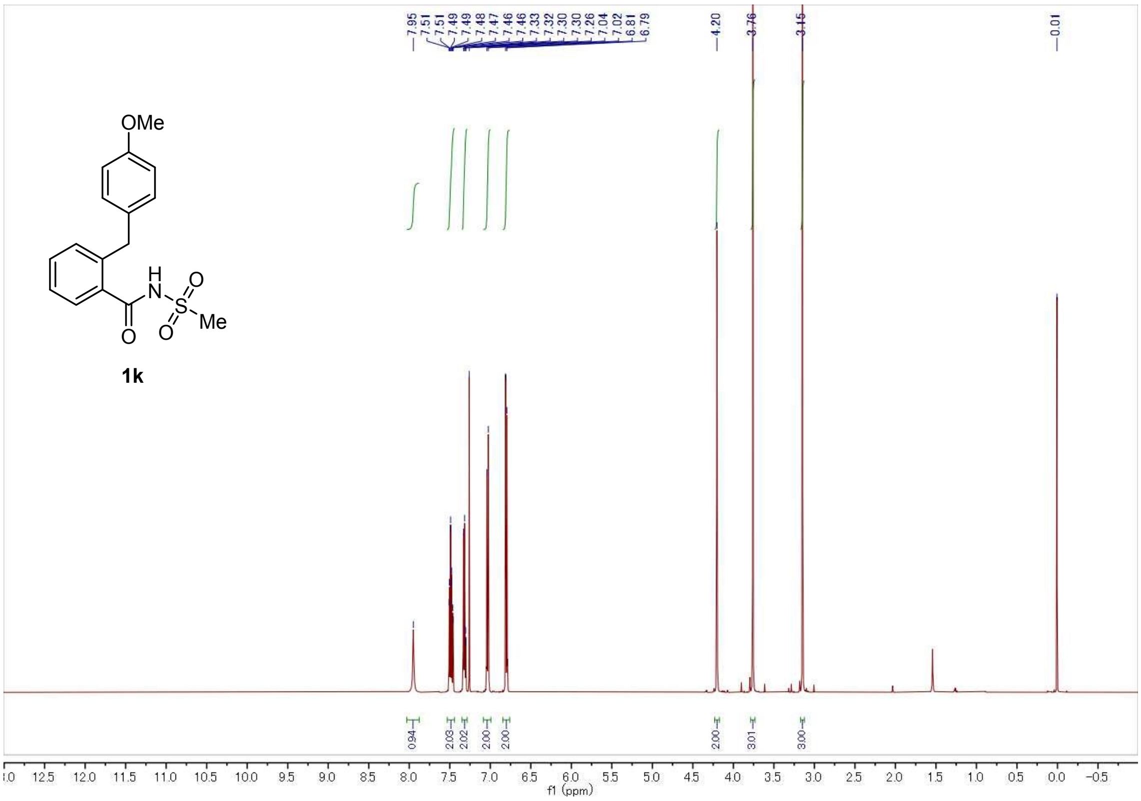

^{1}H NMR (500 MHz, $CDCl_3$) of 1l

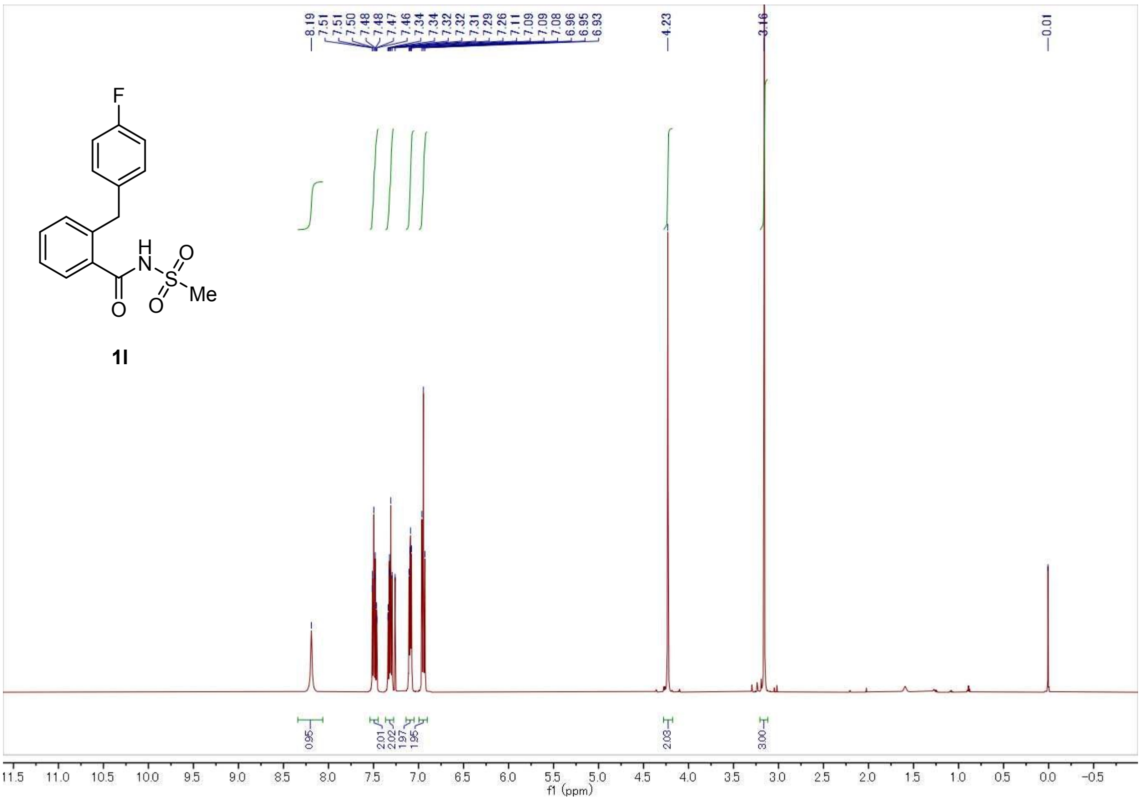

^{13}C NMR (126 MHz, $CDCl_3$) of 1l

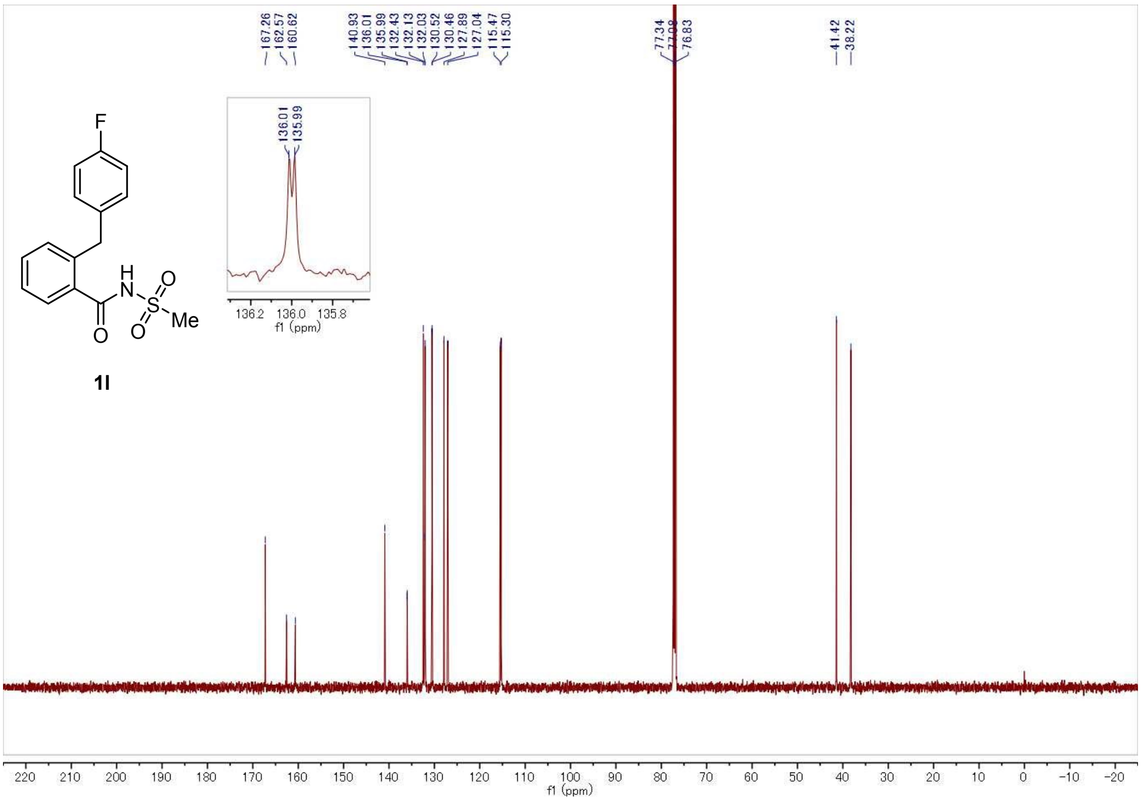

^{19}F NMR (471 MHz, CDCl$_3$) of 1l

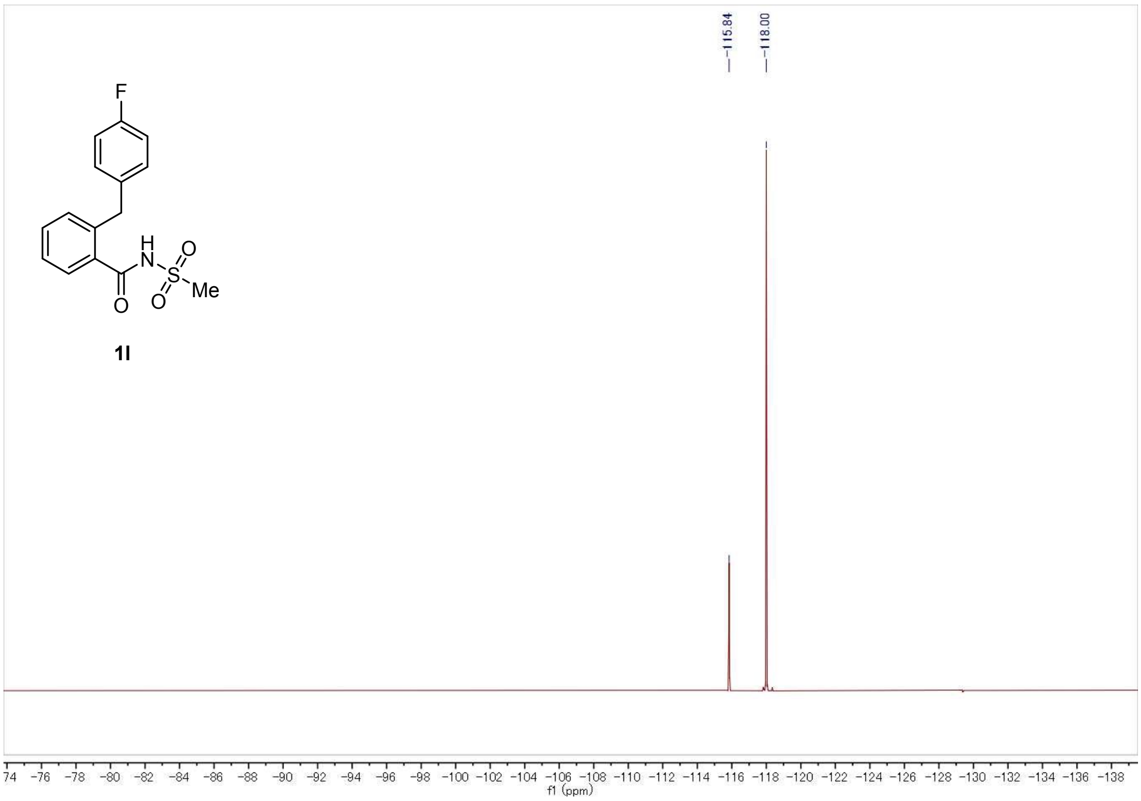

^1H NMR (400 MHz, $CDCl_3$) of 1m

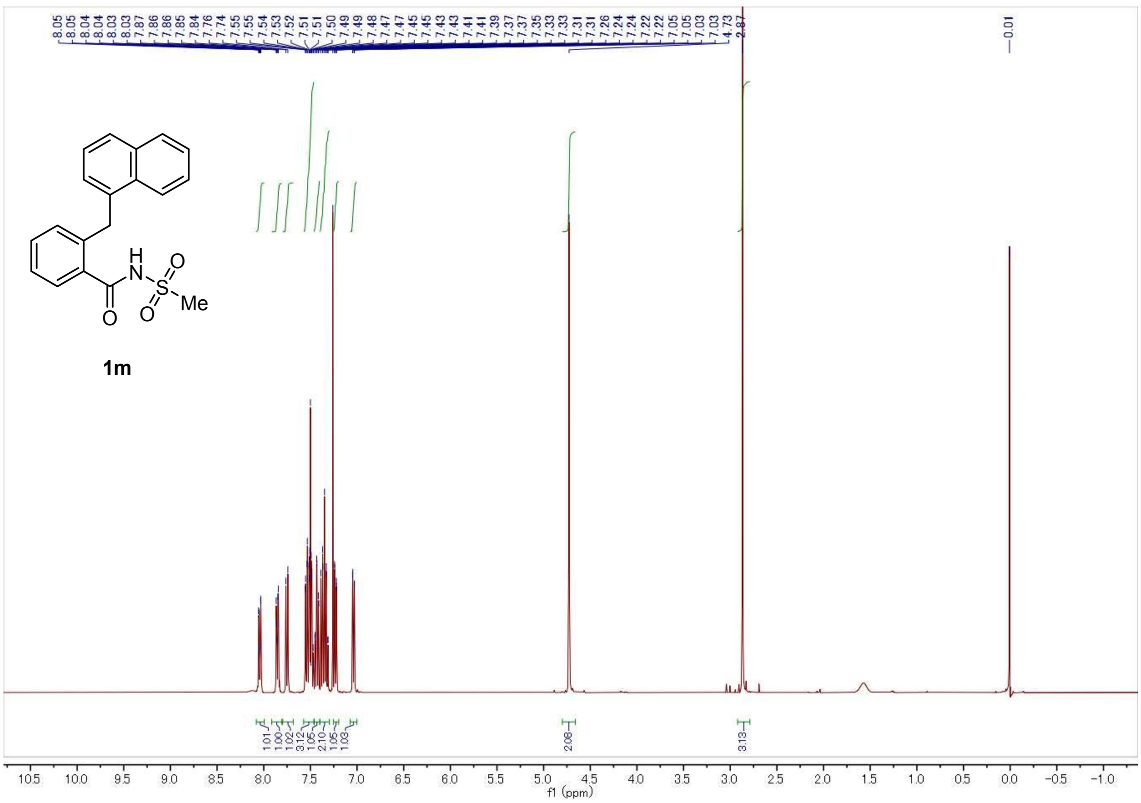

^{13}C NMR (101 MHz, $CDCl_3$) of 1m

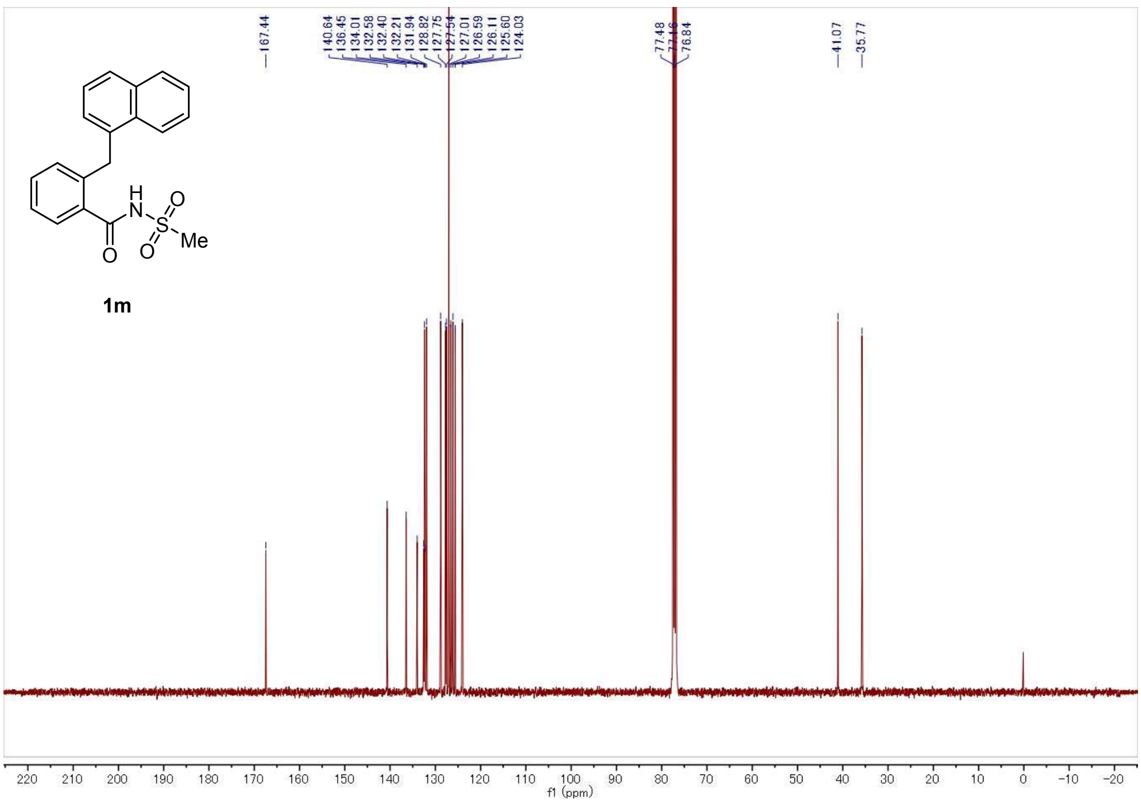

^{1}H NMR (500 MHz, $CDCl_3$) of 1n

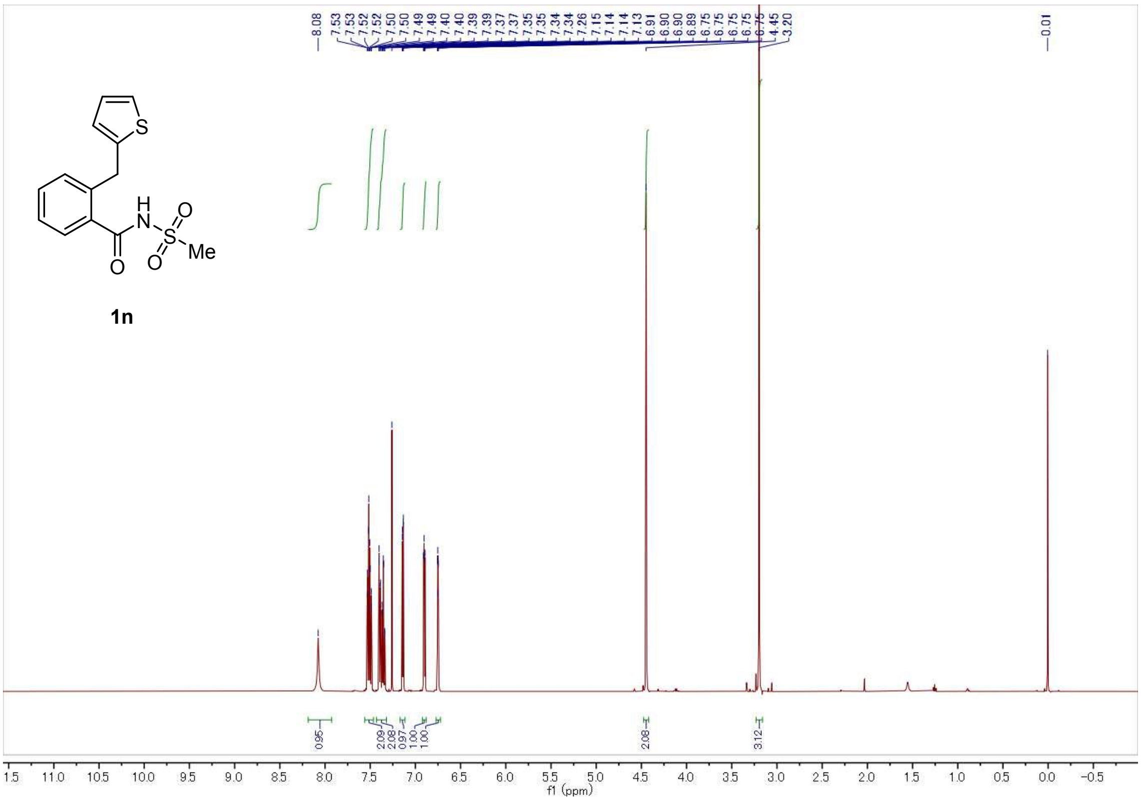

^{13}C NMR (126 MHz, $CDCl_3$) of 1n

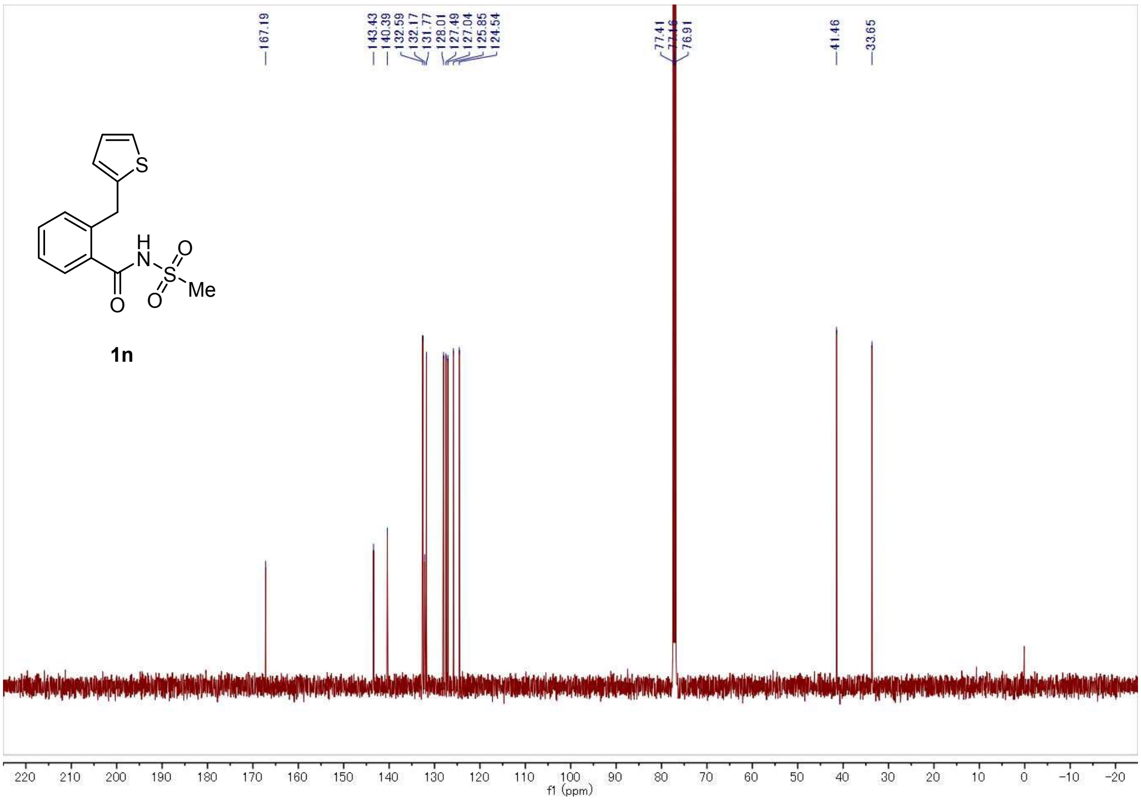

^{1}H NMR (400 MHz, $CDCl_3$) of 1o

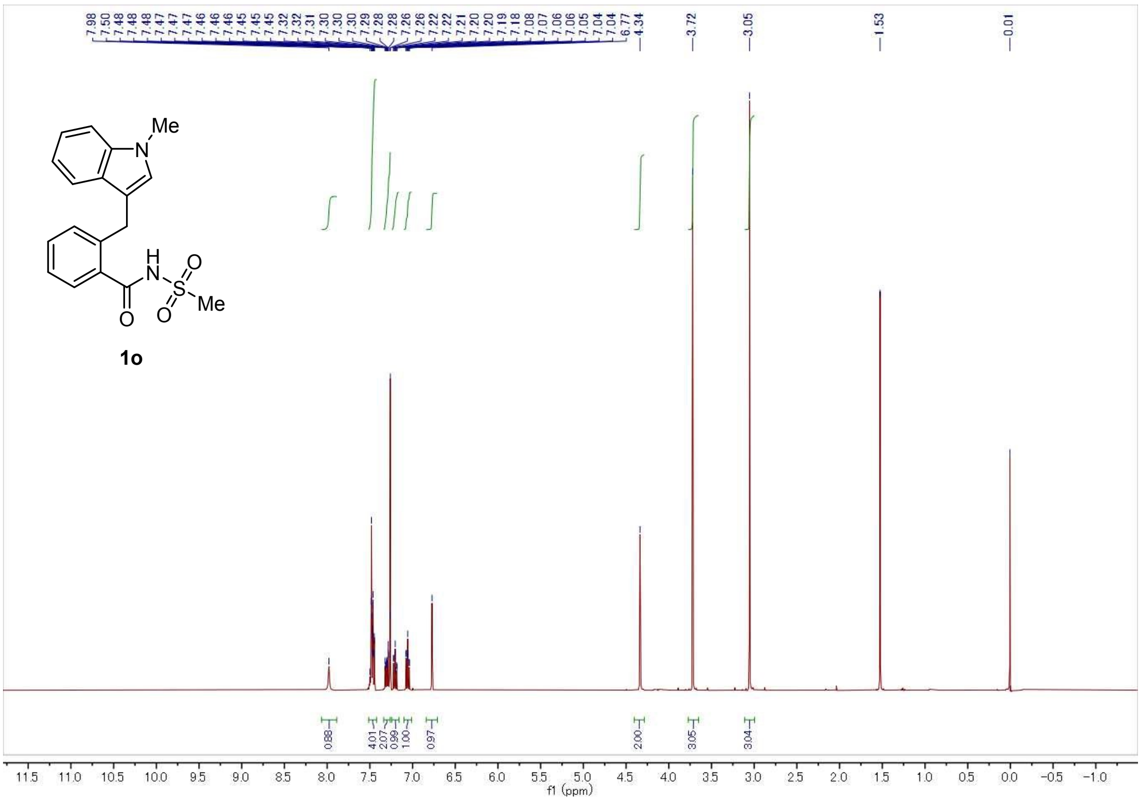

^{13}C NMR (101 MHz, $CDCl_3$) of 1o

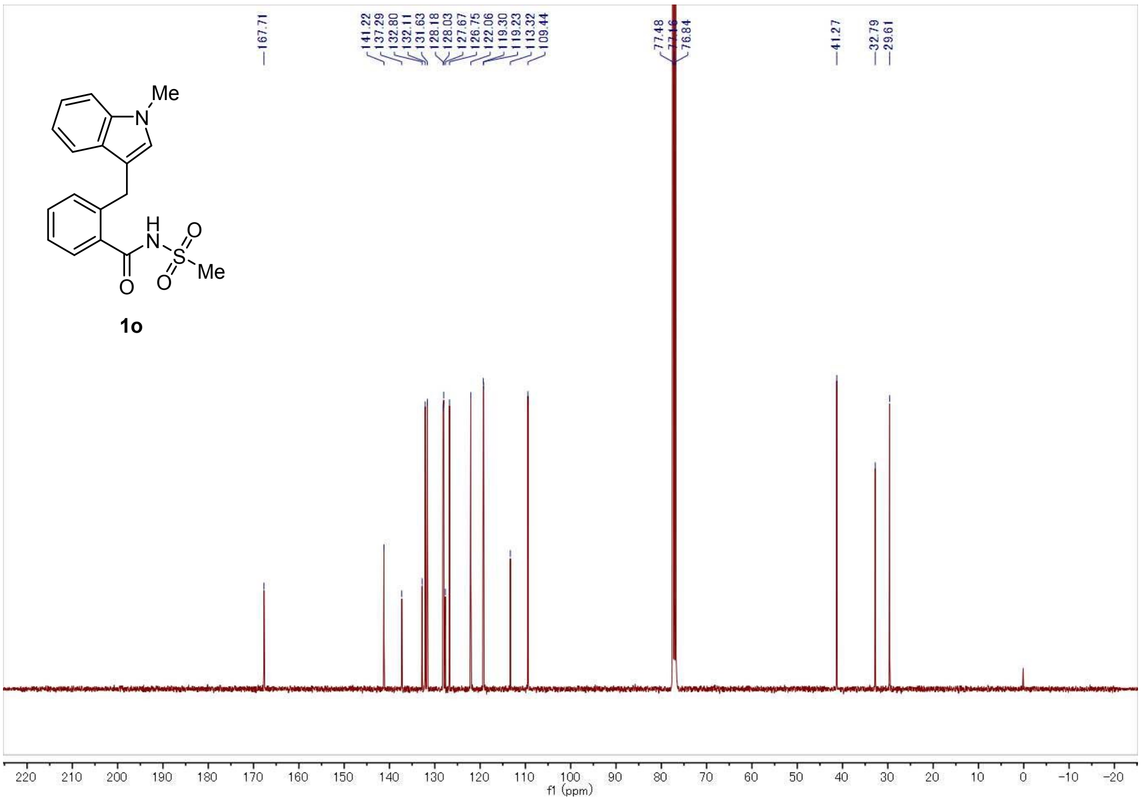

^{1}H NMR (500 MHz, $CDCl_3$) of 1p

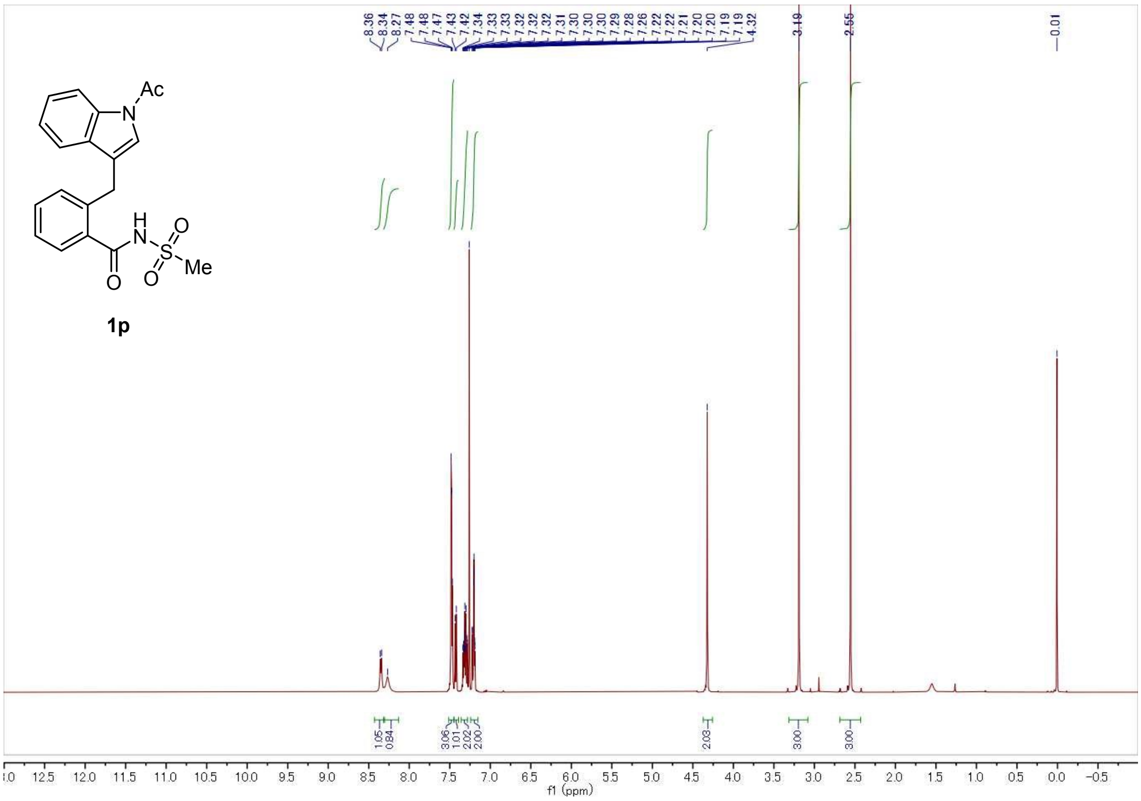

^{13}C NMR (126 MHz, $CDCl_3$) of 1p

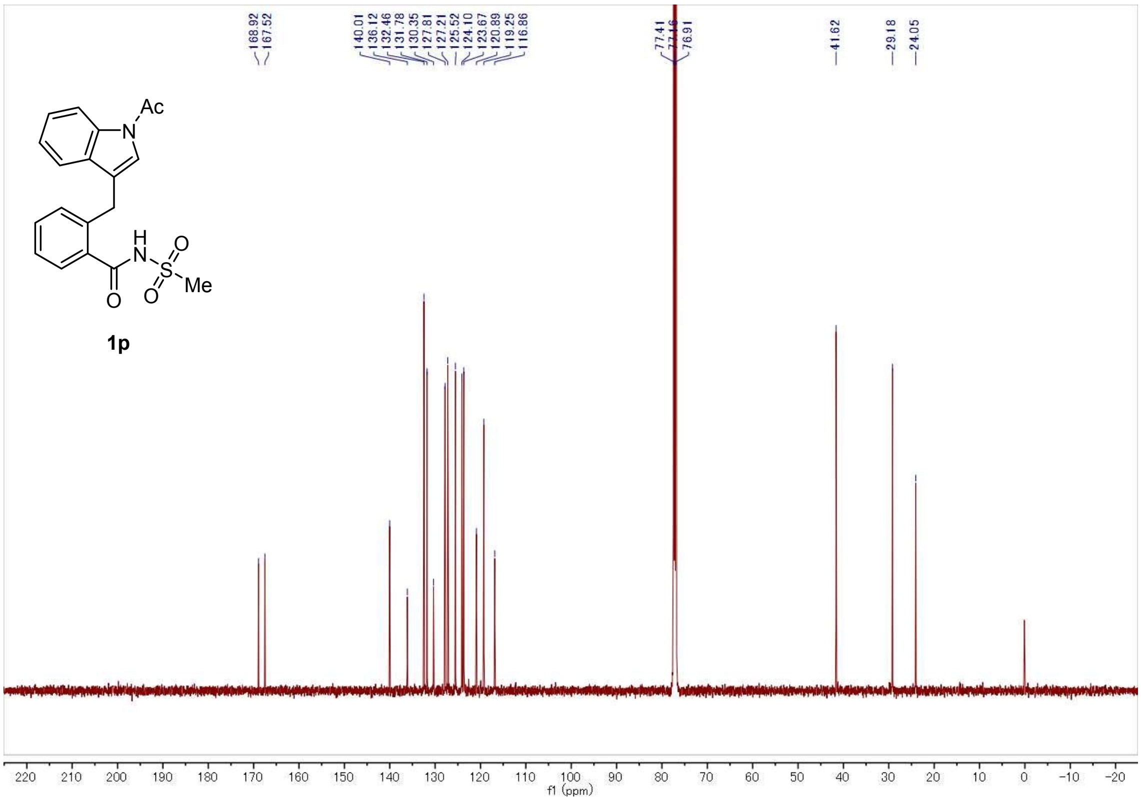

^{1}H NMR (500 MHz, $CDCl_3$) of 1q

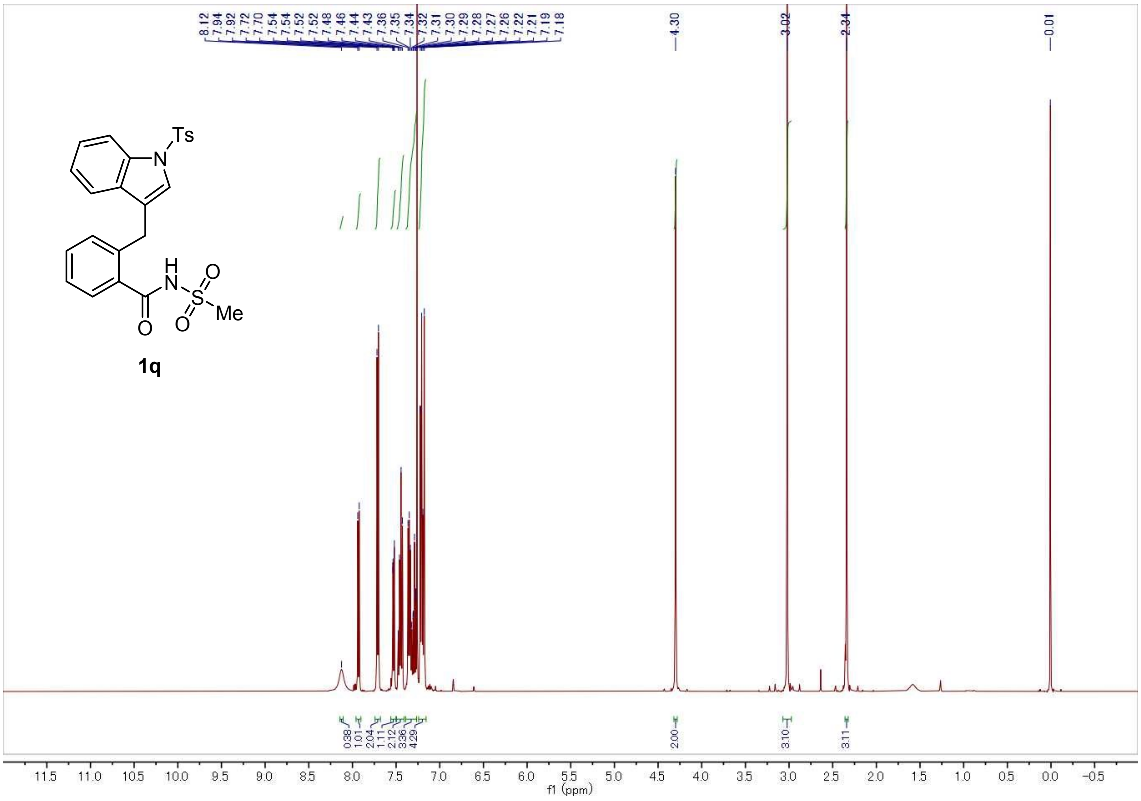

^{13}C NMR (101 MHz, $CDCl_3$) of 1q

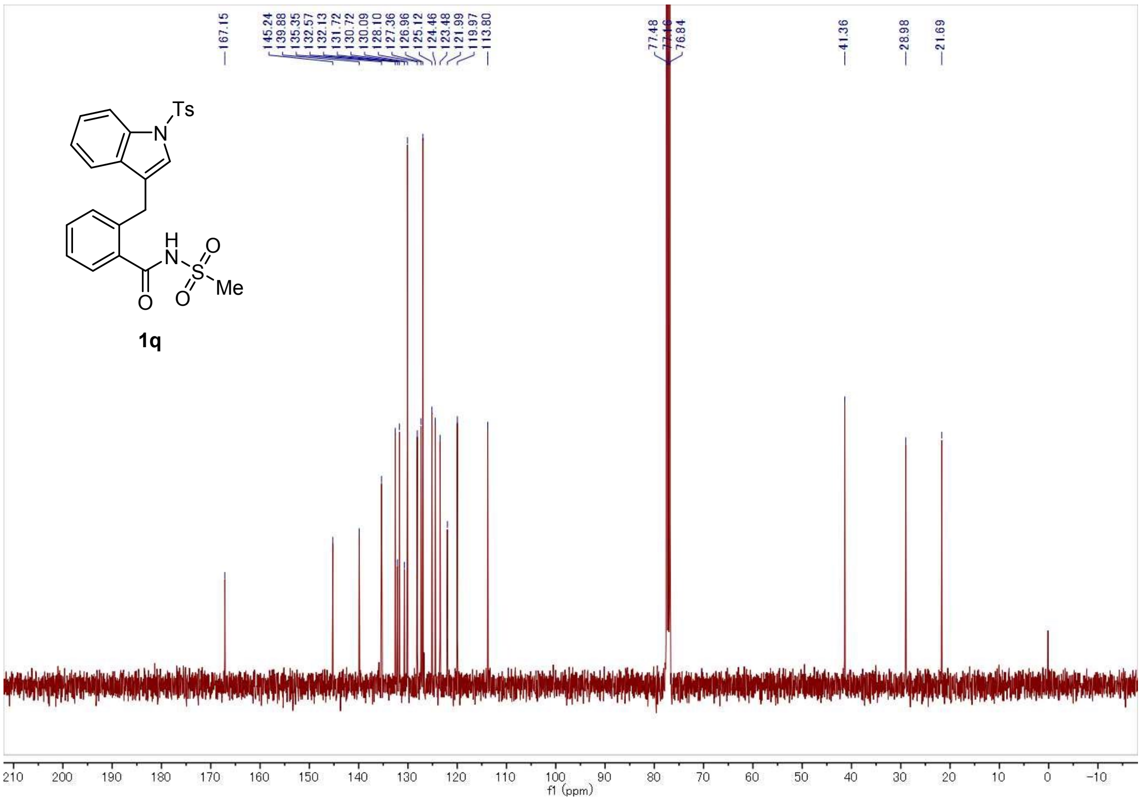

^{1}H NMR (500 MHz, $CDCl_3$) of 1r

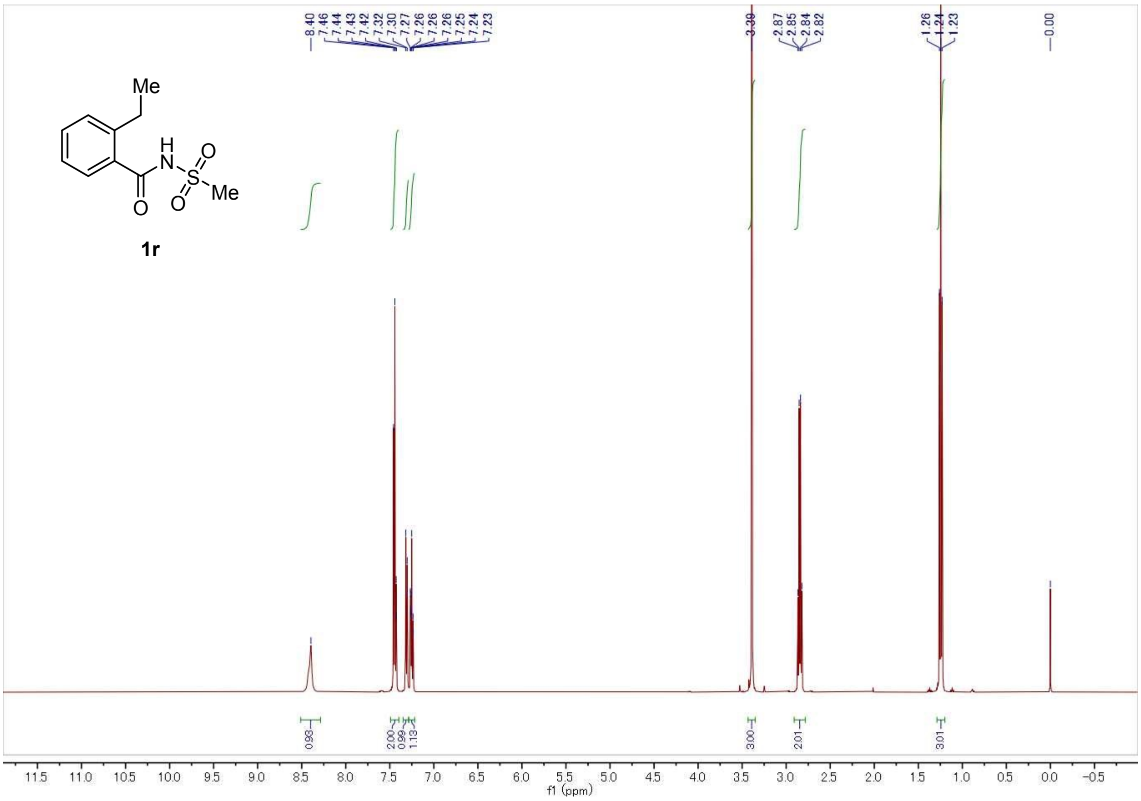

^{13}C NMR (126 MHz, $CDCl_3$) of 1r

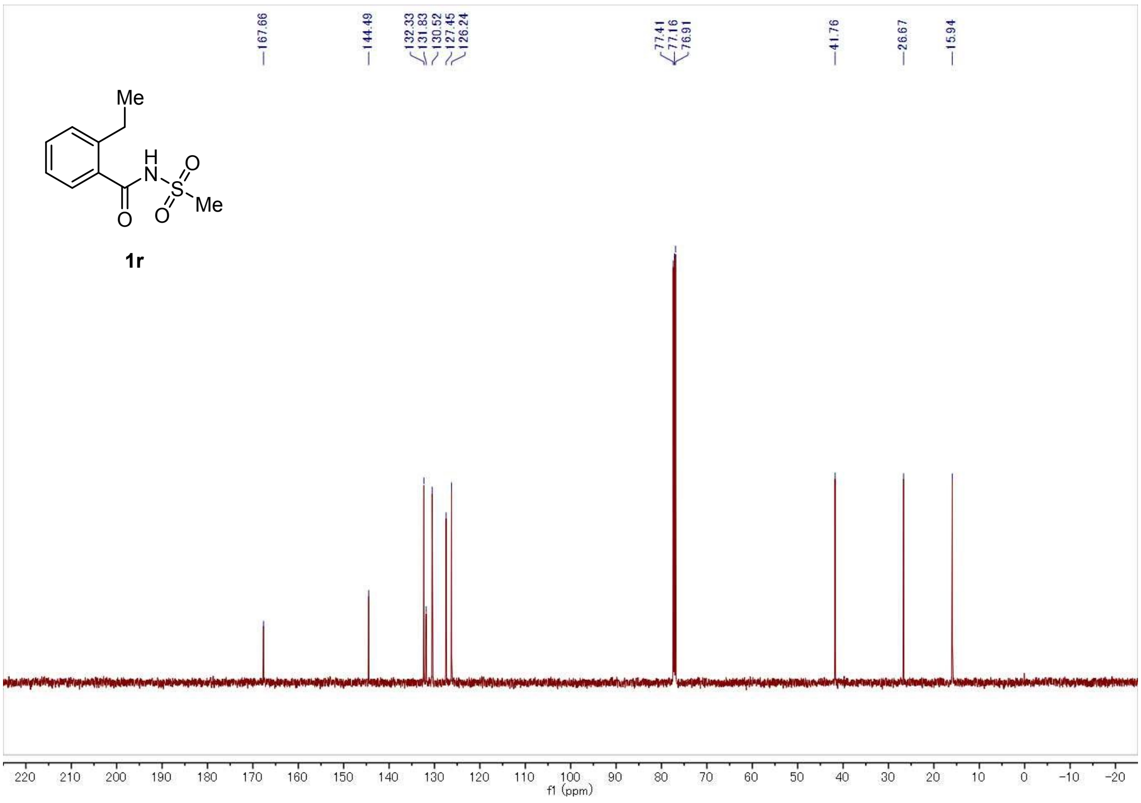

^{1}H NMR (400 MHz, $CDCl_3$) of S1

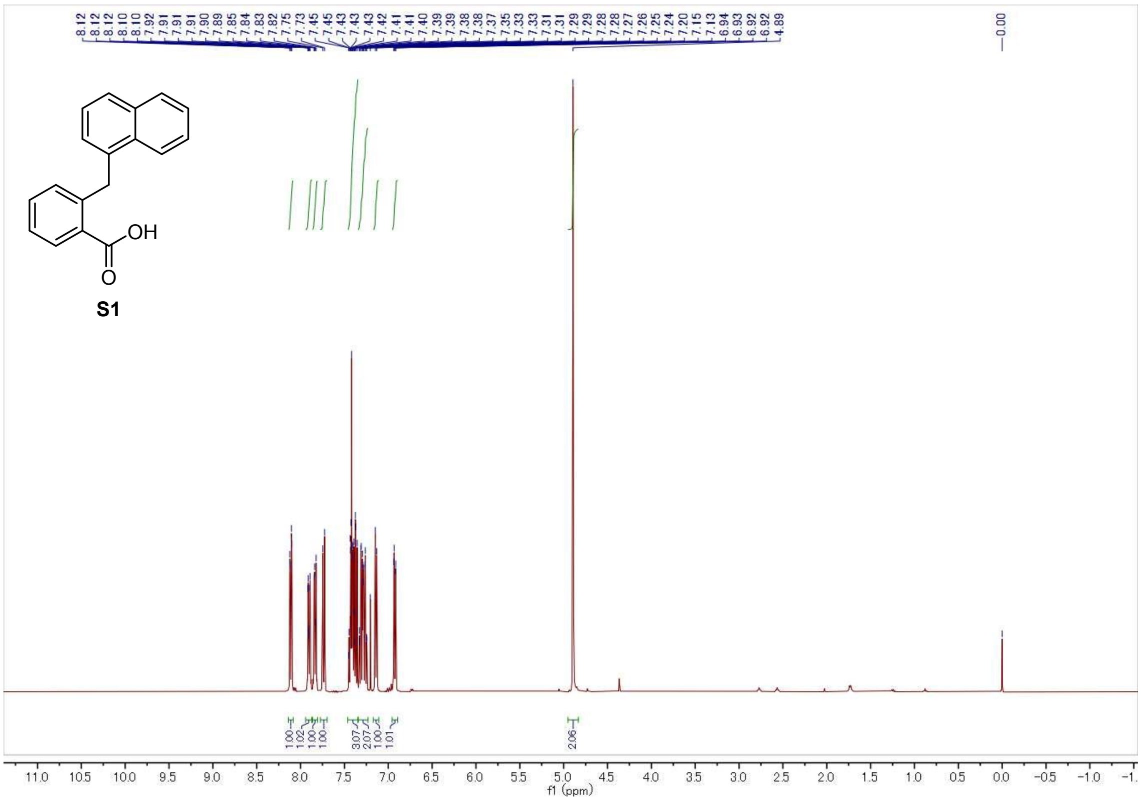

^{13}C NMR (101 MHz, $CDCl_3$) of S1

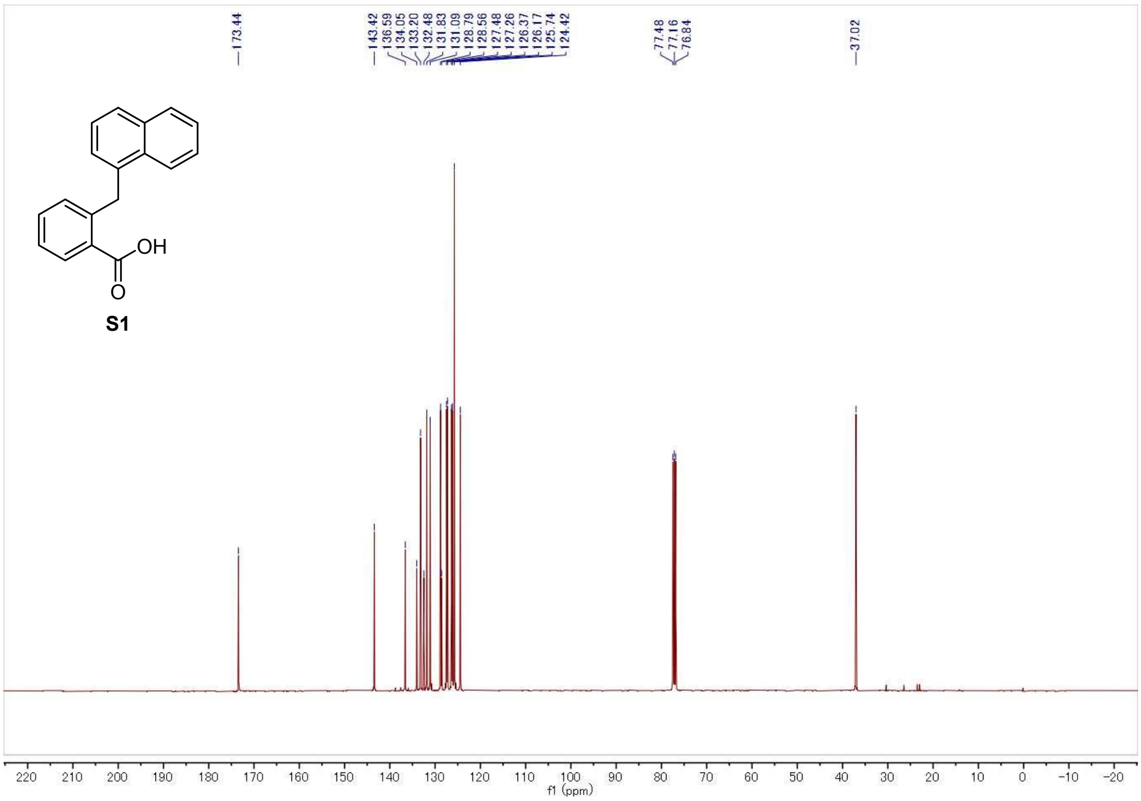

^{1}H NMR (400 MHz, $CDCl_3$) of S2

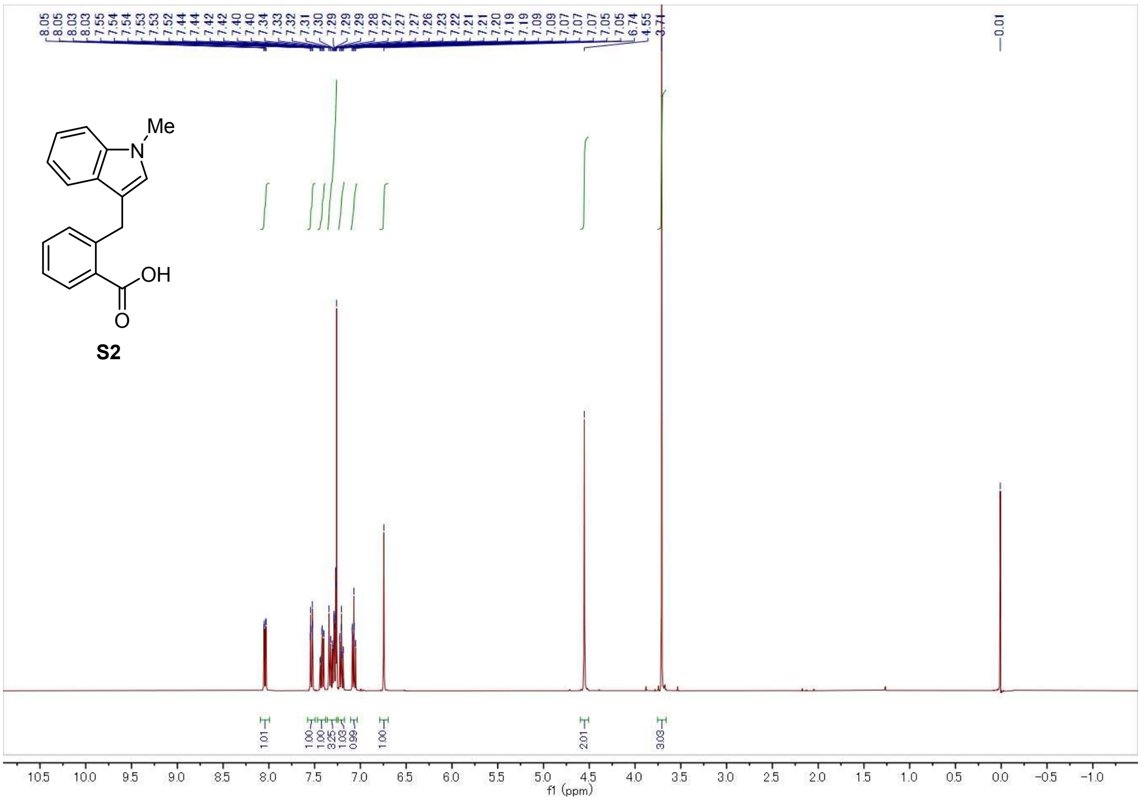

^{13}C NMR (101 MHz, $CDCl_3$) of S2

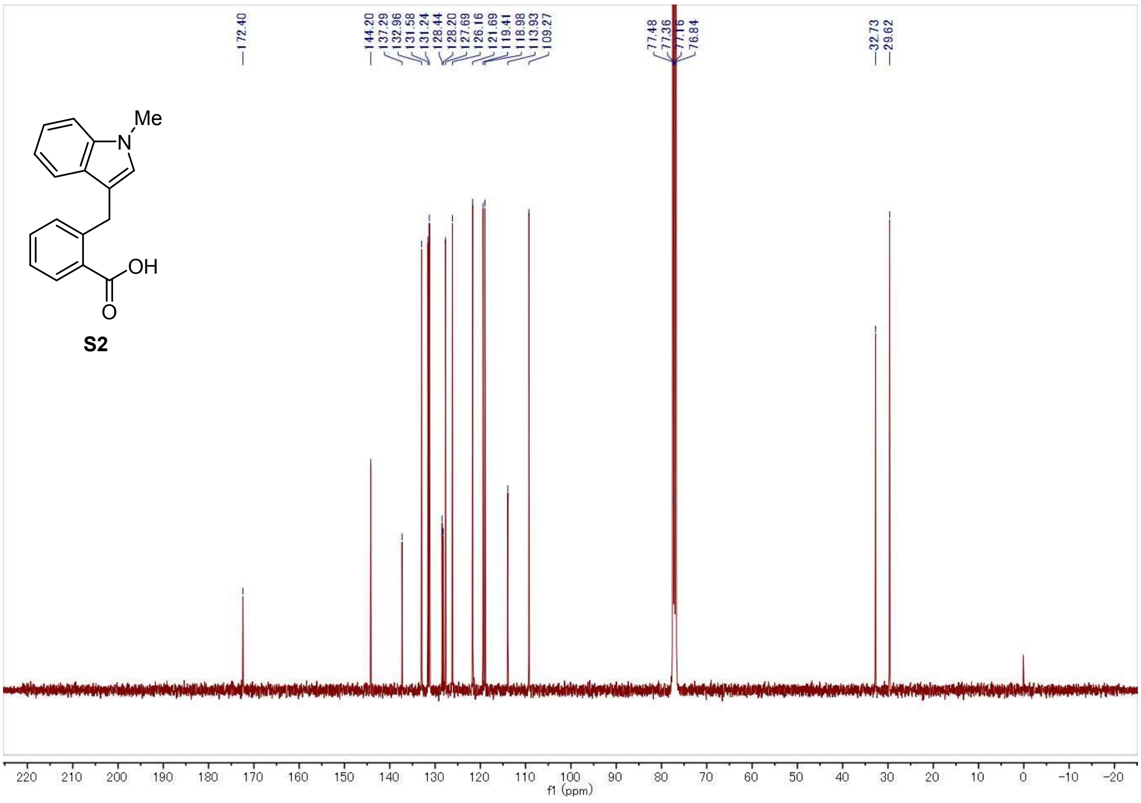

^{1}H NMR (400 MHz, DMSO-d_6) of S3

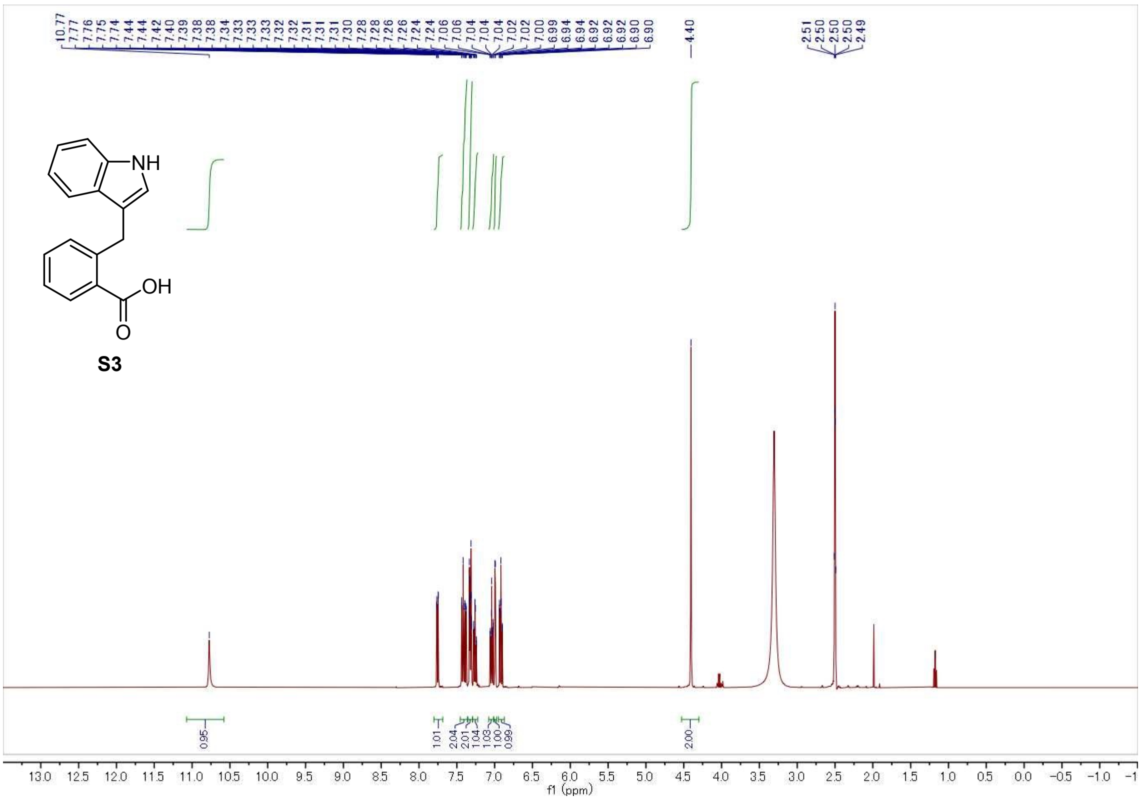

^{13}C NMR (101 MHz, DMSO-d_6) of S3

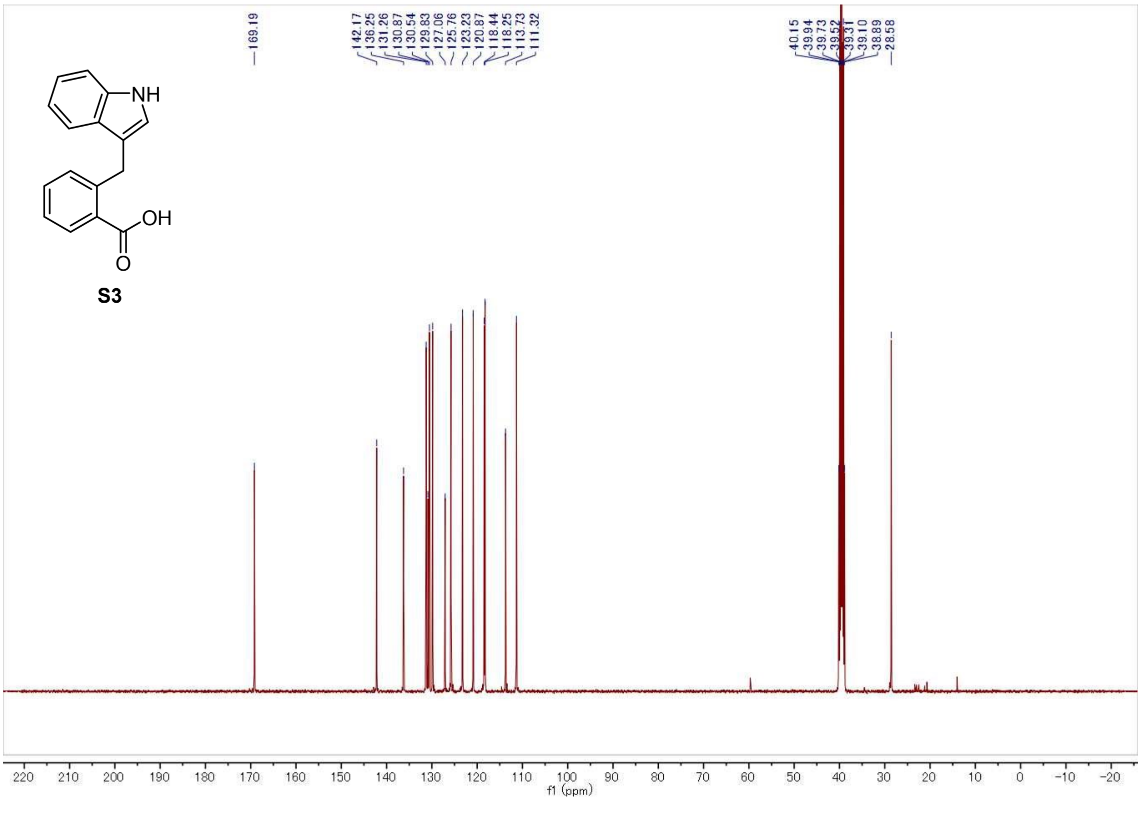

^{1}H NMR (400 MHz, acetone-d_6) of S4

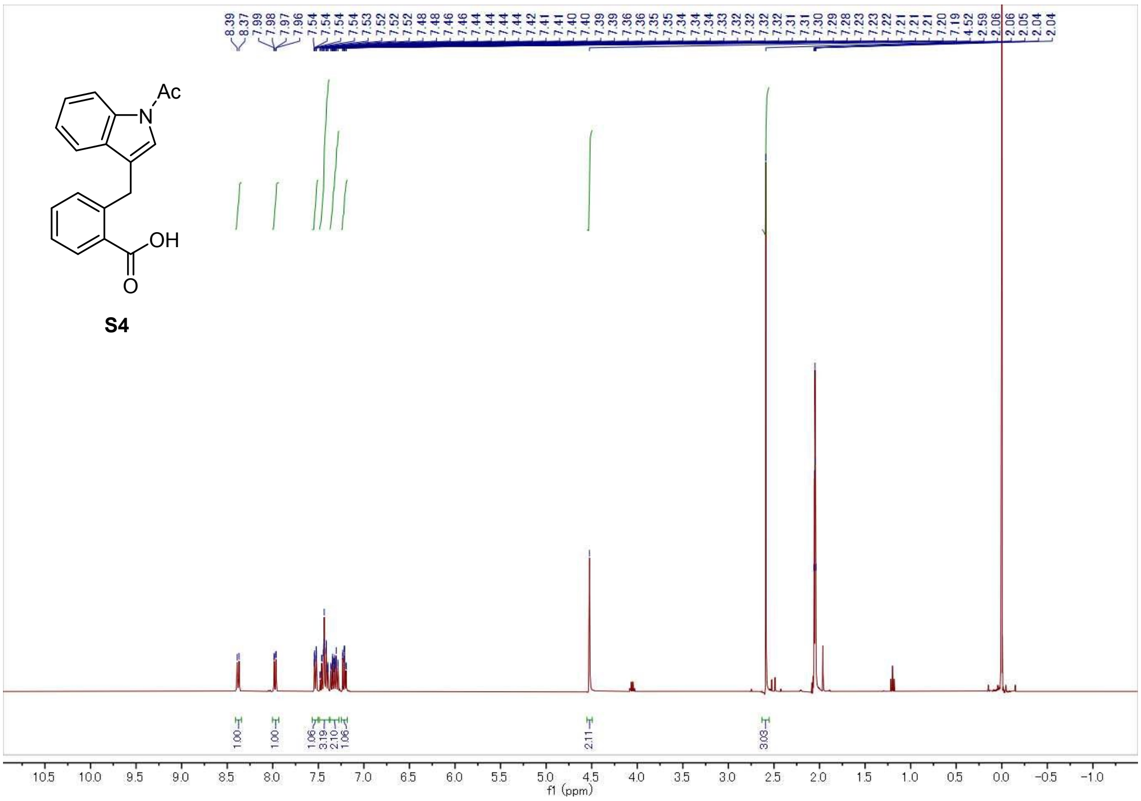

^{13}C NMR (101 MHz, acetone-d_6) of S4

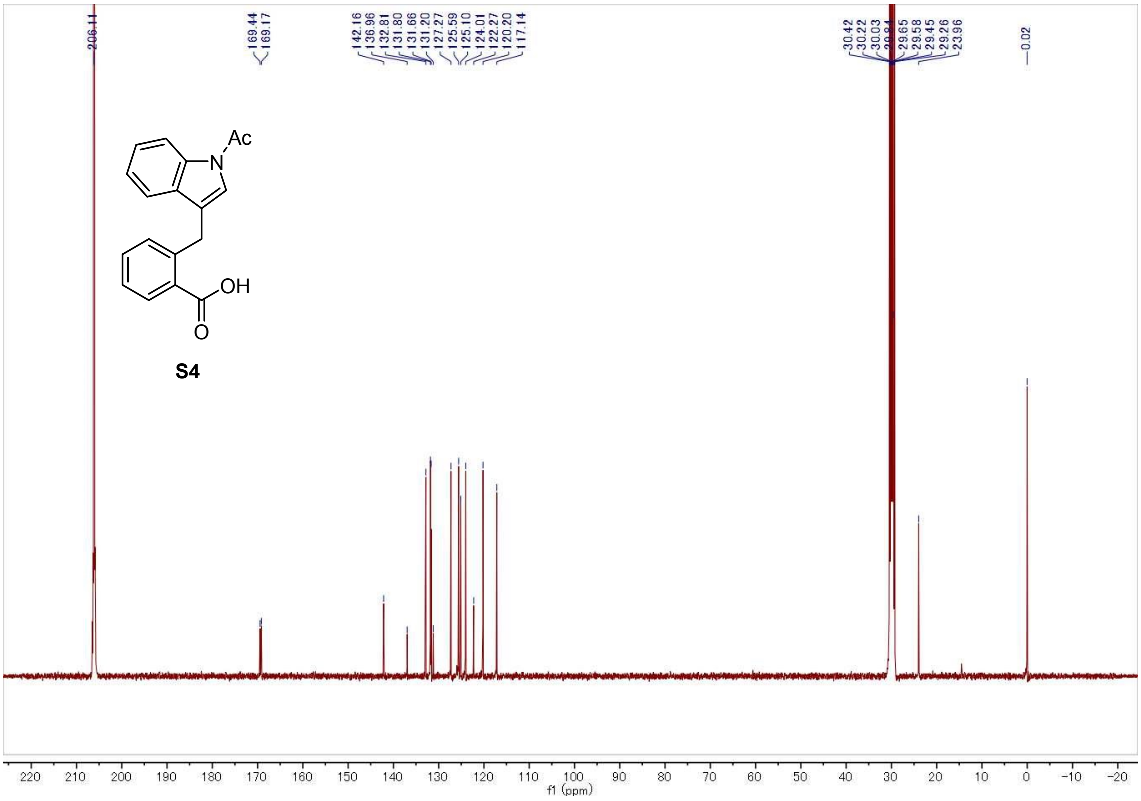

^{1}H NMR (400 MHz, acetone-d_6) of S5

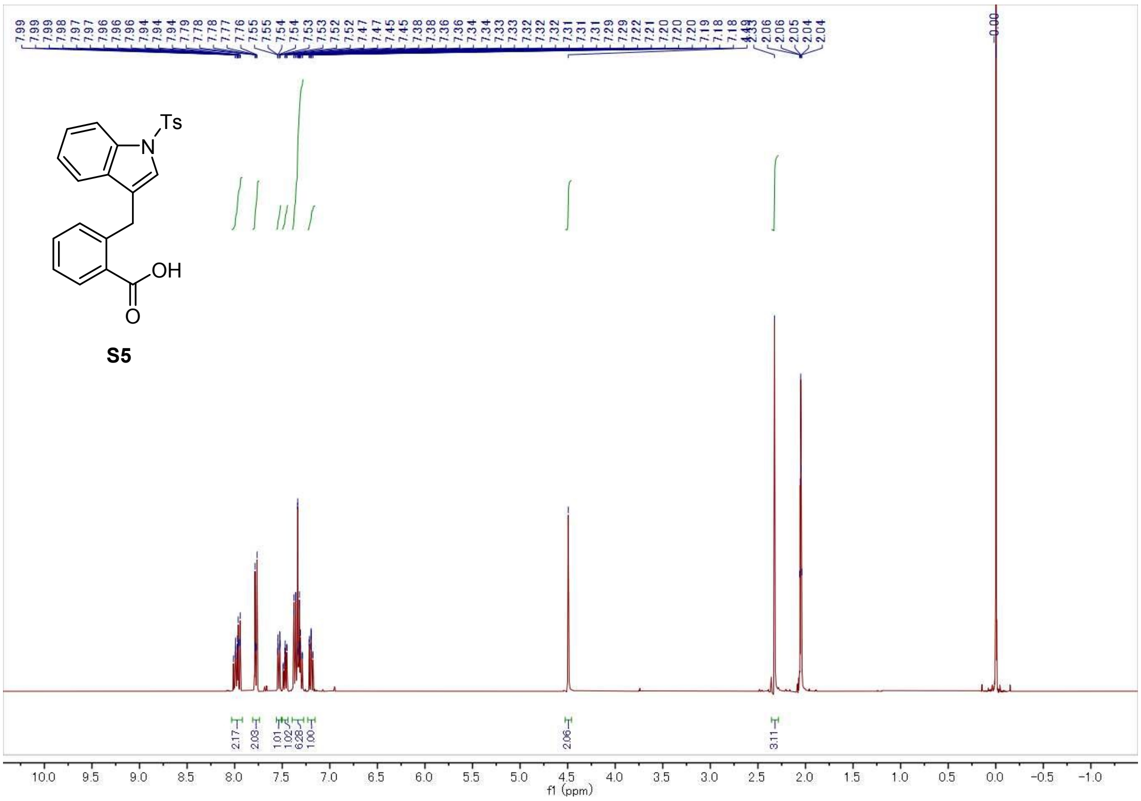

^{13}C NMR (101 MHz, acetone-d_6) of S5

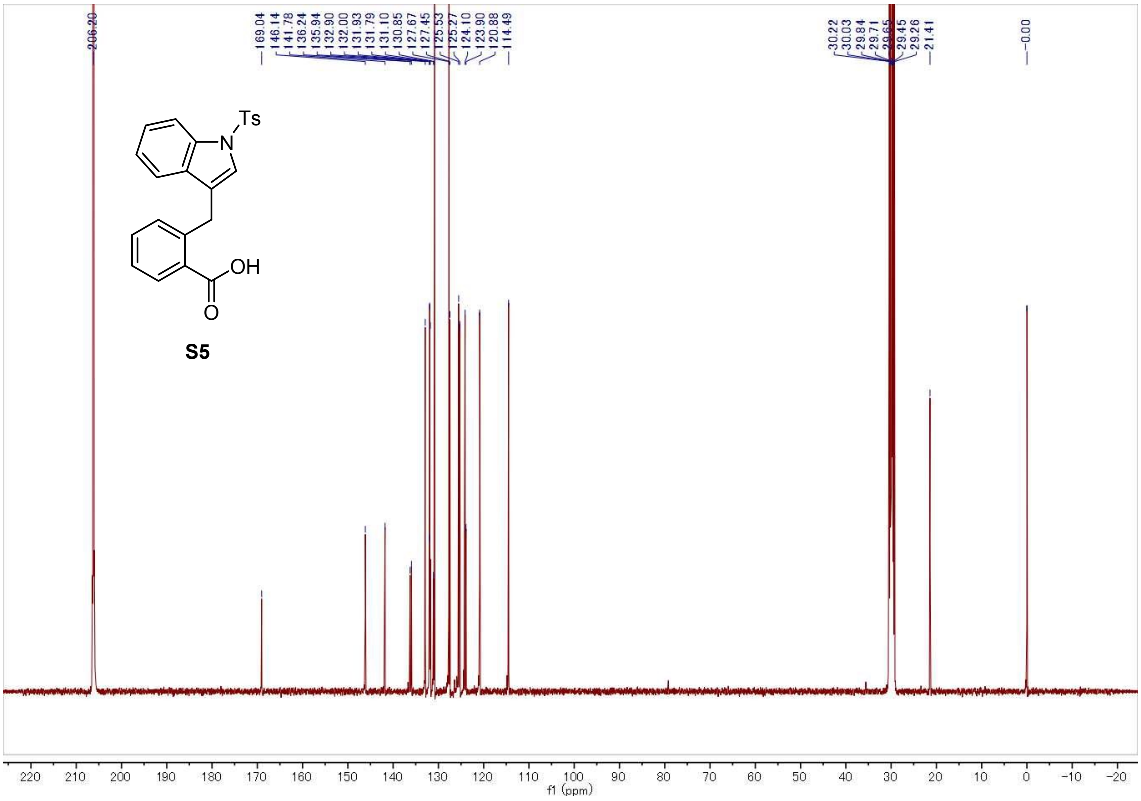

^{1}H NMR (400 MHz, $CDCl_3$) of 2a

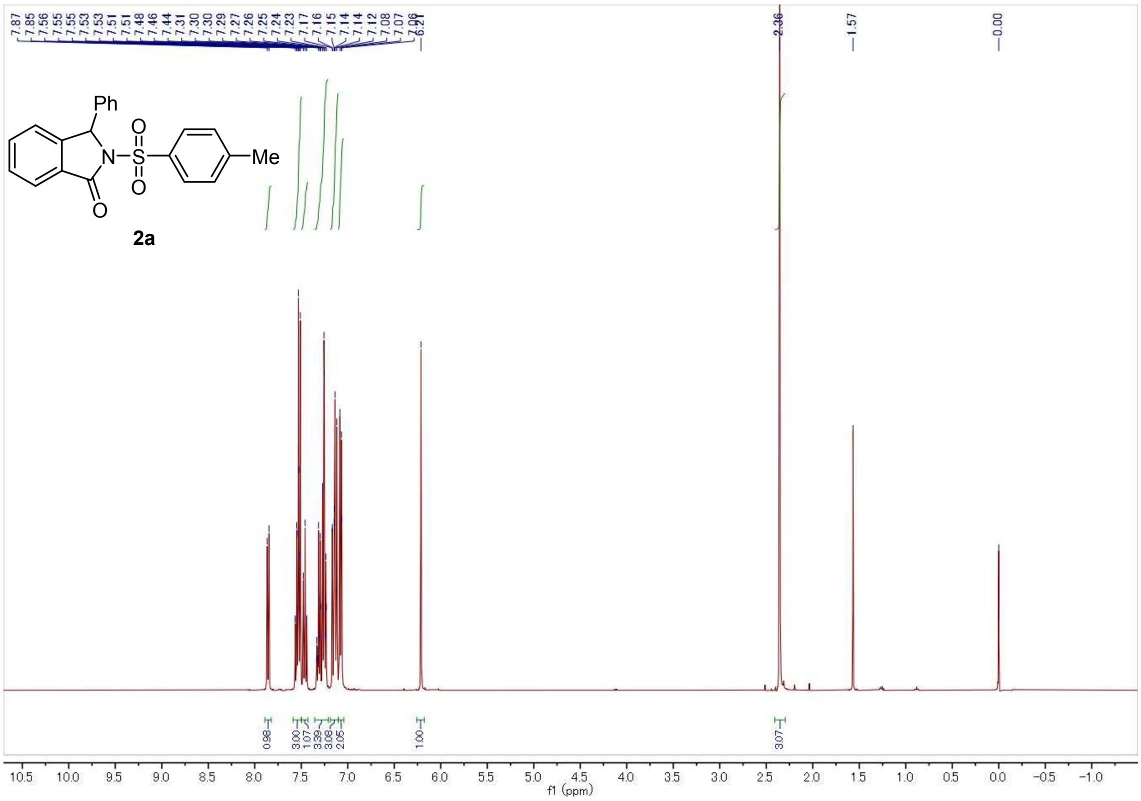

^{13}C NMR (101 MHz, $CDCl_3$) of 2a

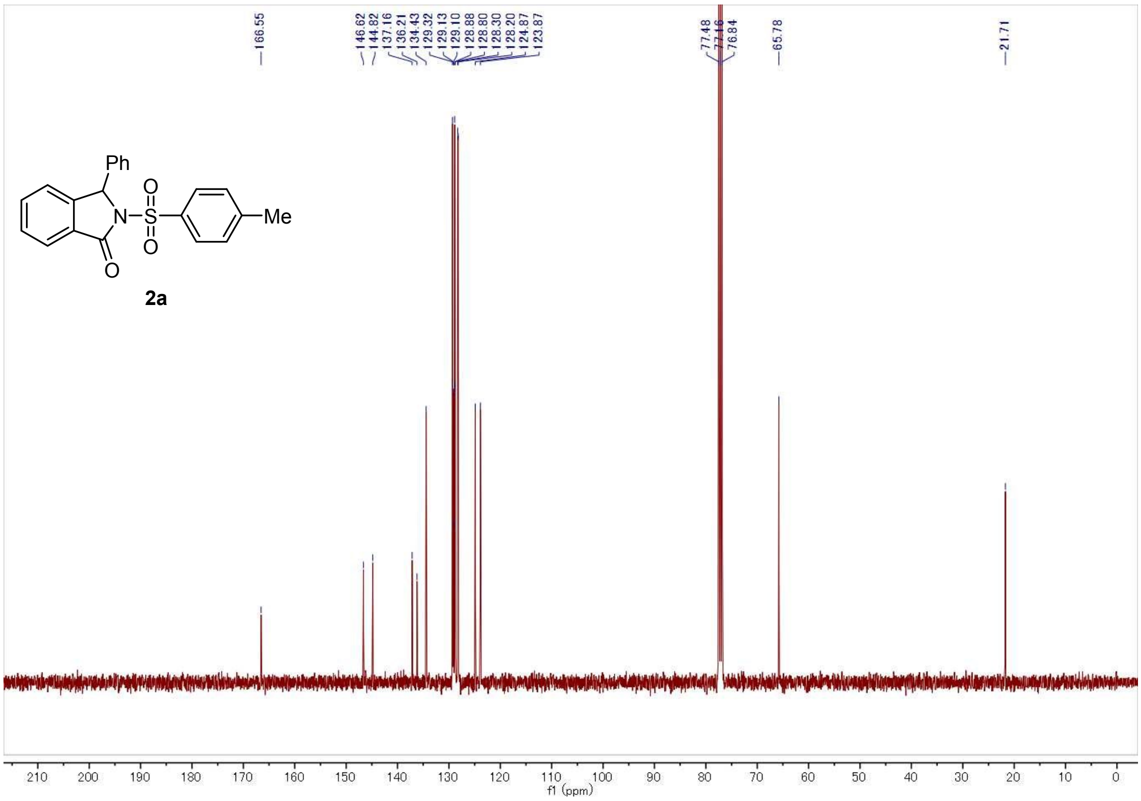

^{1}H NMR (500 MHz, $CDCl_3$) of 2b

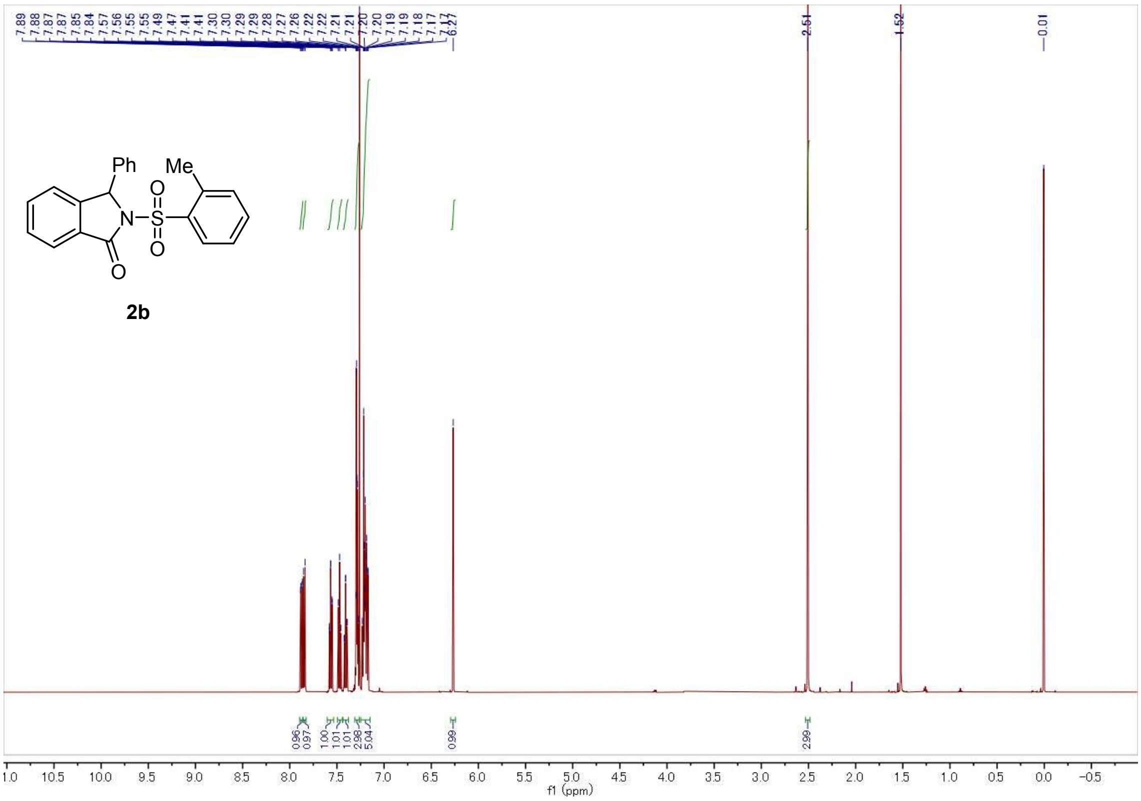

^{13}C NMR (126 MHz, $CDCl_3$) of 2b

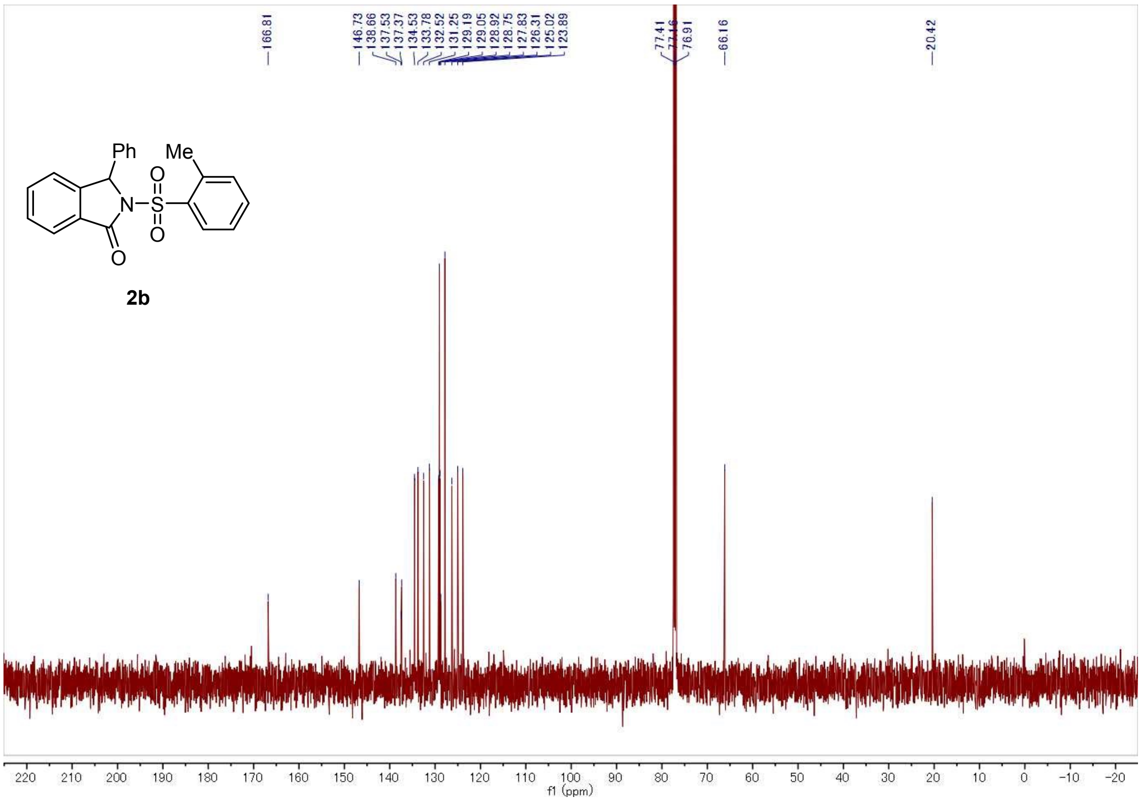

^{1}H NMR (500 MHz, $CDCl_3$) of 2c

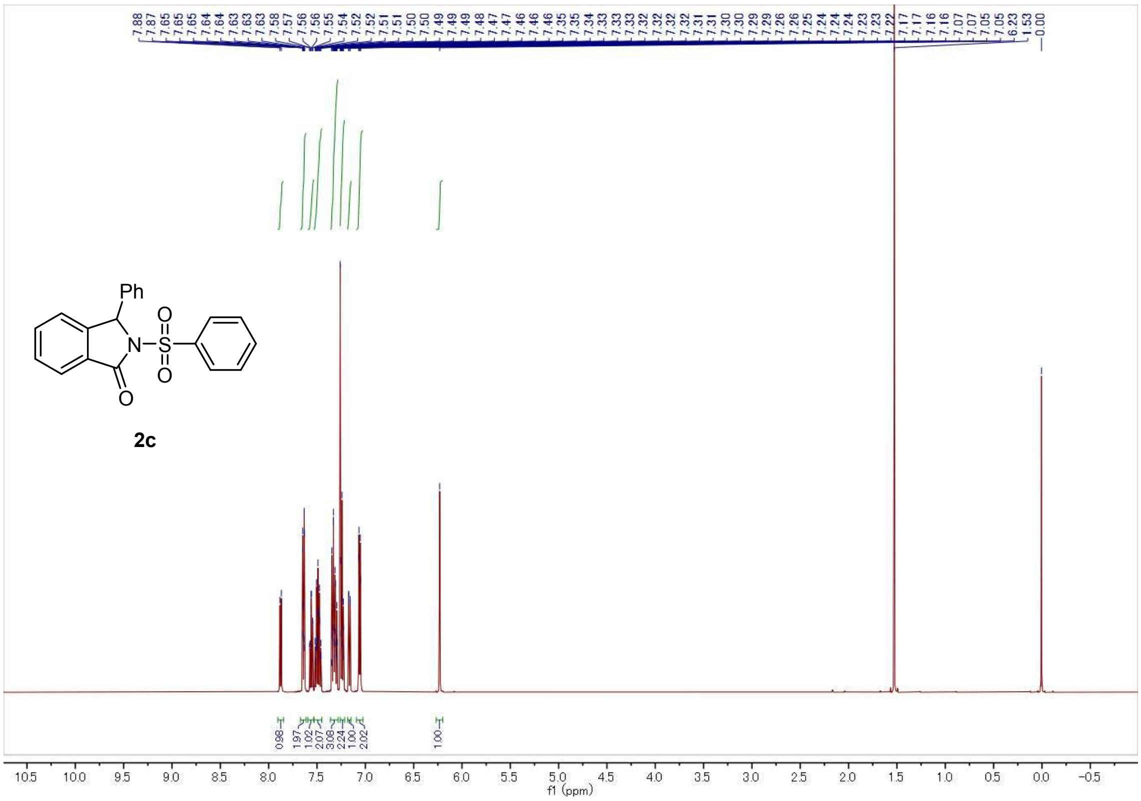

^{13}C NMR (126 MHz, $CDCl_3$) of 2c

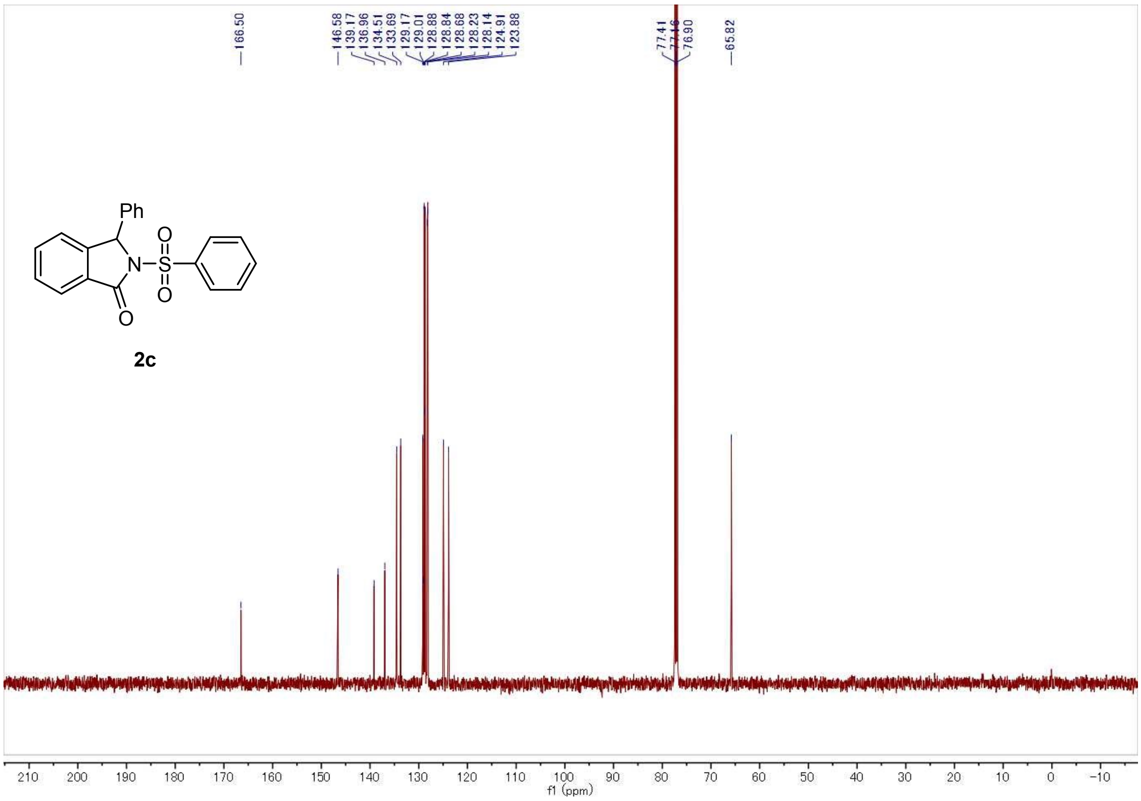

^{1}H NMR (500 MHz, $CDCl_3$) of 2d

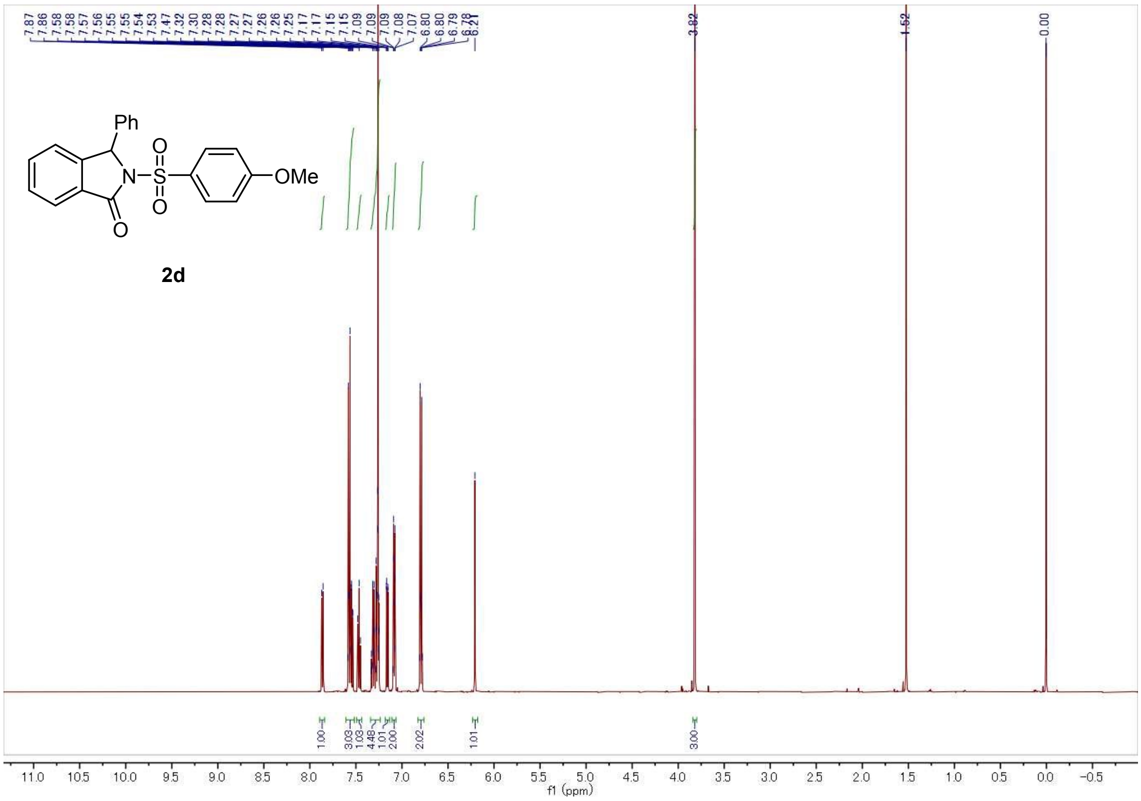

^{13}C NMR (126 MHz, $CDCl_3$) of 2d

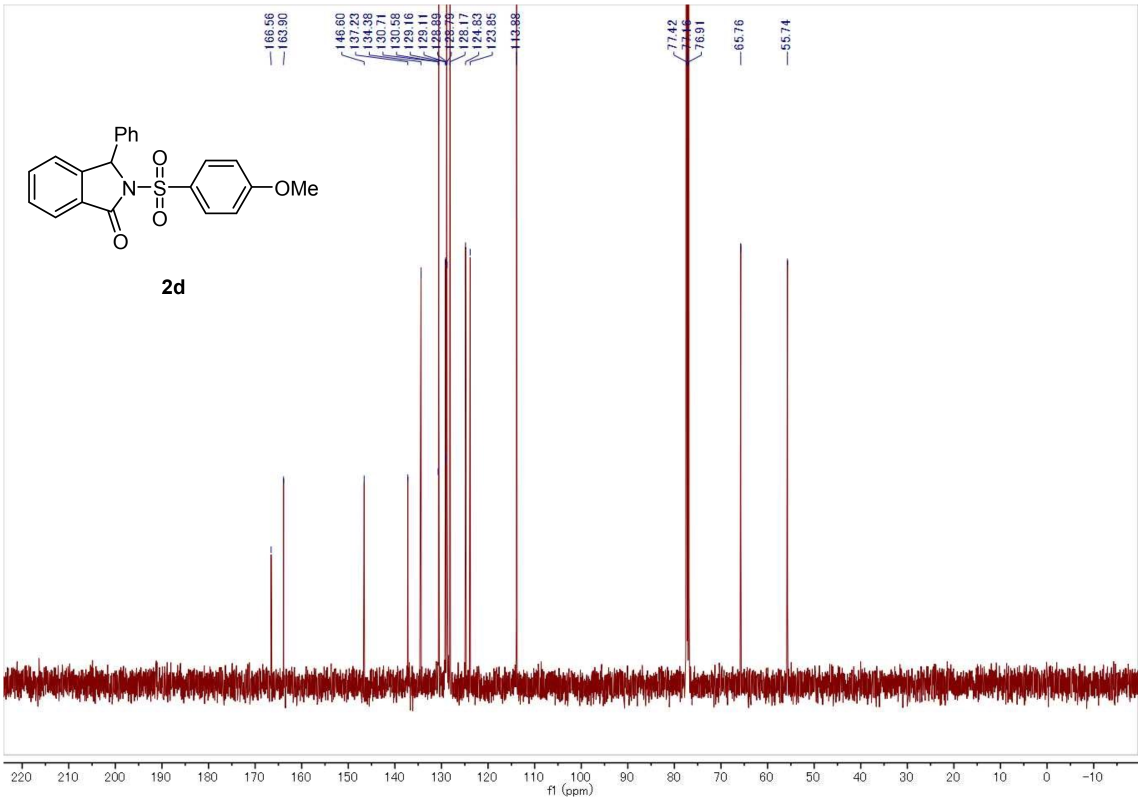

^{1}H NMR (500 MHz, CDCl$_3$) of 2f

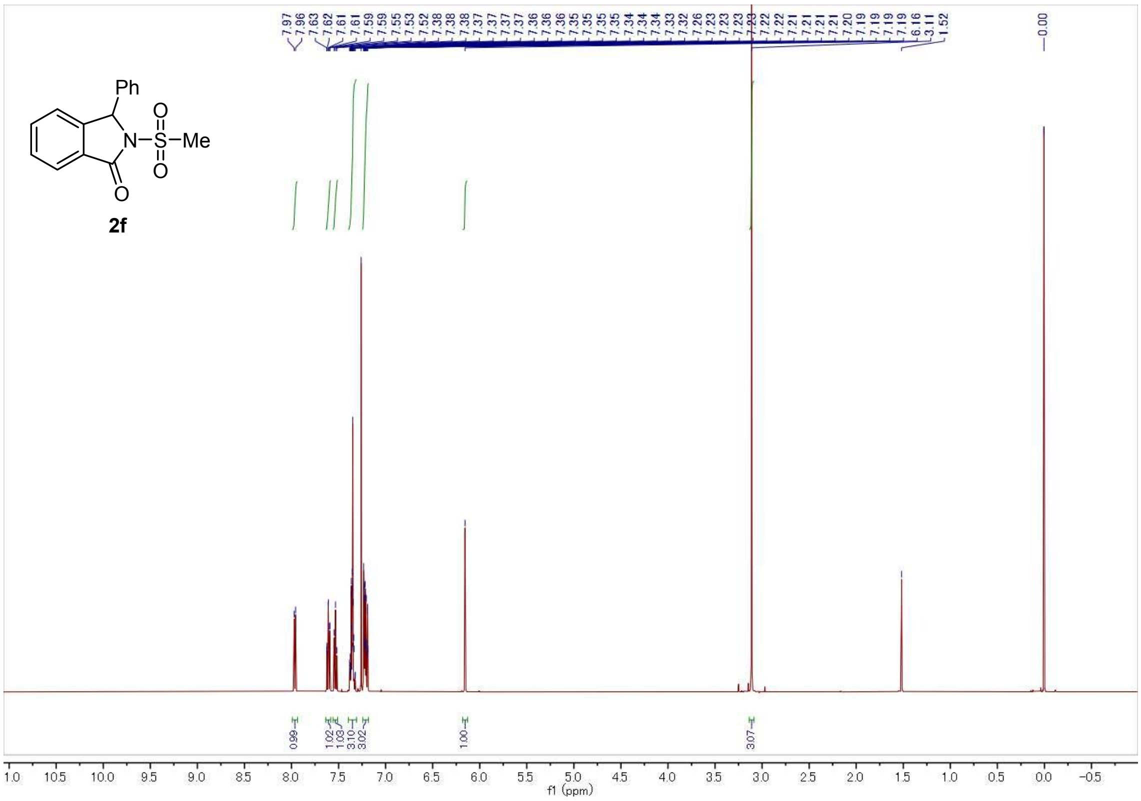

^{13}C NMR (126 MHz, CDCl$_3$) of 2f

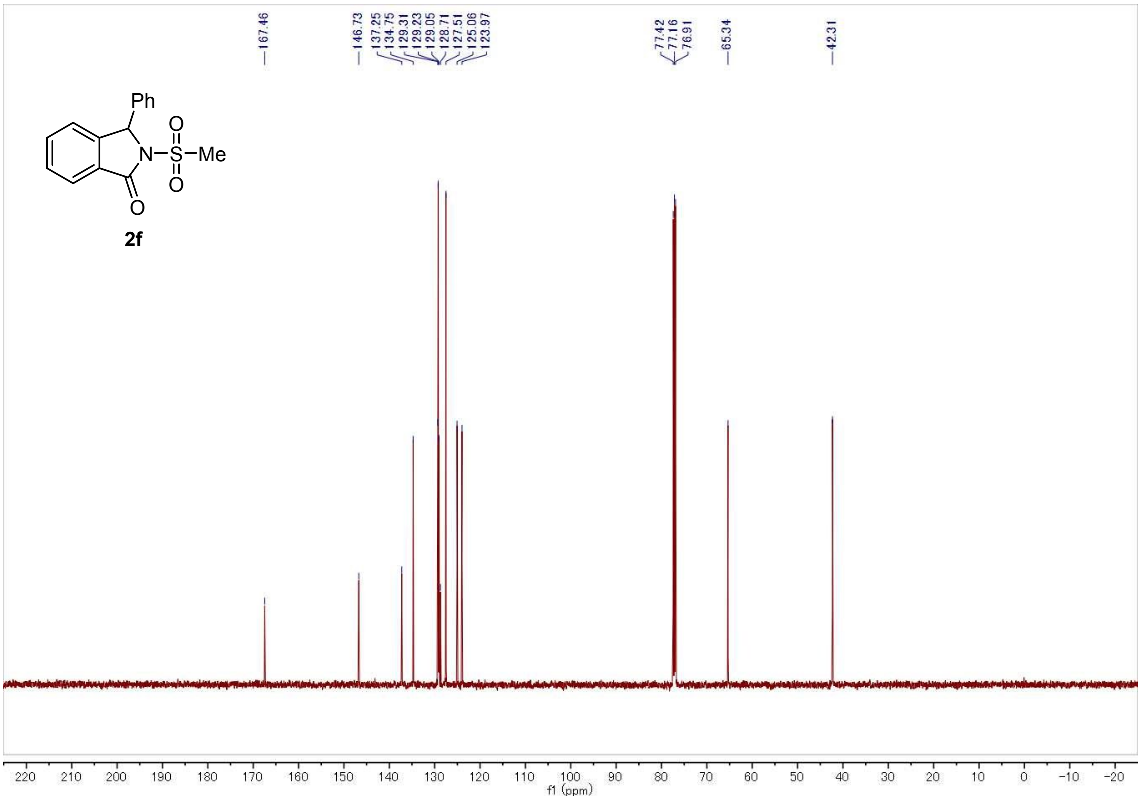

^{1}H NMR (500 MHz, $CDCl_3$) of 2g

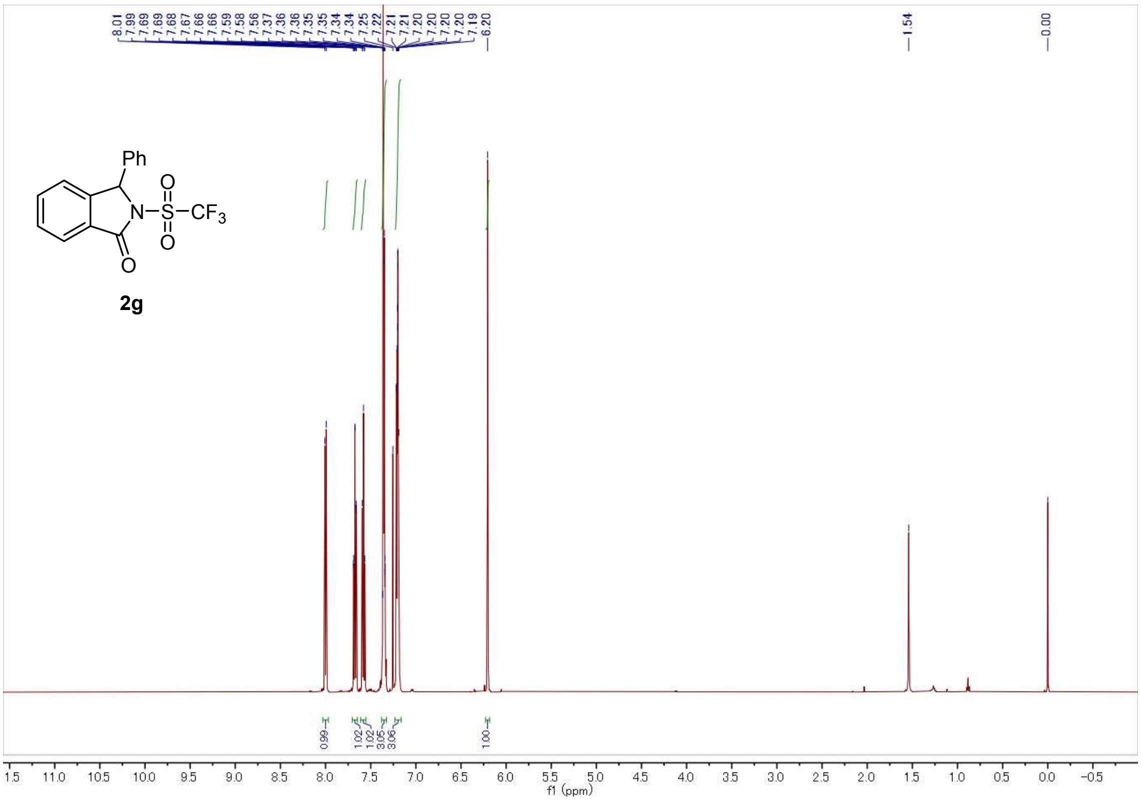

^{13}C NMR (126 MHz, $CDCl_3$) of 2g

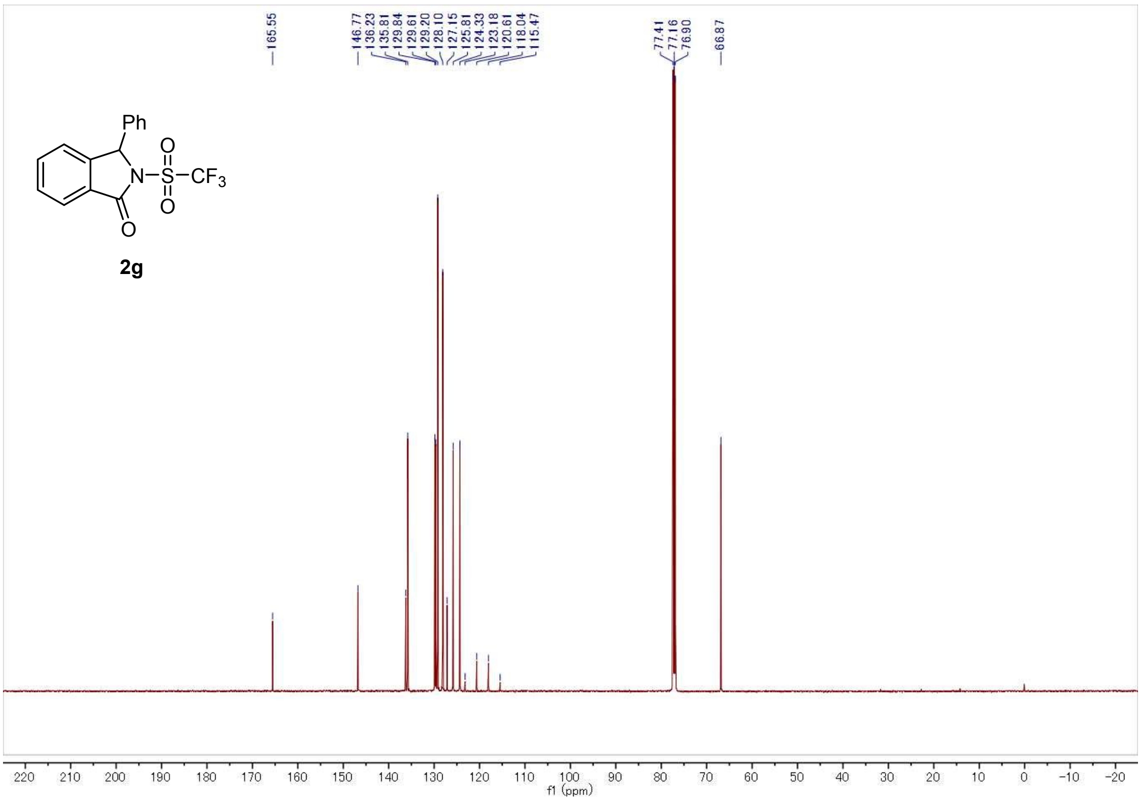

^{19}F NMR (376 MHz, CDCl$_3$) of 2g

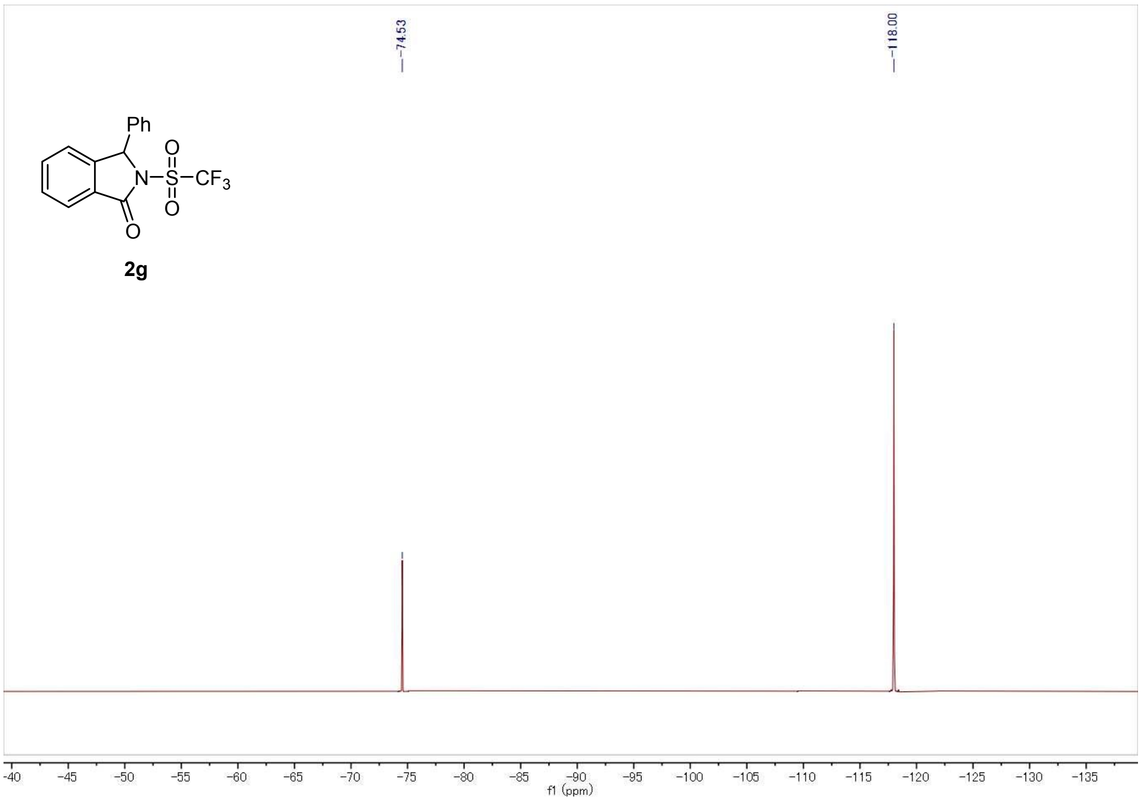

^{1}H NMR (500 MHz, $CDCl_3$) of 2j

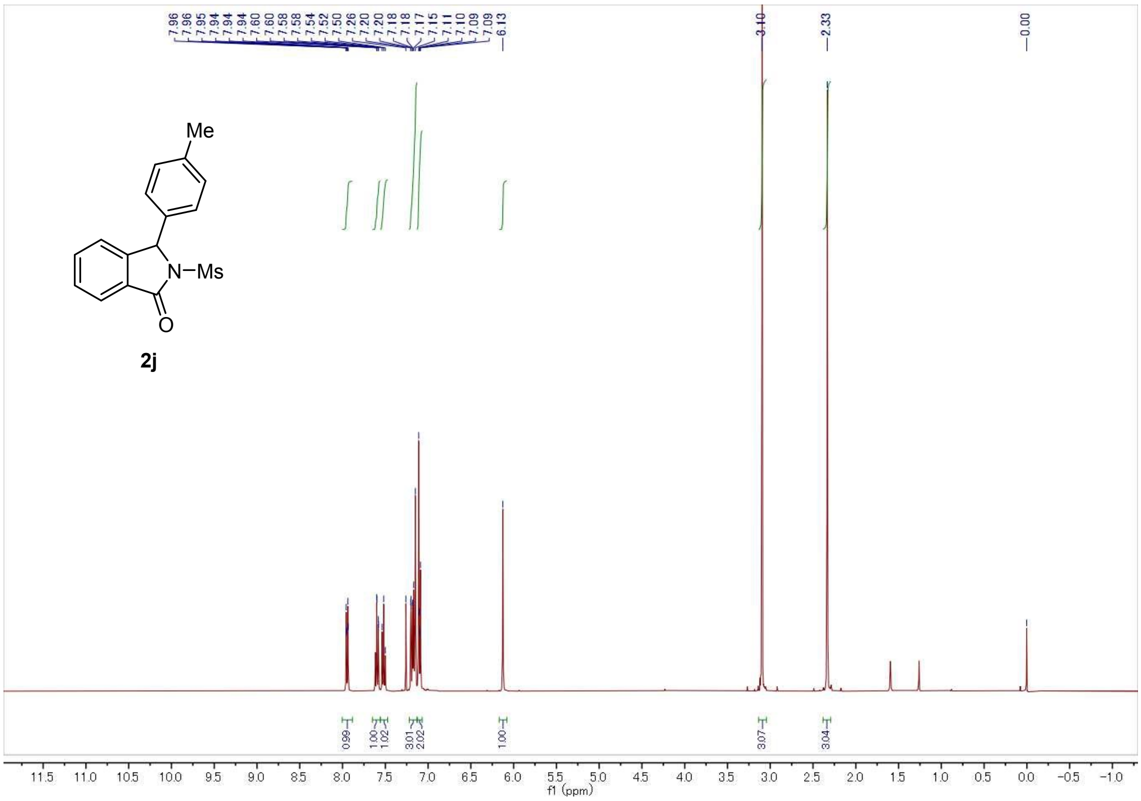

^{13}C NMR (126 MHz, $CDCl_3$) of 2j

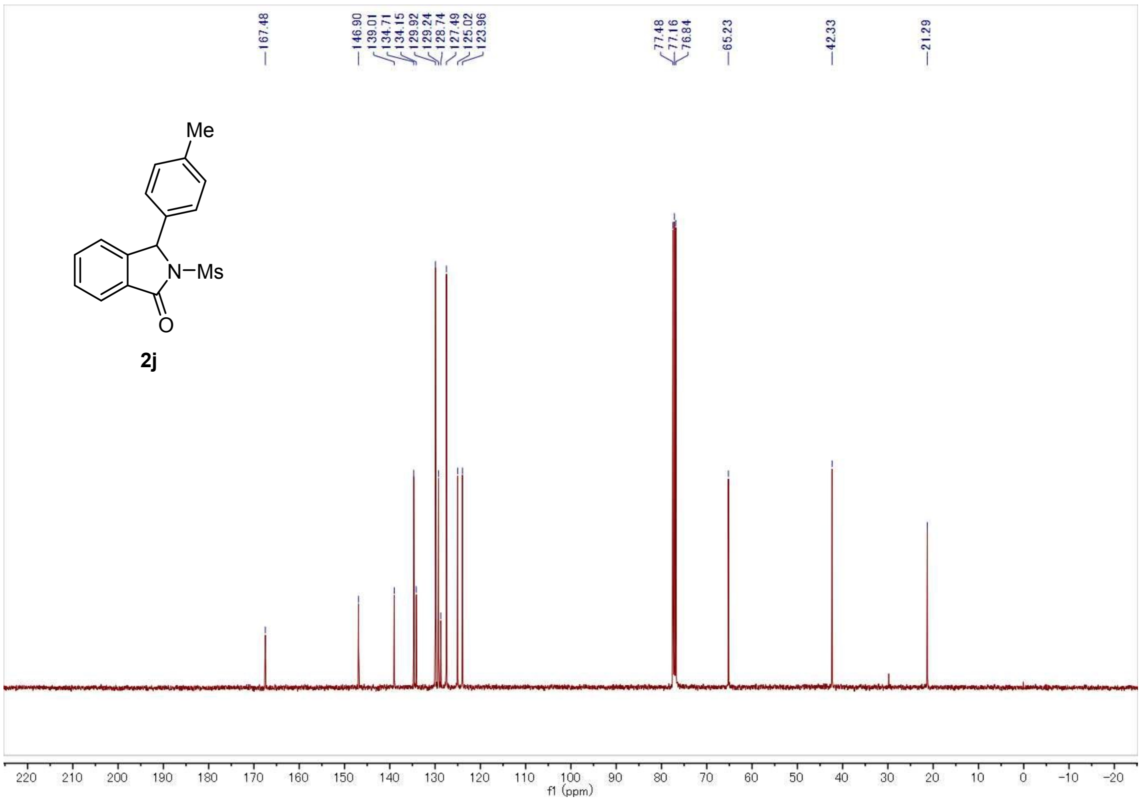

^{1}H NMR (400 MHz, $CDCl_3$) of 2k

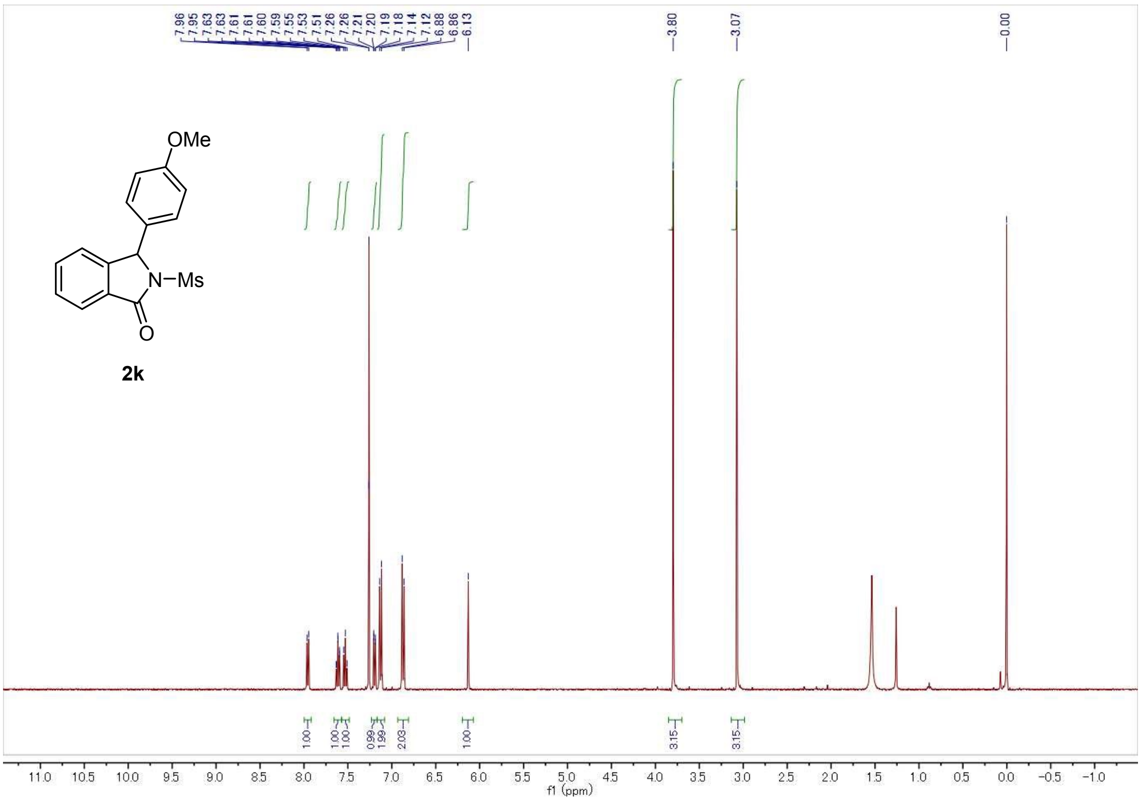

^{13}C NMR (101 MHz, $CDCl_3$) of 2k

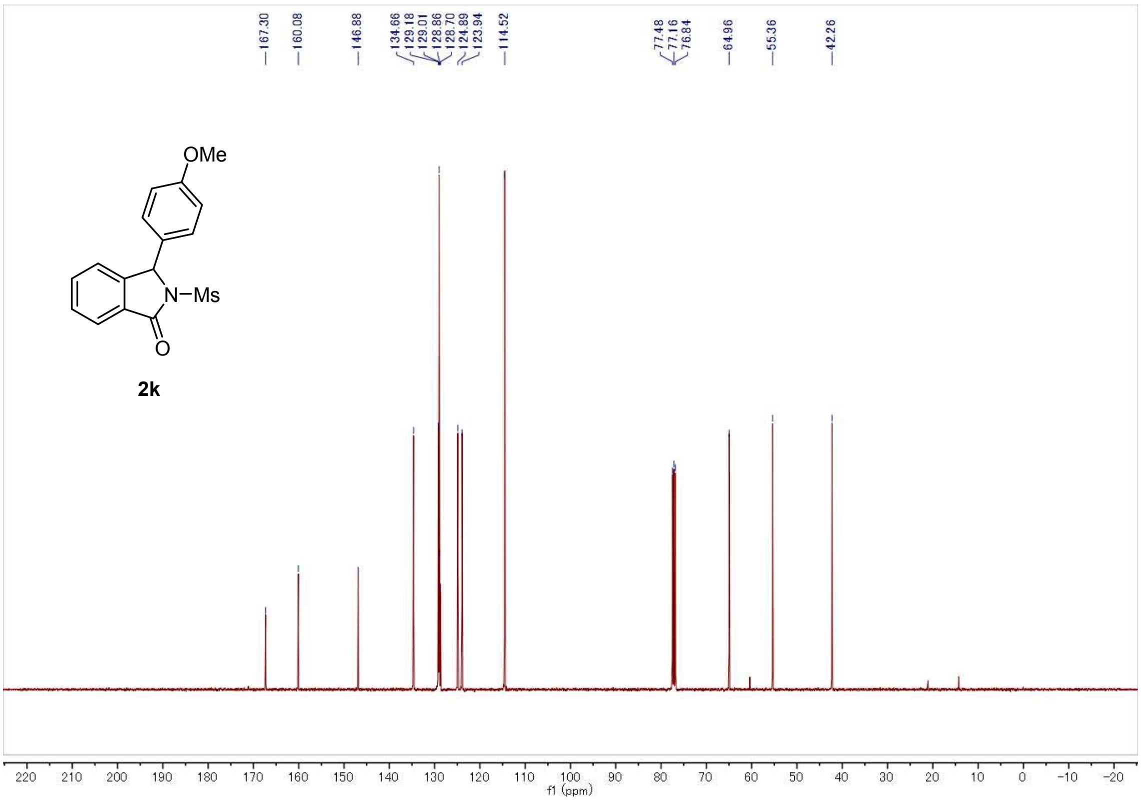

^{1}H NMR (500 MHz, $CDCl_3$) of 2l

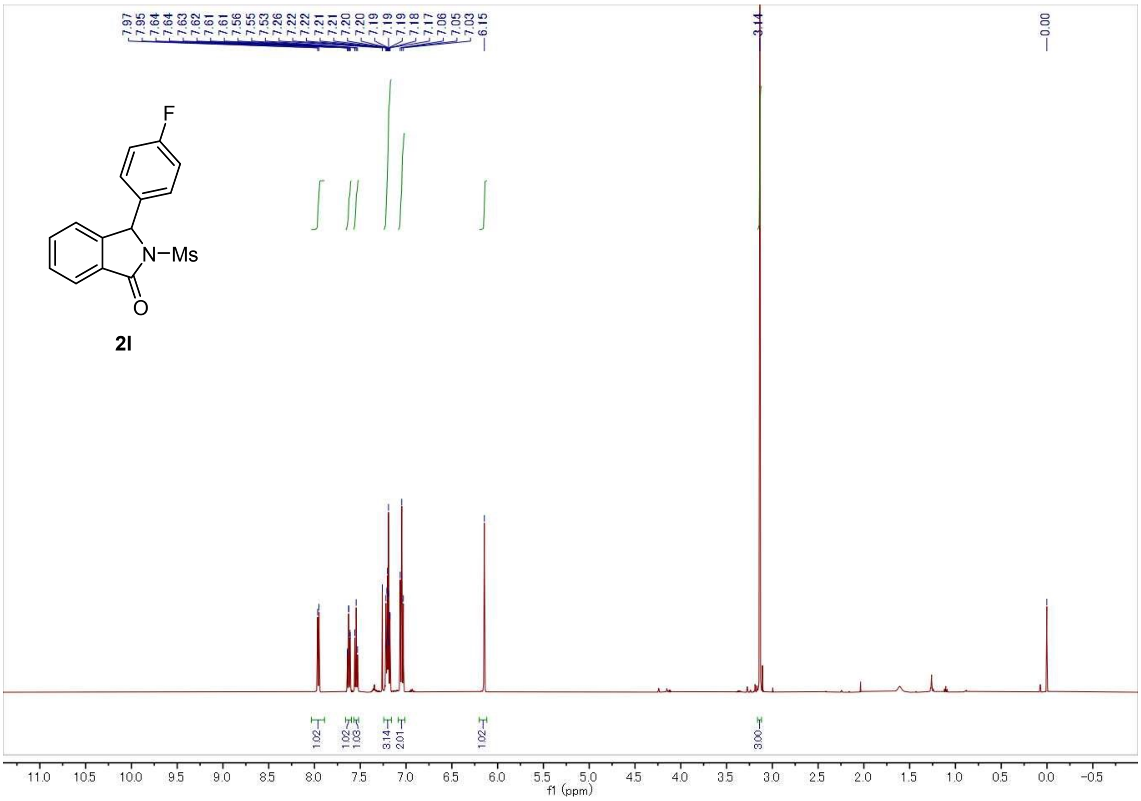

2l

^{13}C NMR (126 MHz, $CDCl_3$) of 2l

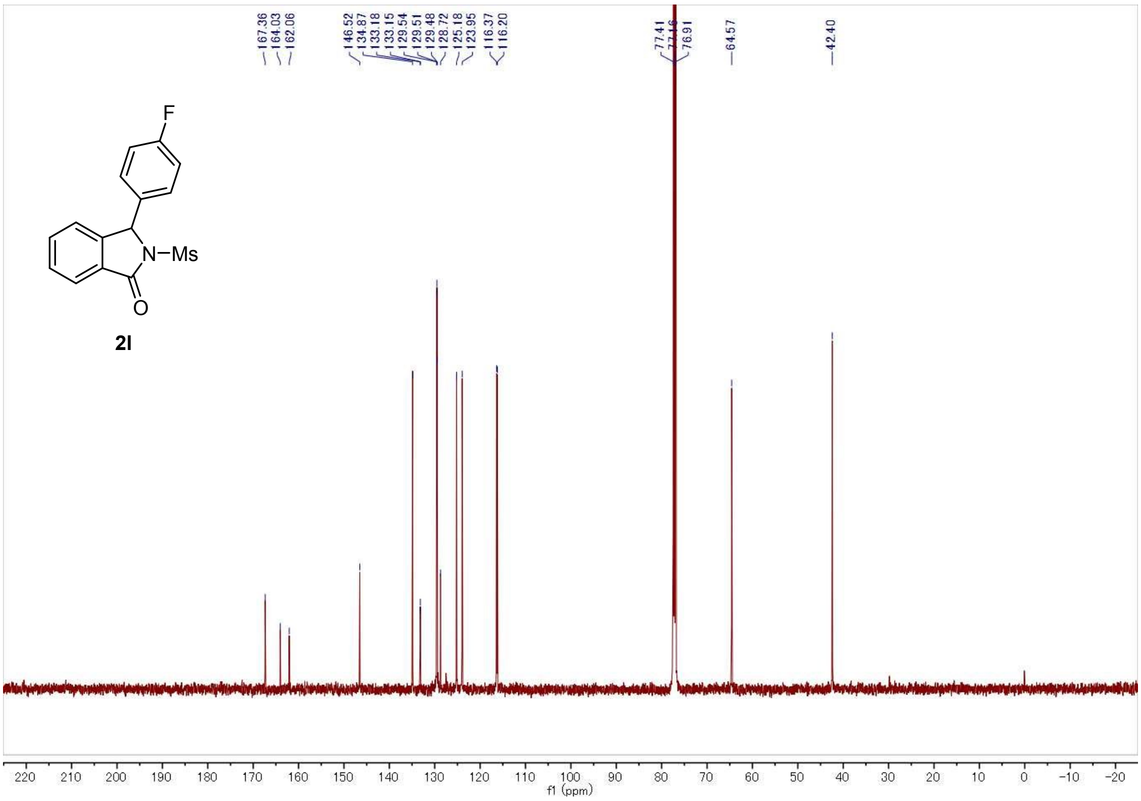

2l

^{19}F NMR (471 MHz, $CDCl_3$) of 2l

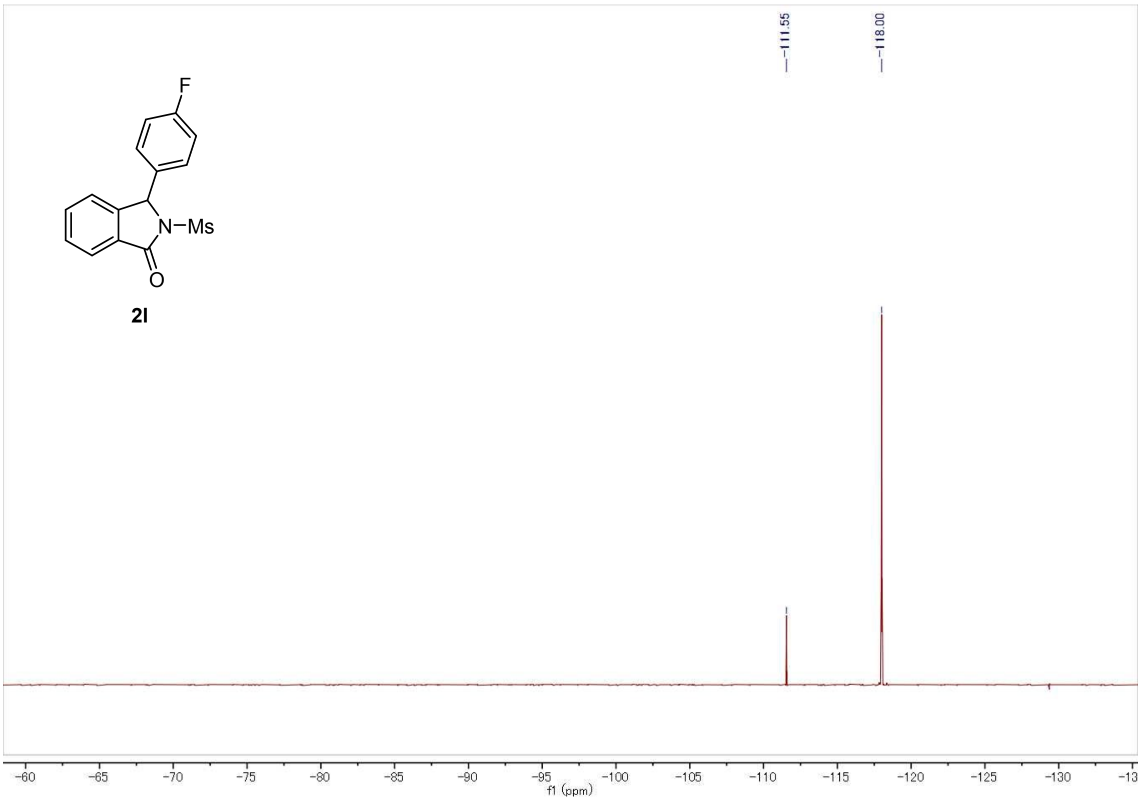

^{1}H NMR (400 MHz, $CDCl_3$) of 2m

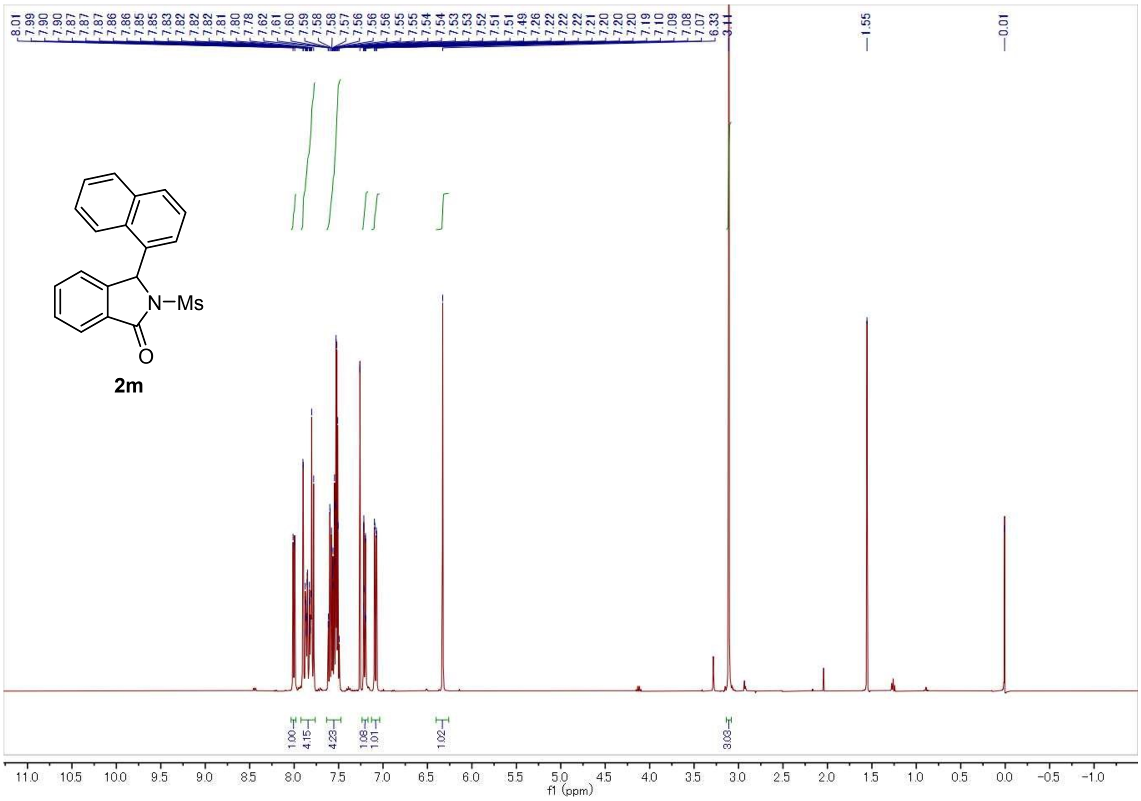

^{13}C NMR (101 MHz, $CDCl_3$) of 2m

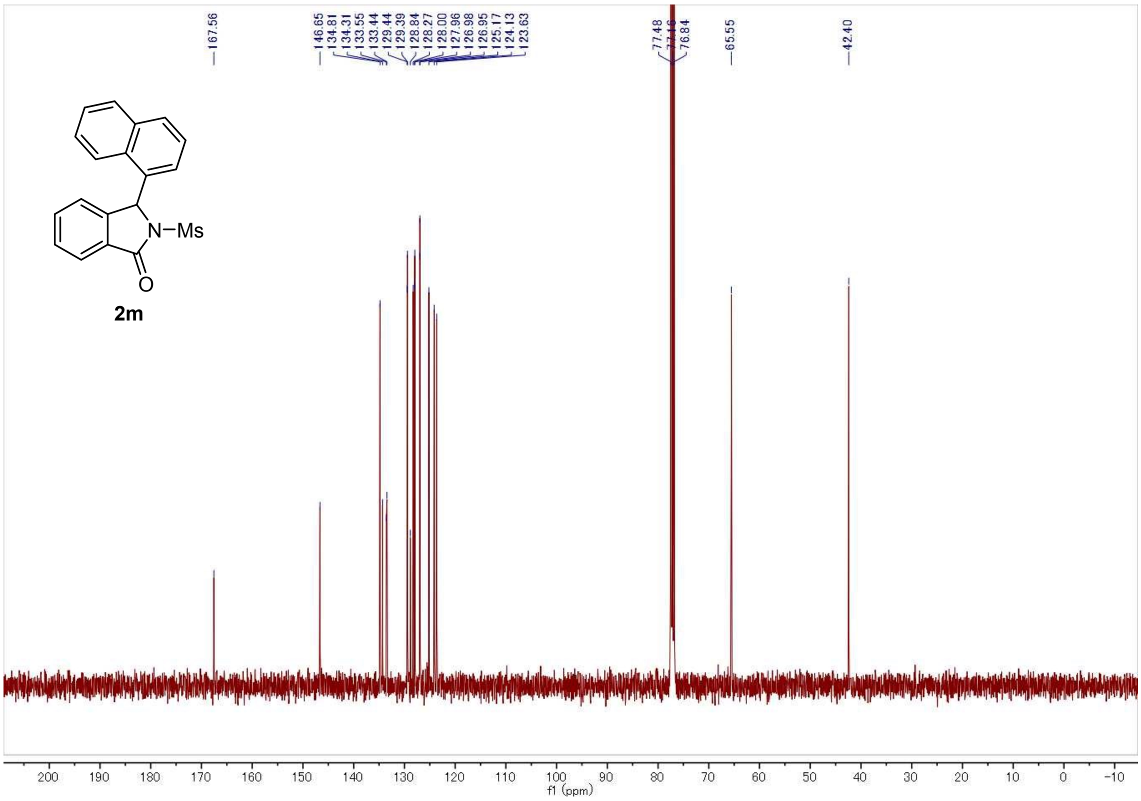

^{1}H NMR (400 MHz, $CDCl_3$) of 2n

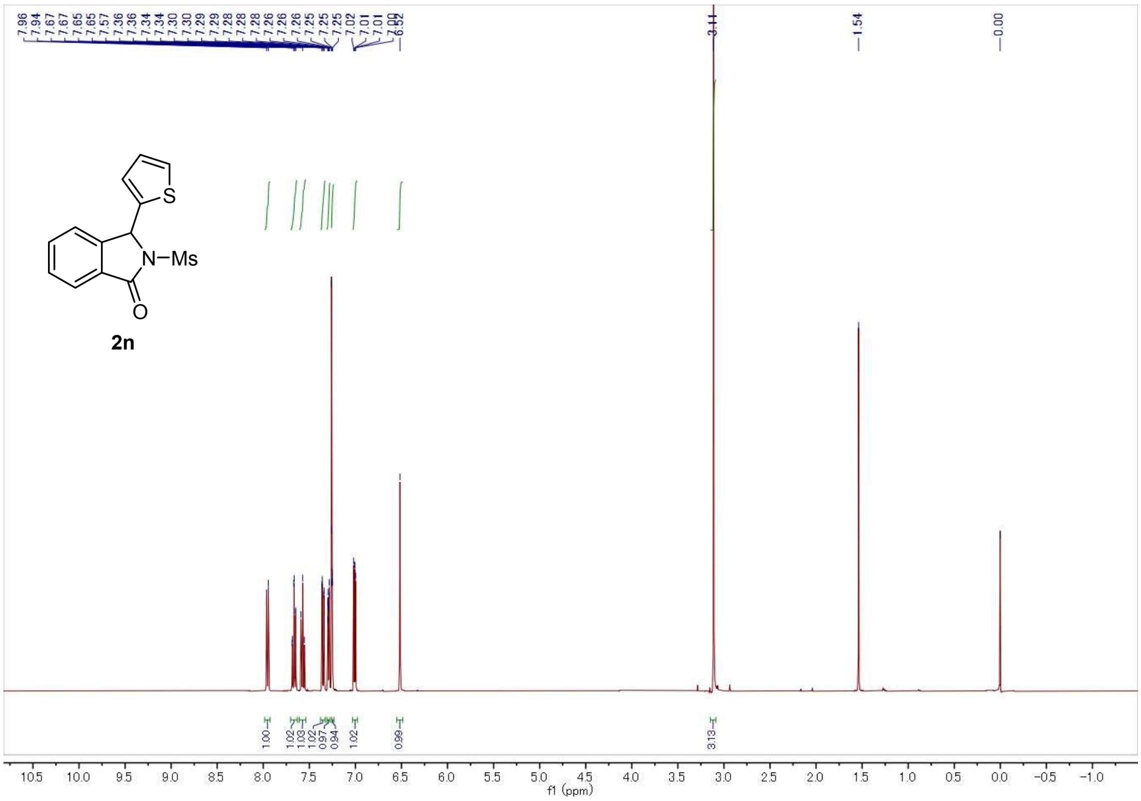

^{13}C NMR (101 MHz, $CDCl_3$) of 2n

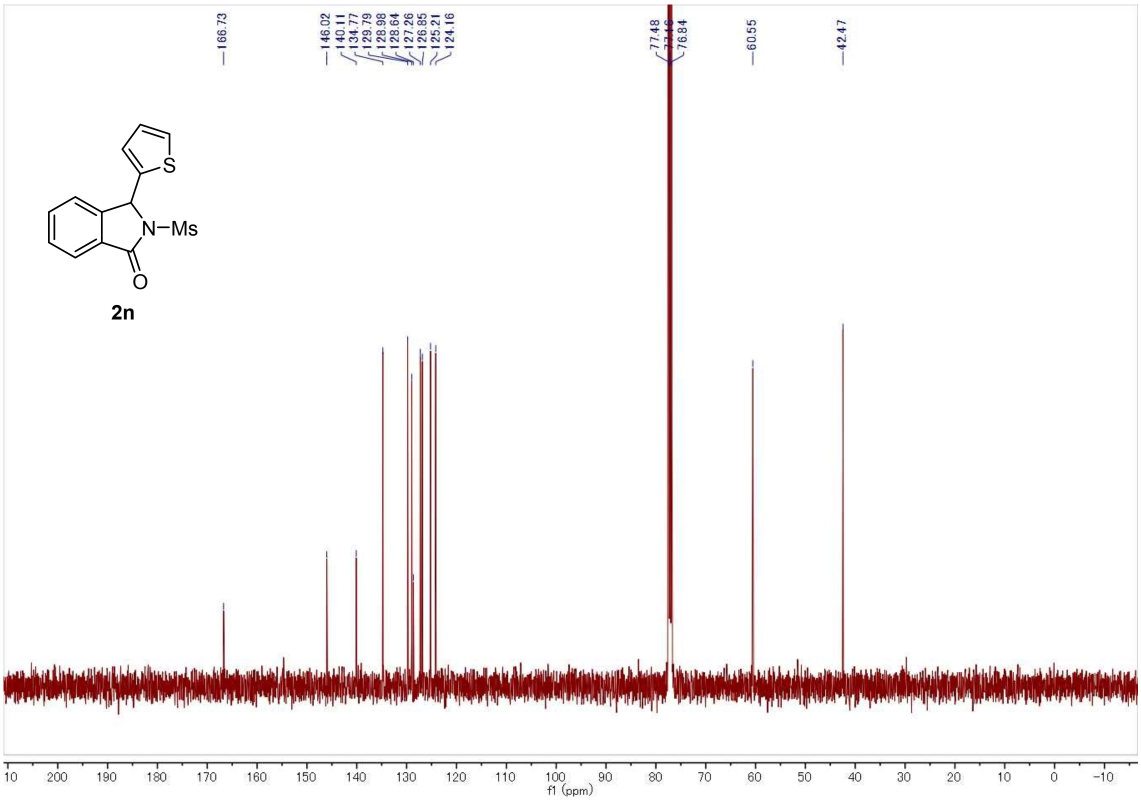

^{1}H NMR (400 MHz, $CDCl_3$) of 2o

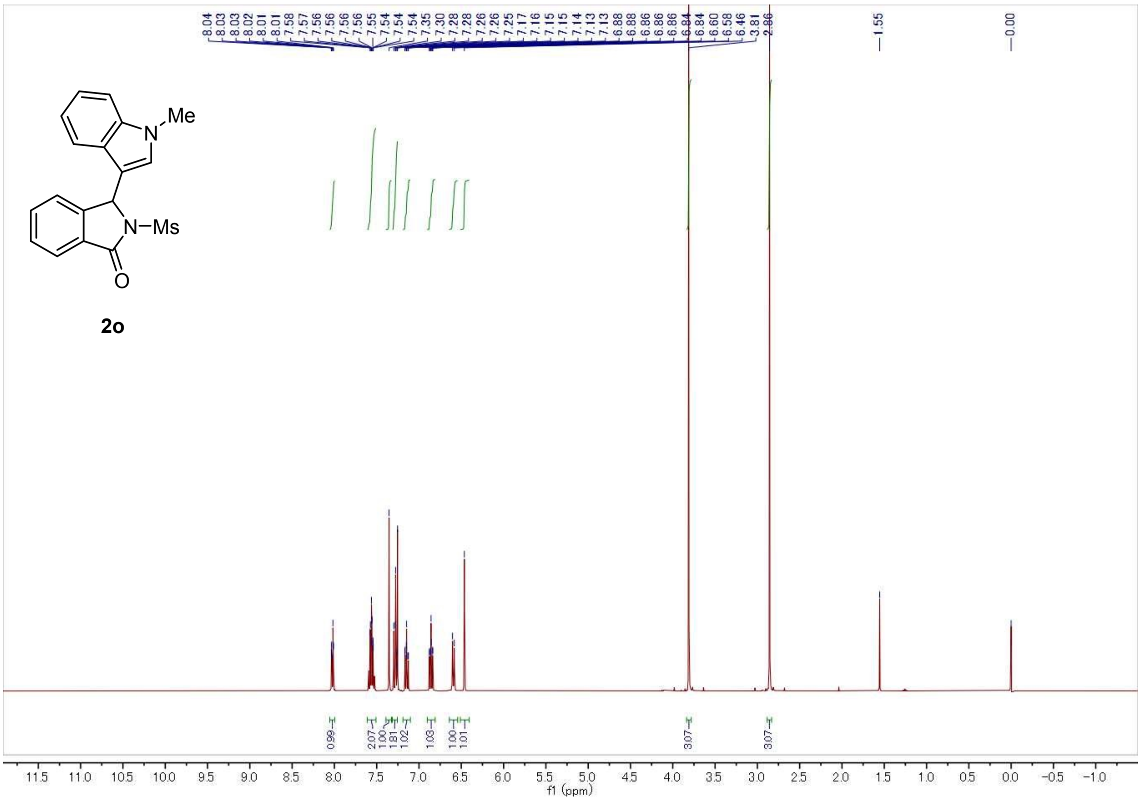

^{13}C NMR (101 MHz, $CDCl_3$) of 2o

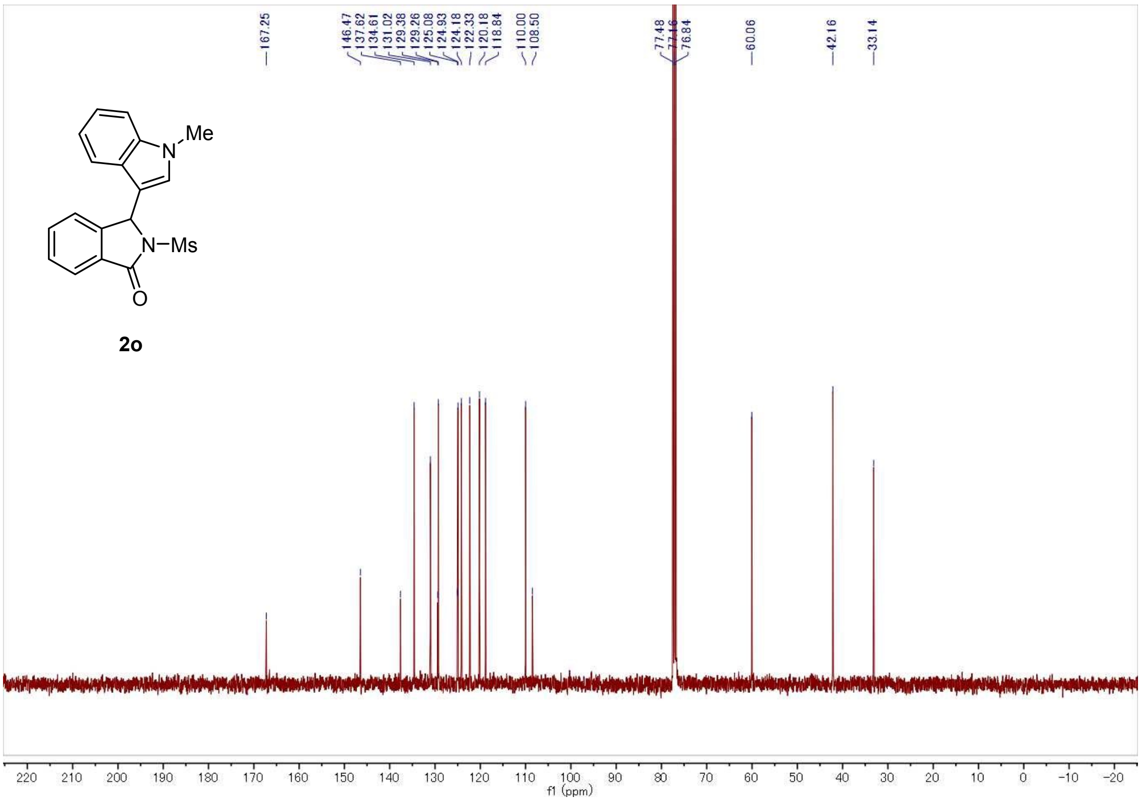

^{1}H NMR (400 MHz, CDCl$_3$) of 2p

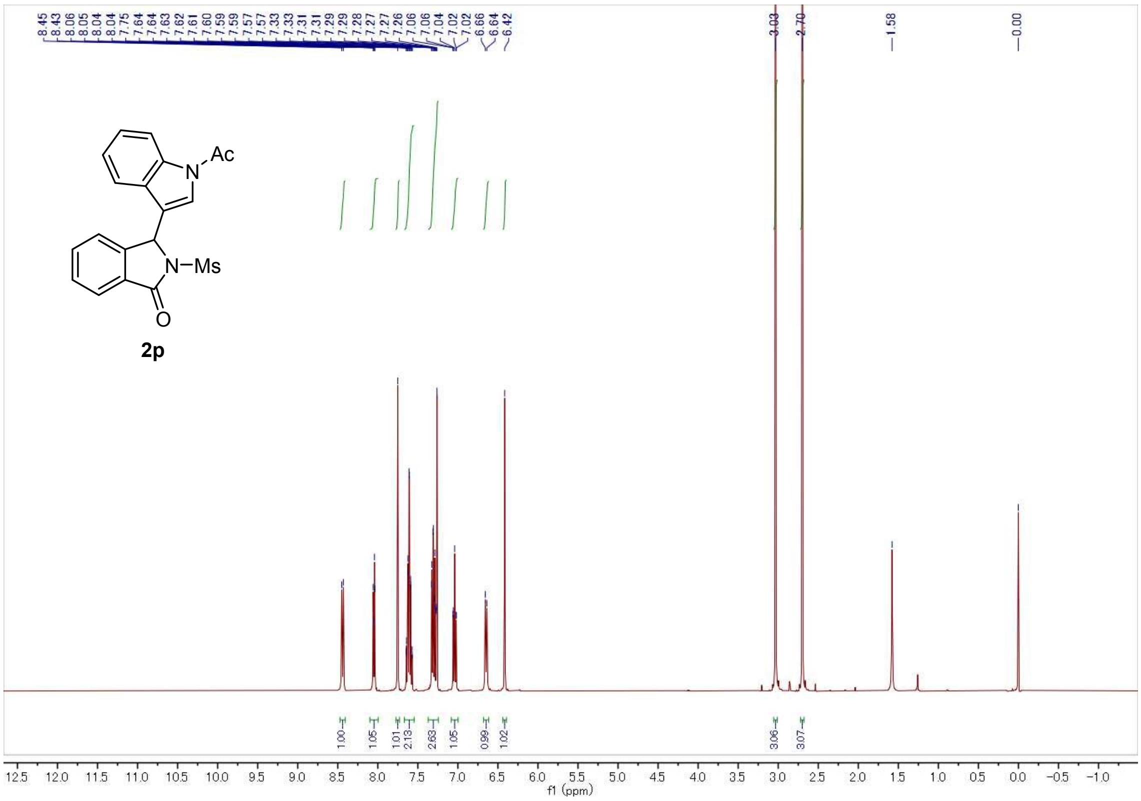

^{13}C NMR (101 MHz, CDCl$_3$) of 2p

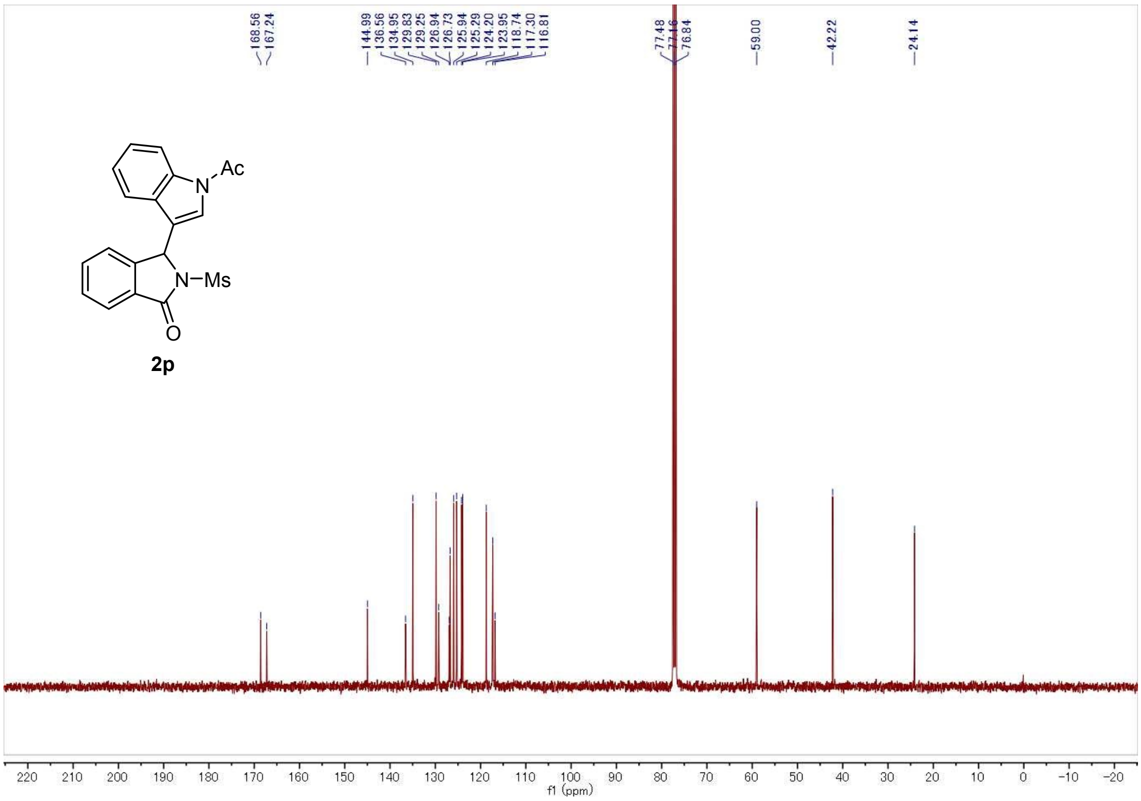

^{1}H NMR (500 MHz, $CDCl_3$) of 2q

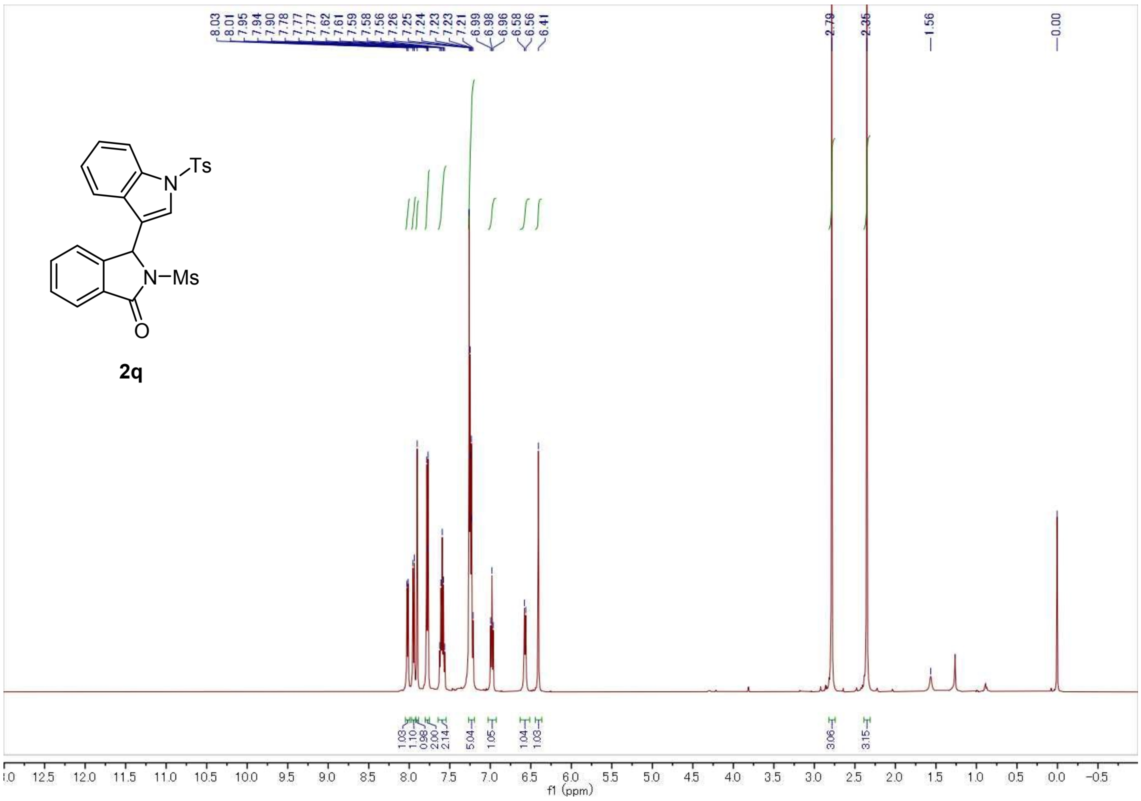

^{13}C NMR (101 MHz, $CDCl_3$) of 2q

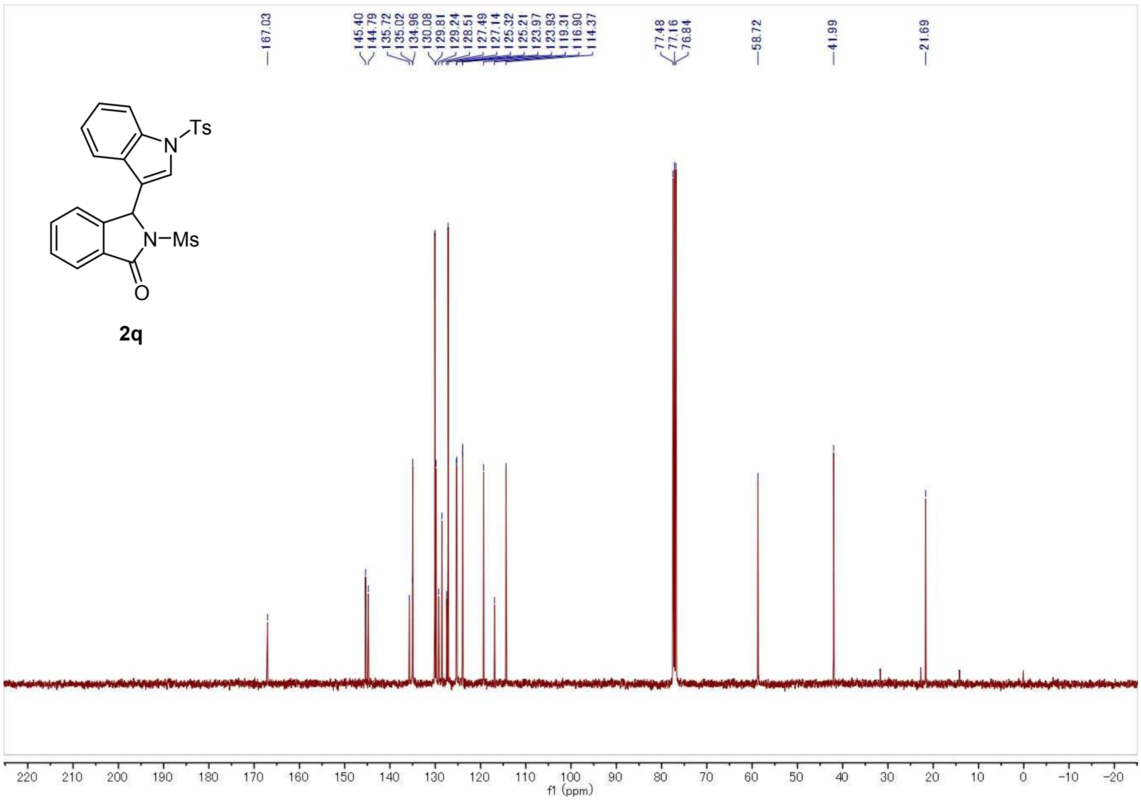

^{1}H NMR (400 MHz, $CDCl_3$) of 3

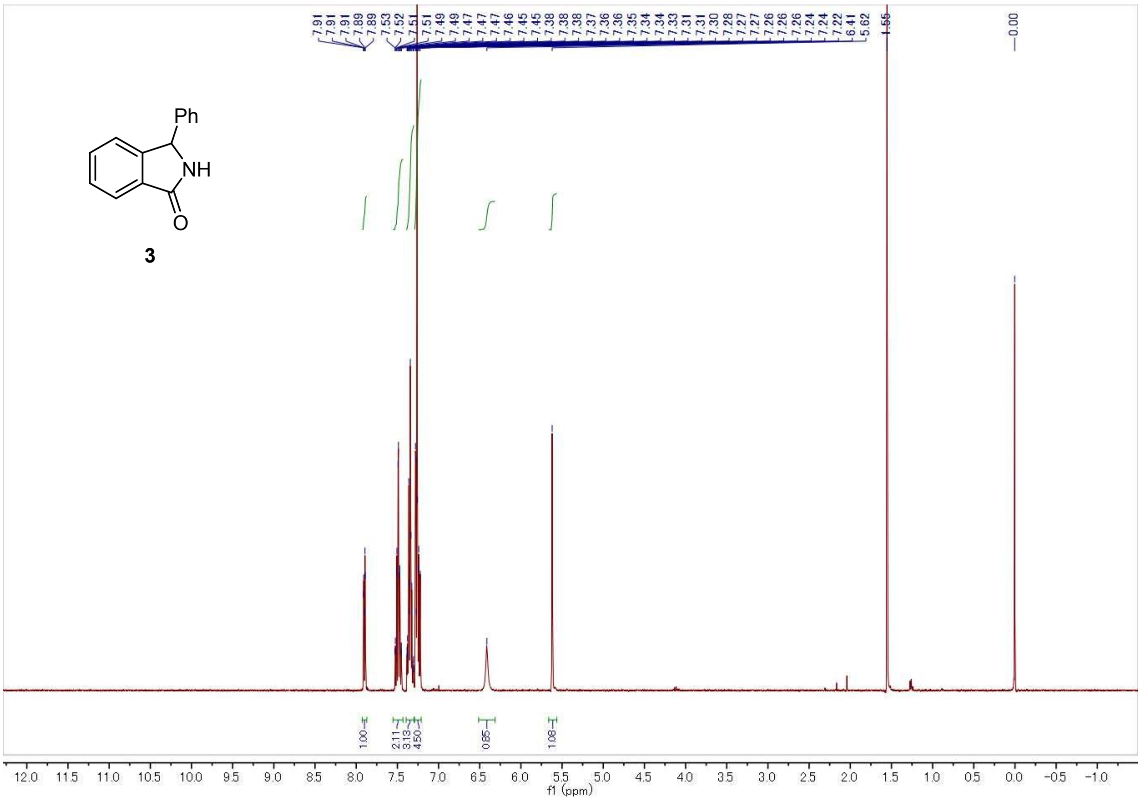

^{13}C NMR (101 MHz, $CDCl_3$) of 3

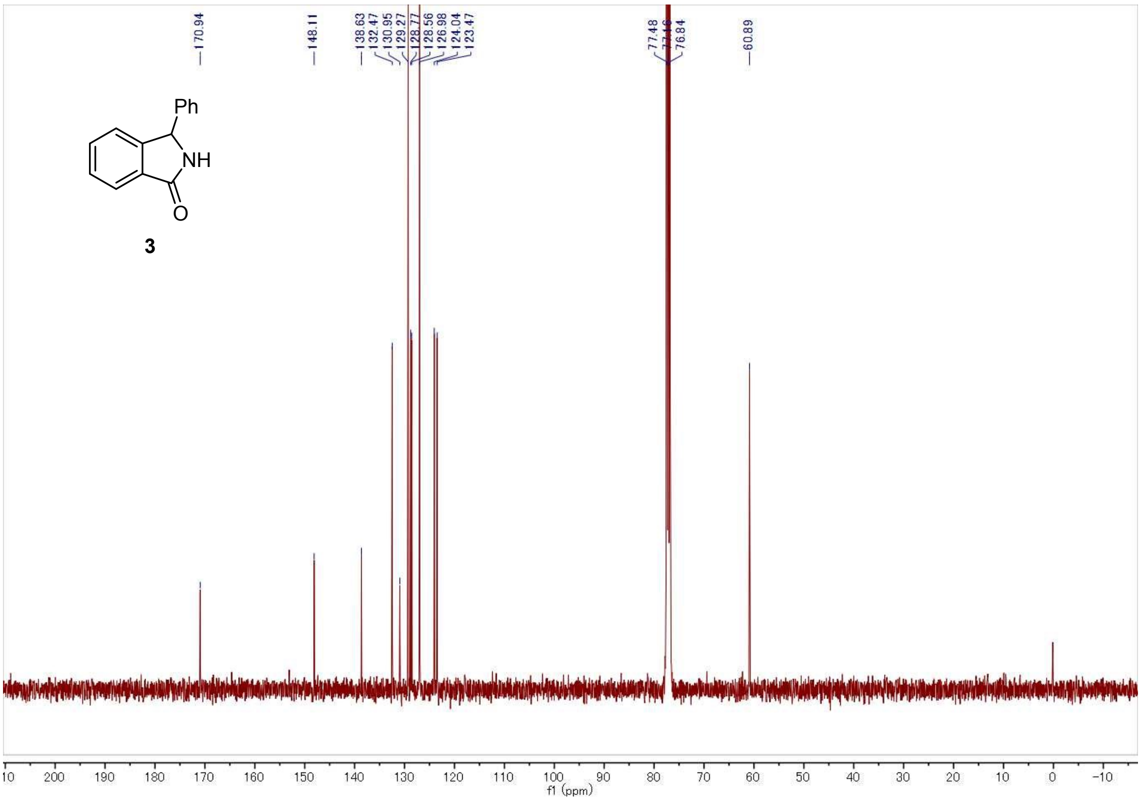

^{1}H NMR (400 MHz, $CDCl_3$) of 4

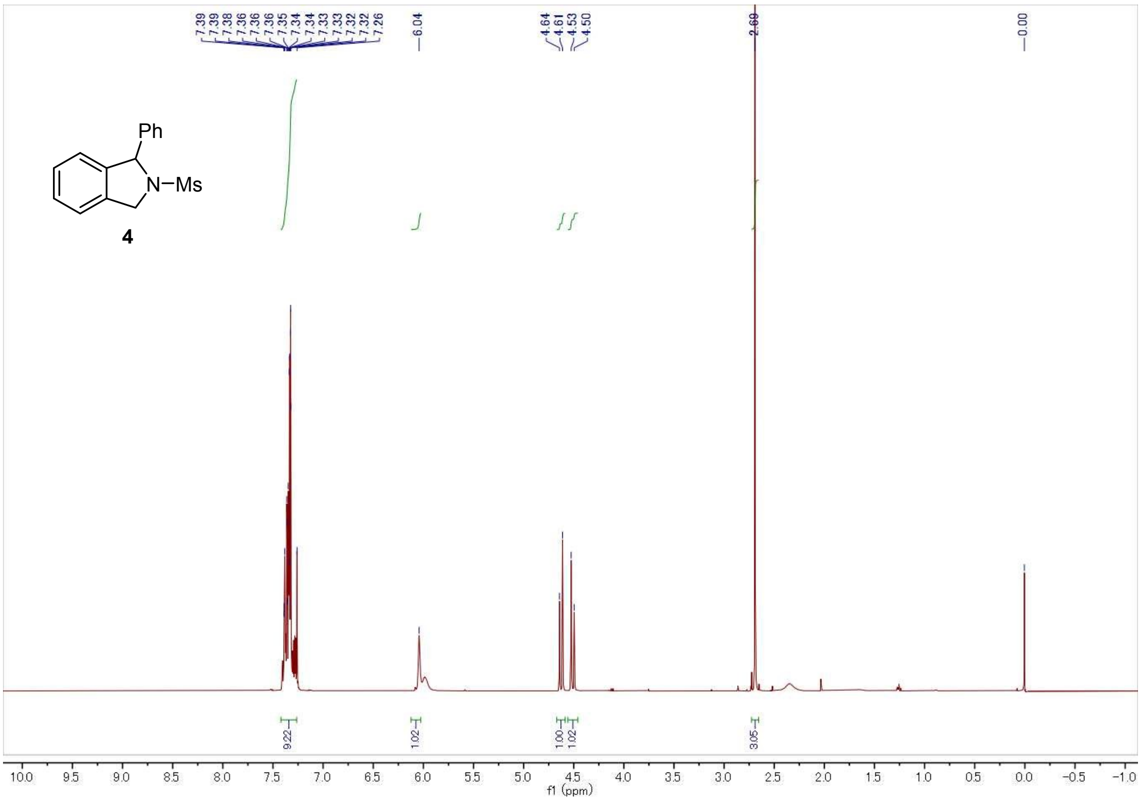

^{13}C NMR (101 MHz, $CDCl_3$) of 4

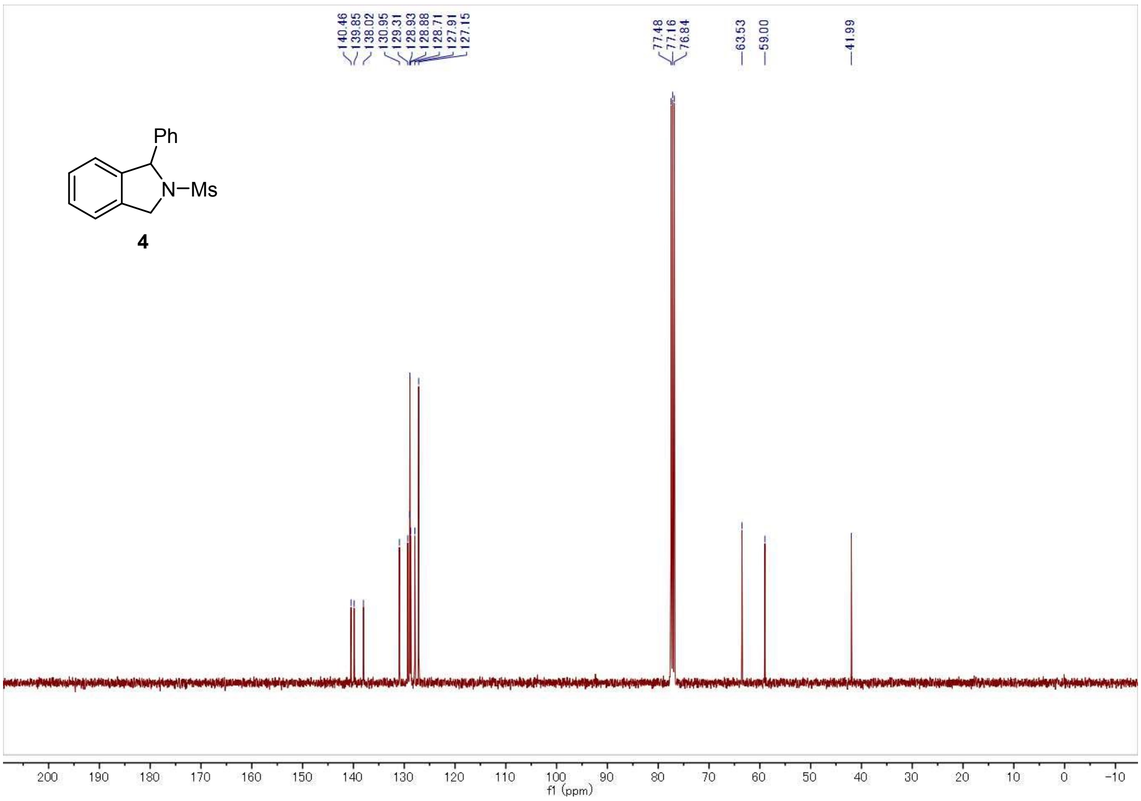

7. Mechanistic Studies

7.1 Confirmation of the Formation of H_2

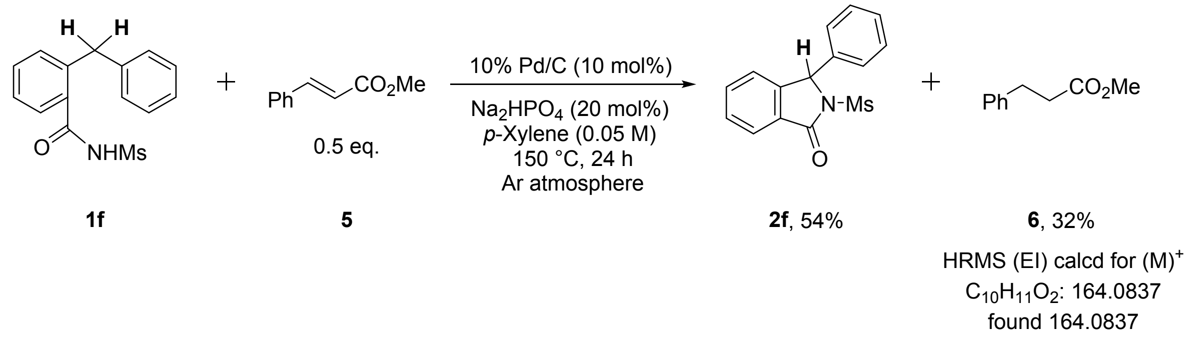

^{1}H-NMR (400 MHz, $CDCl_3$) of the crude mixture after the reaction. 1,1,2-Trichloroethane ($CHCl_2CH_2Cl$) was used as an internal standard.

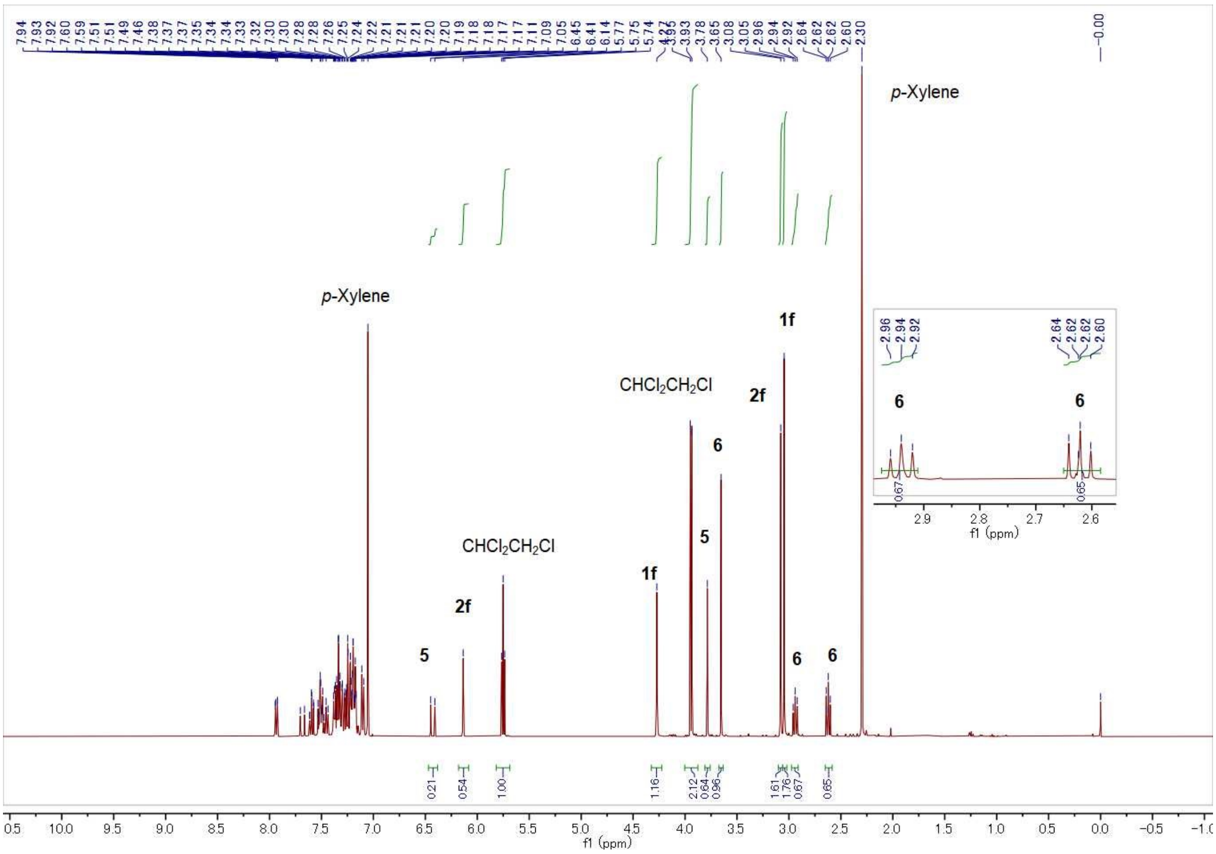

Zoom in (2.4 ppm to 6.8 ppm)

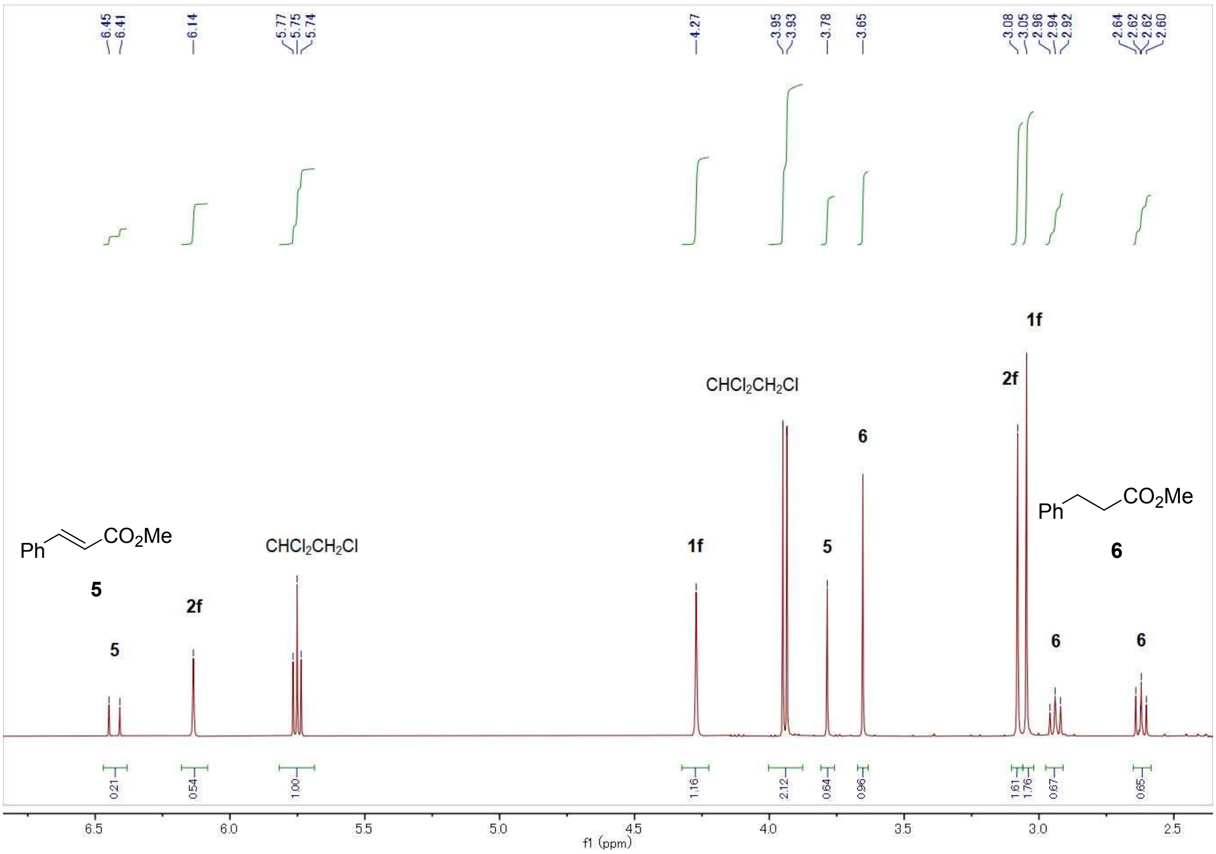

7.2 Palladium Leaching

To confirm the possibility of the palladium leaching, the “hot-filtration test” was examined according to the Glorius’ report (*Angew. Chem. Int. Ed.* **2014**, *53*, 1809).

“Hot-filtration test”: Two reactions of **1f** under the same conditions were independently undertaken. After 2 h heating, one reaction was quenched and the yield was determined by ^{1}H NMR (*control reaction*). The other reaction was directly filtered through a pad of Celite into a test tube containing Na_2HPO_4 (20 mol%). After refilling with Ar, the reaction mixture was heated again for an additional 22 h at 150 °C (*hot filtration test*). No further reaction progress was observed during the additional 22 h (69% yield for 2 h *vs.* 72% yield for 2 + 22 h, the discrepancy between the two yields should be within the measurement error range), suggesting that the active catalytic species are heterogeneous. The result is consistent with that from Glorius’ group.

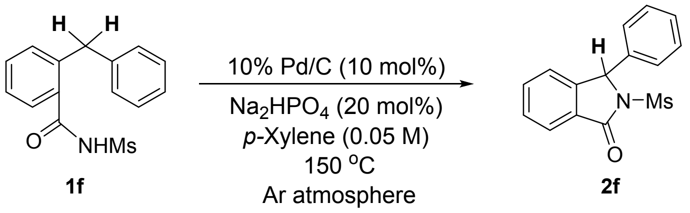

	Yield (2 h)	Yield (24 h)
control reaction	*69%*	92%
hot filtration test	69%	*72%*

7.3 Reuse of Catalyst

According to the Crabtree’s report (*Organometallics* **2002**, *21*, 700), a repetitious process as shown in the scheme below was examined using **1f**, and the desired isoindolinone **2f** was obtained in a high yield.

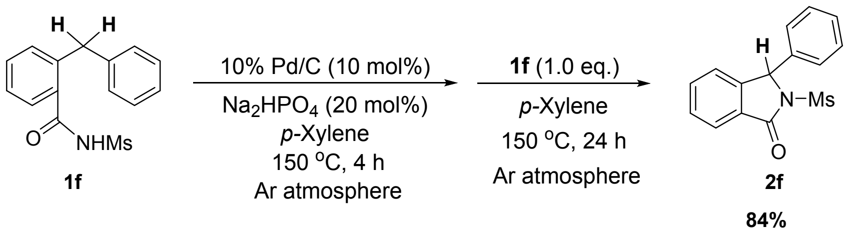

Supplement: RA-011-D1RA04661F-s001 [file RA-011-D1RA04661F-s001.pdf]
